# Diretriz Brasileira sobre a Saúde Cardiovascular no Climatério e na Menopausa – 2024

**DOI:** 10.36660/abc.20240478

**Published:** 2024-07-30

**Authors:** Gláucia Maria Moraes de Oliveira, Maria Cristina Costa de Almeida, Carolina María Artucio Arcelus, Larissa Neto Espíndola, Maria Alayde Mendonça Rivera, Agnaldo Lopes da Silva-Filho, Celi Marques-Santos, César Eduardo Fernandes, Carlos Japhet da Matta Albuquerque, Claudia Maria Vilas Freire, Maria Cristina de Oliveira Izar, Maria Elizabeth Navegantes Caetano Costa, Marildes Luiza de Castro, Viviana de Mello Guzzo Lemke, Alexandre Jorge Gomes de Lucena, Andréa Araujo Brandão, Ariane Vieira Scarlatelli Macedo, Carisi Anne Polanczyk, Carla Janice Baister Lantieri, Eliana Petri Nahas, Elizabeth Regina Giunco Alexandre, Erika Maria Gonçalves Campana, Érika Olivier Vilela Bragança, Fernanda Marciano Consolim Colombo, Imara Correia de Queiroz Barbosa, Ivan Romero Rivera, Jaime Kulak, Lidia Ana Zytynski Moura, Luciano de Mello Pompei, Luiz Francisco Cintra Baccaro, Marcia Melo Barbosa, Marcio Alexandre Hipólito Rodrigues, Marco Aurelio Albernaz, Maria Sotera Paniagua de Decoud, Maria Sanali Moura de Oliveira Paiva, Martha Beatriz Sanchez-Zambrano, Milena dos Santos Barros Campos, Monica Acevedo, Monica Susana Ramirez, Olga Ferreira de Souza, Orlando Otávio de Medeiros, Regina Coeli Marques de Carvalho, Rogerio Bonassi Machado, Sheyla Cristina Tonheiro Ferro da Silva, Thais de Carvalho Vieira Rodrigues, Walkiria Samuel Avila, Lucia Helena Simões da Costa-Paiva, Maria Celeste Osorio Wender

**Affiliations:** 1 Universidade Federal do Rio de Janeiro Rio de Janeiro RJ Brasil Universidade Federal do Rio de Janeiro (UFRJ), Rio de Janeiro, RJ – Brasil; 2 Centro Universitário de Belo Horizonte Belo Horizonte MG Brasil Centro Universitário de Belo Horizonte, Belo Horizonte, MG – Brasil; 3 Centro Cardiovascular de Sanatorio Galicia Montevideo Uruguai Centro Cardiovascular de Sanatorio Galicia, Montevideo – Uruguai; 4 Hospital Santa Izabel Salvador BA Brasil Hospital Santa Izabel, Salvador, BA – Brasil; 5 Hospital Municipal de Salvador Salvador BA Brasil Hospital Municipal de Salvador, Salvador, BA – Brasil; 6 Universidade Federal de Alagoas Maceió AL Brasil Universidade Federal de Alagoas (UFAL), Maceió, AL – Brasil; 7 Universidade Federal de Minas Gerais Belo Horizonte MG Brasil Universidade Federal de Minas Gerais (UFMG), Belo Horizonte, MG – Brasil; 8 Universidade Tiradentes Aracaju SE Brasil Universidade Tiradentes (UNIT), Aracaju, SE – Brasil; 9 Hospital São Lucas Rede D’Or São Luis Aracaju SE Brasil Hospital São Lucas Rede D’Or São Luis, Aracaju, SE – Brasil; 10 Faculdade de Medicina do ABC Santo André SP Brasil Faculdade de Medicina do ABC, Santo André, SP – Brasil; 11 Hospital Santa Joana Recife Recife PE Brasil Hospital Santa Joana Recife, Recife PE – Brasil; 12 EMCOR – Diagnósticos do Coração LTDA Recife PE Brasil EMCOR – Diagnósticos do Coração LTDA, Recife PE – Brasil; 13 Hospital Barão de Lucena Recife PE Brasil Hospital Barão de Lucena, Recife PE – Brasil; 14 Universidade Federal de São Paulo São Paulo SP Brasil Universidade Federal de São Paulo (UNIFESP), São Paulo, SP – Brasil; 15 Centro Universitário do Estado Pará Belém PA Brasil Centro Universitário do Estado Pará (CESUPA), Belém PA – Brasil; 16 Faculdade IPEMED de Ciências Médicas Belo Horizonte MG Brasil Faculdade IPEMED de Ciências Médicas, Belo Horizonte MG – Brasil; 17 Cardiocare Clínica Cardiológica Curitiba PR Brasil Cardiocare Clínica Cardiológica, Curitiba PR – Brasil; 18 Hospital Agamenom Magalhães Recife PE Brasil Hospital Agamenom Magalhães, Recife PE – Brasil; 19 Universidade do Estado do Rio de Janeiro Rio de Janeiro RJ Brasil Universidade do Estado do Rio de Janeiro (UERJ), Rio de Janeiro RJ – Brasil; 20 Santa Casa de Misericórdia de São Paulo São Paulo SP Brasil Santa Casa de Misericórdia de São Paulo, São Paulo, SP – Brasil; 21 Universidade Federal do Rio Grande do Sul Hospital de Clínicas Porto Alegre RS Brasil Hospital de Clínicas da Universidade Federal do Rio Grande do Sul (UFRS), Porto Alegre RS – Brasil; 22 Hospital do Coração São Paulo SP Brasil Hospital do Coração (HCor), São Paulo SP – Brasil; 23 RitmoCheck São José dos Campos SP Brasil RitmoCheck, São José dos Campos, SP – Brasil; 24 Instituto do Coração Hospital das Clínicas FMUSP São Paulo SP Brasil Instituto do Coração (Incor) do Hospital das Clínicas FMUSP, São Paulo SP – Brasil; 25 Universidade Federal de Campina Grande Campina Grande PB Brasil Universidade Federal de Campina Grande, Campina Grande, PB – Brasil; 26 Universidade Federal de Alagoas Maceio AL Brasil Universidade Federal de Alagoas (UFAL), Maceio AL – Brasil; 27 Maceió AL – BrasilUniversidade Federal do Paraná Curitiba PR Brasil Maceió AL – BrasilUniversidade Federal do Paraná (UFPR), Curitiba, PR – Brasil; 28 Pontifícia Universidade Católica do Paraná Curitiba PR Brasil Pontifícia Universidade Católica do Paraná (PUC-PR), Curitiba, PR – Brasil; 29 Universidade Estadual de Campinas Campinas SP Brasil Universidade Estadual de Campinas (UNICAMP), Campinas, SP – Brasil; 30 Hospital Socor Belo Horizonte MG Brasil Hospital Socor, Belo Horizonte, MG – Brasil; 31 Hospital Estadual da Mulher Goiânia GO Brasil Hospital Estadual da Mulher, Goiânia, GO – Brasil; 32 Sanatorio Italiano Assunção Paraguai Sanatorio Italiano, Assunção – Paraguai; 33 Universidade Federal do Rio Grande do Norte Natal RN Brasil Universidade Federal do Rio Grande do Norte, Natal, RN – Brasil; 34 Comité de Enfermedades Cardiovasculares de la Mujer Sociedad Venezolana de Cardiología Caracas Venezuela Comité de Enfermedades Cardiovasculares de la Mujer, Sociedad Venezolana de Cardiología, Caracas – Venezuela; 35 Hospital São Lucas Rede D’Or São Luiz Aracaju SE Brasil Hospital São Lucas, Rede D’Or São Luiz, Aracaju, SE – Brasil; 36 Pontificia Universidad Católica de Chile Santiago Chile Pontificia Universidad Católica de Chile, Santiago – Chile; 37 Hospital Privado Rosario Rosario Argentina Hospital Privado Rosario, Rosario – Argentina; 38 Instituto Universitario Rosario Santa Fe Argentina Instituto Universitario Rosario (IUNIR), Santa Fe – Argentina; 39 Rede D’Or Rio de Janeiro RJ Brasil Rede D’Or, Rio de Janeiro, RJ – Brasil; 40 Ministério da Saúde Brasília DF Brasil Ministério da Saúde, Brasília, DF – Brasil; 41 Hospital Geral de Fortaleza Fortaleza CE Brasil Hospital Geral de Fortaleza, Fortaleza CE – Brasil; 42 Secretaria de Saúde do Estado do Ceará Fortaleza CE Brasil Secretaria de Saúde do Estado do Ceará, Fortaleza CE – Brasil; 43 Faculdade de Medicina de Jundiaí Jundiaí SP Brasil Faculdade de Medicina de Jundiaí, Jundiaí, SP – Brasil; 44 CEMISE Oncoclínicas Aracaju SE Brasil CEMISE Oncoclínicas, Aracaju, SE – Brasil; 45 Universidade Federal de Sergipe Aracaju SE Brasil Universidade Federal de Sergipe (UFS), Aracaju, SE – Brasil; 46 Hospital de Clínicas de Porto Alegre Porto Alegre RS Brasil Hospital de Clínicas de Porto Alegre, Porto Alegre, RS – Brasil

**Table:** 

Diretriz Brasileira sobre a Saúde Cardiovascular no Climatério e na Menopausa – 2024	
O relatório abaixo lista as declarações de interesse conforme relatadas à SBC pelos especialistas durante o período de desenvolvimento deste posicionamento, 2023-2024.	
Especialista	Tipo de relacionamento com a indústria
Agnaldo Lopes da Silva-Filho	Outros relacionamentos Financiamento de atividades de educação médica continuada, incluindo viagens, hospedagens e inscrições para congressos e cursos, provenientes da indústria farmacêutica, de órteses, próteses, equipamentos e implantes, brasileiras ou estrangeiras: - Bayer e Vifor.
Alexandre Jorge Gomes de Lucena	Nada a ser declarado
Andréa Araujo Brandão	Nada a ser declarado
Ariane Vieira Scarlatelli Macedo	Declaração financeira A - Pagamento de qualquer espécie e desde que economicamente apreciáveis, feitos a (i) você, (ii) ao seu cônjuge/companheiro ou a qualquer outro membro que resida com você, (iii) a qualquer pessoa jurídica em que qualquer destes seja controlador, sócio, acionista ou participante, de forma direta ou indireta, recebimento por palestras, aulas, atuação como proctor de treinamentos, remunerações, honorários pagos por participações em conselhos consultivos, de investigadores, ou outros comitês, etc. Provenientes da indústria farmacêutica, de órteses, próteses, equipamentos e implantes, brasileiras ou estrangeiras: - Bayer: anticoagulação e insuficiência cardíaca; Pfizer: anticoagulação e amiloidose; Jannsen: leucemia. Outros relacionamentos Financiamento de atividades de educação médica continuada, incluindo viagens, hospedagens e inscrições para congressos e cursos, provenientes da indústria farmacêutica, de órteses, próteses, equipamentos e implantes, brasileiras ou estrangeiras: - Bayer: insuficiência cardíaca.
Carisi Anne Polanczyk	Nada a ser declarado
Carla Janice Baister Lantieri	Nada a ser declarado
Carlos Japhet da Matta Albuquerque	Nada a ser declarado
Carolina María Artucio Arcelus	Nada a ser declarado
Celi Marques-Santos	Nada a ser declarado
César Eduardo Fernandes	Declaração financeira A - Pagamento de qualquer espécie e desde que economicamente apreciáveis, feitos a (i) você, (ii) ao seu cônjuge/ companheiro ou a qualquer outro membro que resida com você, (iii) a qualquer pessoa jurídica em que qualquer destes seja controlador, sócio, acionista ou participante, de forma direta ou indireta, recebimento por palestras, aulas, atuação como proctor de treinamentos, remunerações, honorários pagos por participações em conselhos consultivos, de investigadores, ou outros comitês, etc. Provenientes da indústria farmacêutica, de órteses, próteses, equipamentos e implantes, brasileiras ou estrangeiras: - Membro do Board e palestrante dos laboratórios GENON, Bayer, Sanofi, Theramex.
Claudia Maria Vilas Freire	Nada a ser declarado
Eliana Aguiar Petri Nahas	Declaração financeira A - Pagamento de qualquer espécie e desde que economicamente apreciáveis, feitos a (i) você, (ii) ao seu cônjuge/ companheiro ou a qualquer outro membro que resida com você, (iii) a qualquer pessoa jurídica em que qualquer destes seja controlador, sócio, acionista ou participante, de forma direta ou indireta, recebimento por palestras, aulas, atuação como proctor de treinamentos, remunerações, honorários pagos por participações em conselhos consultivos, de investigadores, ou outros comitês, etc. Provenientes da indústria farmacêutica, de órteses, próteses, equipamentos e implantes, brasileiras ou estrangeiras: - Libbs: Iziz, IUMI ES; Theramex: Systen adesivos, Estreva Gel.
Elizabeth Regina Giunco Alexandre	Declaração financeira A - Pagamento de qualquer espécie e desde que economicamente apreciáveis, feitos a (i) você, (ii) ao seu cônjuge/ companheiro ou a qualquer outro membro que resida com você, (iii) a qualquer pessoa jurídica em que qualquer destes seja controlador, sócio, acionista ou participante, de forma direta ou indireta, recebimento por palestras, aulas, atuação como proctor de treinamentos, remunerações, honorários pagos por participações em conselhos consultivos, de investigadores, ou outros comitês, etc. Provenientes da indústria farmacêutica, de órteses, próteses, equipamentos e implantes, brasileiras ou estrangeiras: - Lilly, Servier, Libbs.
Erika Maria Gonçalves Campana	Declaração financeira A - Pagamento de qualquer espécie e desde que economicamente apreciáveis, feitos a (i) você, (ii) ao seu cônjuge/ companheiro ou a qualquer outro membro que resida com você, (iii) a qualquer pessoa jurídica em que qualquer destes seja controlador, sócio, acionista ou participante, de forma direta ou indireta, recebimento por palestras, aulas, atuação como proctor de treinamentos, remunerações, honorários pagos por participações em conselhos consultivos, de investigadores, ou outros comitês, etc. Provenientes da indústria farmacêutica, de órteses, próteses, equipamentos e implantes, brasileiras ou estrangeiras: - Servier, Brace Pharma, Biolab, Momenta. Outros relacionamentos Financiamento de atividades de educação médica continuada, incluindo viagens, hospedagens e inscrições para congressos e cursos, provenientes da indústria farmacêutica, de órteses, próteses, equipamentos e implantes, brasileiras ou estrangeiras: - Servier: hipertensão; Biolab: hipertensão.
Érika Olivier Vilela Bragança	Declaração financeira A - Pagamento de qualquer espécie e desde que economicamente apreciáveis, feitos a (i) você, (ii) ao seu cônjuge/ companheiro ou a qualquer outro membro que resida com você, (iii) a qualquer pessoa jurídica em que qualquer destes seja controlador, sócio, acionista ou participante, de forma direta ou indireta, recebimento por palestras, aulas, atuação como proctor de treinamentos, remunerações, honorários pagos por participações em conselhos consultivos, de investigadores, ou outros comitês, etc. Provenientes da indústria farmacêutica, de órteses, próteses, equipamentos e implantes, brasileiras ou estrangeiras: - Biolab: Dozoito. Outros relacionamentos Financiamento de atividades de educação médica continuada, incluindo viagens, hospedagens e inscrições para congressos e cursos, provenientes da indústria farmacêutica, de órteses, próteses, equipamentos e implantes, brasileiras ou estrangeiras: - Merck.
Fernanda Marciano Consolim Colombo	Declaração financeira A - Pagamento de qualquer espécie e desde que economicamente apreciáveis, feitos a (i) você, (ii) ao seu cônjuge/ companheiro ou a qualquer outro membro que resida com você, (iii) a qualquer pessoa jurídica em que qualquer destes seja controlador, sócio, acionista ou participante, de forma direta ou indireta, recebimento por palestras, aulas, atuação como proctor de treinamentos, remunerações, honorários pagos por participações em conselhos consultivos, de investigadores, ou outros comitês, etc. Provenientes da indústria farmacêutica, de órteses, próteses, equipamentos e implantes, brasileiras ou estrangeiras: - Daiichi Sankyo; Merck; Servier; AstraZeneca. Outros relacionamentos Financiamento de atividades de educação médica continuada, incluindo viagens, hospedagens e inscrições para congressos e cursos, provenientes da indústria farmacêutica, de órteses, próteses, equipamentos e implantes, brasileiras ou estrangeiras: - Daiichi Sankyo; Servier.
Gláucia Maria Moraes de Oliveira	Nada a ser declarado
Imara Correia de Queiroz Barbosa	Nada a ser declarado
Ivan Romero Rivera	Nada a ser declarado
Jaime Kulak Junior	Nada a ser declarado
Larissa Neto Espíndola	Nada a ser declarado
Lidia Ana Zytynski Moura	Declaração financeira A - Pagamento de qualquer espécie e desde que economicamente apreciáveis, feitos a (i) você, (ii) ao seu cônjuge/companheiro ou a qualquer outro membro que resida com você, (iii) a qualquer pessoa jurídica em que qualquer destes seja controlador, sócio, acionista ou participante, de forma direta ou indireta, recebimento por palestras, aulas, atuação como proctor de treinamentos, remunerações, honorários pagos por participações em conselhos consultivos, de investigadores, ou outros comitês, etc. Provenientes da indústria farmacêutica, de órteses, próteses, equipamentos e implantes, brasileiras ou estrangeiras: - Novartis: Entresto; AstraZeneca: Forxiga; Boehringer: Jardiance; Bayer: Vericiguat; Vifor: Ferrinject. B - Financiamento de pesquisas sob sua responsabilidade direta/pessoal (direcionado ao departamento ou instituição) provenientes da indústria farmacêutica, de órteses, próteses, equipamentos e implantes, brasileiras ou estrangeiras. - Bayer: Vericiguat.
Lucia Helena Simões da Costa-Paiva	Declaração financeira A - Pagamento de qualquer espécie e desde que economicamente apreciáveis, feitos a (i) você, (ii) ao seu cônjuge/companheiro ou a qualquer outro membro que resida com você, (iii) a qualquer pessoa jurídica em que qualquer destes seja controlador, sócio, acionista ou participante, de forma direta ou indireta, recebimento por palestras, aulas, atuação como proctor de treinamentos, remunerações, honorários pagos por participações em conselhos consultivos, de investigadores, ou outros comitês, etc. Provenientes da indústria farmacêutica, de órteses, próteses, equipamentos e implantes, brasileiras ou estrangeiras: - Bayer: Siu Mirena; Besins: Vagifem; Libbs: terapia hormonal e contracepção; Theramex: terapia hormonal. B - Financiamento de pesquisas sob sua responsabilidade direta/pessoal (direcionado ao departamento ou instituição) provenientes da indústria farmacêutica, de órteses, próteses, equipamentos e implantes, brasileiras ou estrangeiras. - FAPESP e bolsa produtiva CNPQ. Outros relacionamentos Financiamento de atividades de educação médica continuada, incluindo viagens, hospedagens e inscrições para congressos e cursos, provenientes da indústria farmacêutica, de órteses, próteses, equipamentos e implantes, brasileiras ou estrangeiras: - Besins: Vagifem; Bayer: Mirena; Astelas: Fezolinetanto.
Luciano de Melo Pompei	Declaração financeira A - Pagamento de qualquer espécie e desde que economicamente apreciáveis, feitos a (i) você, (ii) ao seu cônjuge/ companheiro ou a qualquer outro membro que resida com você, (iii) a qualquer pessoa jurídica em que qualquer destes seja controlador, sócio, acionista ou participante, de forma direta ou indireta, recebimento por palestras, aulas, atuação como proctor de treinamentos, remunerações, honorários pagos por participações em conselhos consultivos, de investigadores, ou outros comitês, etc. Provenientes da indústria farmacêutica, de órteses, próteses, equipamentos e implantes, brasileiras ou estrangeiras: - Abbott; Besins; Libbs; Theramex: produtos de terapêutica hormonal da menopausa.
Luiz Francisco Cintra Baccaro	Nada a ser declarado
Marcia Melo barbosa	Nada a ser declarado
Marcio Alexandre Hipólito Rodrigues	Declaração financeira A - Pagamento de qualquer espécie e desde que economicamente apreciáveis, feitos a (i) você, (ii) ao seu cônjuge/ companheiro ou a qualquer outro membro que resida com você, (iii) a qualquer pessoa jurídica em que qualquer destes seja controlador, sócio, acionista ou participante, de forma direta ou indireta, recebimento por palestras, aulas, atuação como proctor de treinamentos, remunerações, honorários pagos por participações em conselhos consultivos, de investigadores, ou outros comitês, etc. Provenientes da indústria farmacêutica, de órteses, próteses, equipamentos e implantes, brasileiras ou estrangeiras: - Theramex, Besins, Novonordisk. B - Financiamento de pesquisas sob sua responsabilidade direta/pessoal (direcionado ao departamento ou instituição) provenientes da indústria farmacêutica, de órteses, próteses, equipamentos e implantes, brasileiras ou estrangeiras: - Theramex. Outros relacionamentos Financiamento de atividades de educação médica continuada, incluindo viagens, hospedagens e inscrições para congressos e cursos, provenientes da indústria farmacêutica, de órteses, próteses, equipamentos e implantes, brasileiras ou estrangeiras: - Theramex e Besins
Marco Aurélio Albernaz	Declaração financeira A - Pagamento de qualquer espécie e desde que economicamente apreciáveis, feitos a (i) você, (ii) ao seu cônjuge/ companheiro ou a qualquer outro membro que resida com você, (iii) a qualquer pessoa jurídica em que qualquer destes seja controlador, sócio, acionista ou participante, de forma direta ou indireta, recebimento por palestras, aulas, atuação como proctor de treinamentos, remunerações, honorários pagos por participações em conselhos consultivos, de investigadores, ou outros comitês, etc. Provenientes da indústria farmacêutica, de órteses, próteses, equipamentos e implantes, brasileiras ou estrangeiras: - Bayer: anticoncepção; Theramex: anticoncepção e terapia hormonal; Organon: anticoncepção; Libbs: terapia hormonal.
Maria Alayde Mendonça Rivera	Nada a ser declarado
Maria Celeste Osorio Wender	Declaração financeira A - Pagamento de qualquer espécie e desde que economicamente apreciáveis, feitos a (i) você, (ii) ao seu cônjuge/ companheiro ou a qualquer outro membro que resida com você, (iii) a qualquer pessoa jurídica em que qualquer destes seja controlador, sócio, acionista ou participante, de forma direta ou indireta, recebimento por palestras, aulas, atuação como proctor de treinamentos, remunerações, honorários pagos por participações em conselhos consultivos, de investigadores, ou outros comitês, etc. Provenientes da indústria farmacêutica, de órteses, próteses, equipamentos e implantes, brasileiras ou estrangeiras: - Bayer, Besins, MSD, Astellas, Gedeon, Abbott, Theramex. B - Financiamento de pesquisas sob sua responsabilidade direta/pessoal (direcionado ao departamento ou instituição) provenientes da indústria farmacêutica, de órteses, próteses, equipamentos e implantes, brasileiras ou estrangeiras: - Mithra: Estetrol; Myovant: Relugolix.
Maria Cristina Costa de Almeida	Nada a ser declarado
Maria Cristina de Oliveira Izar	Declaração financeira A - Pagamento de qualquer espécie e desde que economicamente apreciáveis, feitos a (i) você, (ii) ao seu cônjuge/companheiro ou a qualquer outro membro que resida com você, (iii) a qualquer pessoa jurídica em que qualquer destes seja controlador, sócio, acionista ou participante, de forma direta ou indireta, recebimento por palestras, aulas, atuação como proctor de treinamentos, remunerações, honorários pagos por participações em conselhos consultivos, de investigadores, ou outros comitês, etc. Provenientes da indústria farmacêutica, de órteses, próteses, equipamentos e implantes, brasileiras ou estrangeiras: - Amgen: Repatha; Amryt Pharma: Lojuxta; AstraZeneca: Dapagliflozina; Aché: Trezor, Trezete; Biolab: Livalo; Abbott: Lipidil; EMS: Rosuvastatina; Eurofarma: Rosuvastatina; Sanofi: Praluent, Zympass, Zympass Eze, Efluelda; Libbs: Plenance, Plenance Eze; Novo Nordisk: Ozempic, Victoza; Servier: Acertamlo, Alertalix; PTCBio: Waylivra. B - Financiamento de pesquisas sob sua responsabilidade direta/pessoal (direcionado ao departamento ou instituição) provenientes da indústria farmacêutica, de órteses, próteses, equipamentos e implantes, brasileiras ou estrangeiras. - PTCBio: Waylivra; Amgen: Repatha; Novartis: Inclisiran, Pelacarsen; NovoNordisk: Ziltivekimab. Outros relacionamentos Financiamento de atividades de educação médica continuada, incluindo viagens, hospedagens e inscrições para congressos e cursos, provenientes da indústria farmacêutica, de órteses, próteses, equipamentos e implantes, brasileiras ou estrangeiras: - Novo Nordisk: Diabetes.
Maria Elizabeth N. Caetano Costa	Declaração financeira A - Pagamento de qualquer espécie e desde que economicamente apreciáveis, feitos a (i) você, (ii) ao seu cônjuge/companheiro ou a qualquer outro membro que resida com você, (iii) a qualquer pessoa jurídica em que qualquer destes seja controlador, sócio, acionista ou participante, de forma direta ou indireta, recebimento por palestras, aulas, atuação como proctor de treinamentos, remunerações, honorários pagos por participações em conselhos consultivos, de investigadores, ou outros comitês, etc. Provenientes da indústria farmacêutica, de órteses, próteses, equipamentos e implantes, brasileiras ou estrangeiras: - Libbs: Plenance Enze; Servier: Vastarel. Outros relacionamentos Financiamento de atividades de educação médica continuada, incluindo viagens, hospedagens e inscrições para congressos e cursos, provenientes da indústria farmacêutica, de órteses, próteses, equipamentos e implantes, brasileiras ou estrangeiras: - Libbs; Servier: Participação em congresso.
Maria Sanali Moura de Oliveira Paiva	Nada a ser declarado
Maria Sotera Paniagua de Decoud	Nada a ser declarado
Marildes Luiza de Castro	Declaração financeira A - Pagamento de qualquer espécie e desde que economicamente apreciáveis, feitos a (i) você, (ii) ao seu cônjuge/companheiro ou a qualquer outro membro que resida com você, (iii) a qualquer pessoa jurídica em que qualquer destes seja controlador, sócio, acionista ou participante, de forma direta ou indireta, recebimento por palestras, aulas, atuação como proctor de treinamentos, remunerações, honorários pagos por participações em conselhos consultivos, de investigadores, ou outros comitês, etc. Provenientes da indústria farmacêutica, de órteses, próteses, equipamentos e implantes, brasileiras ou estrangeiras: - Novartis: Sacubitril/Valsartana; Pfizer: Patisiran; Merck: Vericiquat; Amgen.
Martha Beatriz Sanchez-Zambrano	Nada a ser declarado
Milena dos Santos Barros Campos	Nada a ser declarado
Monica Acevedo	Declaração financeira A - Pagamento de qualquer espécie e desde que economicamente apreciáveis, feitos a (i) você, (ii) ao seu cônjuge/ companheiro ou a qualquer outro membro que resida com você, (iii) a qualquer pessoa jurídica em que qualquer destes seja controlador, sócio, acionista ou participante, de forma direta ou indireta, recebimento por palestras, aulas, atuação como proctor de treinamentos, remunerações, honorários pagos por participações em conselhos consultivos, de investigadores, ou outros comitês, etc. Provenientes da indústria farmacêutica, de órteses, próteses, equipamentos e implantes, brasileiras ou estrangeiras: - Recebi honorários por consultoria, palestras e pesquisas da Abbott, AstraZeneca, Axon-pharma, Bayer, Boehringer Ingelheim, Eli Lily, Eurofarma, Ferrer, Novartis, Novo Nordisk, Tecnofarma, Teva Pharmaceuticals. Todos custaram menos de 5.000 dólares. Outros relacionamentos Financiamento de atividades de educação médica continuada, incluindo viagens, hospedagens e inscrições para congressos e cursos, provenientes da indústria farmacêutica, de órteses, próteses, equipamentos e implantes, brasileiras ou estrangeiras: - AstraZeneca: Congresso da Sociedade Europeia de Cardiologia, Barcelona 2022; Boehringer: Congresso da Sociedade Europeia de Cardiologia, Amsterdã 2023.
Monica Susana Ramirez	Outros relacionamentos Financiamento de atividades de educação médica continuada, incluindo viagens, hospedagens e inscrições para congressos e cursos, provenientes da indústria farmacêutica, de órteses, próteses, equipamentos e implantes, brasileiras ou estrangeiras: - Gador, Bago, Raffo. Participação societária de qualquer natureza e qualquer valor economicamente apreciável de empresas na área de saúde, de ensino ou em empresas concorrentes ou fornecedoras da SBC: - Centro de Diagnostico Rosario.
Olga Ferreira de Souza	Nada a ser declarado
Orlando Otávio de Medeiros	Nada a ser declarado
Regina Coeli Marques de Carvalho	Nada a ser declarado
Rogério Bonassi Machado	Nada a ser declarado
Sheyla Cristina Tonheiro Ferro da Silva	Declaração financeira A - Pagamento de qualquer espécie e desde que economicamente apreciáveis, feitos a (i) você, (ii) ao seu cônjuge/ companheiro ou a qualquer outro membro que resida com você, (iii) a qualquer pessoa jurídica em que qualquer destes seja controlador, sócio, acionista ou participante, de forma direta ou indireta, recebimento por palestras, aulas, atuação como proctor de treinamentos, remunerações, honorários pagos por participações em conselhos consultivos, de investigadores, ou outros comitês, etc. Provenientes da indústria farmacêutica, de órteses, próteses, equipamentos e implantes, brasileiras ou estrangeiras: - Palestras para Novartis: Entresto; AstraZeneca: Forxiga/ Xigduo; aliaça Boeringher-Lilly: Jardiance; Servier: Acertil, Acertalix, triplixan; Novonordisk: Saxenda, Ozempic, Rybelsus; Libbis: Naprix; Vifor: Ferinject. Outros relacionamentos Financiamento de atividades de educação médica continuada, incluindo viagens, hospedagens e inscrições para congressos e cursos, provenientes da indústria farmacêutica, de órteses, próteses, equipamentos e implantes, brasileiras ou estrangeiras: - Aliança Boeringher-Lilly: Jardiance; Novonordisk: Ozempic, Rybelsus, Saxenda; Servier: Acertil, Acertalix, Triplixan.
Thais de Carvalho Vieira Rodrigues	Nada a ser declarado
Viviana de Mello Guzzo Lemke	Nada a ser declarado
Walkiria Samuel Avila	Nada a ser declarado

## 1. Introdução

Após a publicação dos resultados de ensaio clínico realizado pelo *Women's Health Initiative* (WHI) em 2002, demonstrando que há mais riscos do que benefícios para a saúde feminina com a utilização de estrogênio (isolado ou combinado a progestagênio) para o controle dos sinais e sintomas da menopausa,^1^ houve uma diminuição progressiva e sustentada na prescrição desses medicamentos.^2-4^

Nos Estados Unidos, havia ocorrido um aumento de prescrições da terapia hormonal da menopausa (THM) de 16 milhões em 1966 para 90 milhões em 1999,^5-7^ de forma que, no final dos anos 1990, em torno de 25% das mulheres com 45-74 anos de idade^8^ e mais de 40% daquelas com 50-69 anos de idade encontravam-se em uso dessa terapia.^5-7^

Há evidências de que, após a publicação do estudo realizado pelo WHI,^1^ ocorreu uma queda nas prescrições de THM de 25% para 11,9% em 2003-2004, atingindo 4,7% em 2012, em todos os grupos demográficos estudados.^9^ Assinala-se que, mesmo após novas evidências demonstrando que a THM poderia ser utilizada em mulheres mais jovens que não apresentam riscos adicionais e se encontram dentro dos primeiros dez anos da menopausa, não houve ascensão na prescrição, que hoje se encontra em 4-6% das mulheres nessa fase.^10^

Estima-se na atualidade uma população global de 8.019.876.189 de pessoas, sendo 49,75% do sexo feminino e com uma expectativa de vida ao nascer de 76 anos (6 anos a mais que os homens),^11^ com mais acesso à educação e ao mercado de trabalho (apesar da indiscutível e persistente desigualdade de gênero observada) e que tende a sofrer com os sinais e sintomas da menopausa pelo menos durante um terço da vida. E, à medida que essas mulheres envelhecem, apresentam um risco cada vez mais acentuado de morbimortalidade cardiovascular (CV),^12,13^ considerando que um terço da mortalidade atual em mulheres é decorrente da doença isquêmica do coração (DIC) e da doença cerebrovascular.^14^

Segundo Faubion e Shufelt,^10^ as novas gerações de mulheres chegarão na menopausa com maior liberdade e segurança para falar mais abertamente sobre o sofrimento imposto pelos sinais e sintomas dessa etapa da vida, com mais propensão para a busca de soluções e a possibilidade de movimentar um mercado de produtos estimado em 600 bilhões de dólares. Esse grande contingente de mulheres precisa, assim, contar com um sistema de atendimento em saúde preparado para esse cenário. Ainda segundo essas autoras,^10^ para enfrentar esse desafio, é necessário o avanço na ciência da menopausa no que diz respeito à investigação científica, à formação e atualização dos profissionais que lidam com a saúde da mulher (medicina interna, endocrinologia, cardiologia, medicina da família, além, portanto, da ginecologia e obstetrícia), à criação de políticas públicas de estado para a educação em saúde e para o cuidado das mulheres, bem como à educação dos empregadores e dos líderes nas organizações de trabalho, que precisam adaptar esses locais para atender às necessidades das mulheres nessa etapa da vida.

Nesse contexto, o delineamento, a organização e a apresentação desta "Diretriz sobre a Saúde Cardiovascular no Climatério e Menopausa", resultante de um trabalho conjunto de sociedades científicas nacionais [Federação Brasileira das Associações de Ginecologia e Obstetrícia (FEBRASGO), Associação Brasileira de Climatério (SOBRAC) e Sociedade Brasileira de Cardiologia (SBC)] e internacional [*Sociedad Interamericana de Cardiología* (SIAC)], assim como de várias especialidades que lidam no cotidiano com a saúde da mulher, preenchem um dos requisitos importantes para a educação e/ou atualização dos profissionais que trabalham nesse campo, consistindo na divulgação das melhores evidências científicas disponíveis na atualidade sobre o tema climatério e menopausa.^15^

**Figura 1.1:**
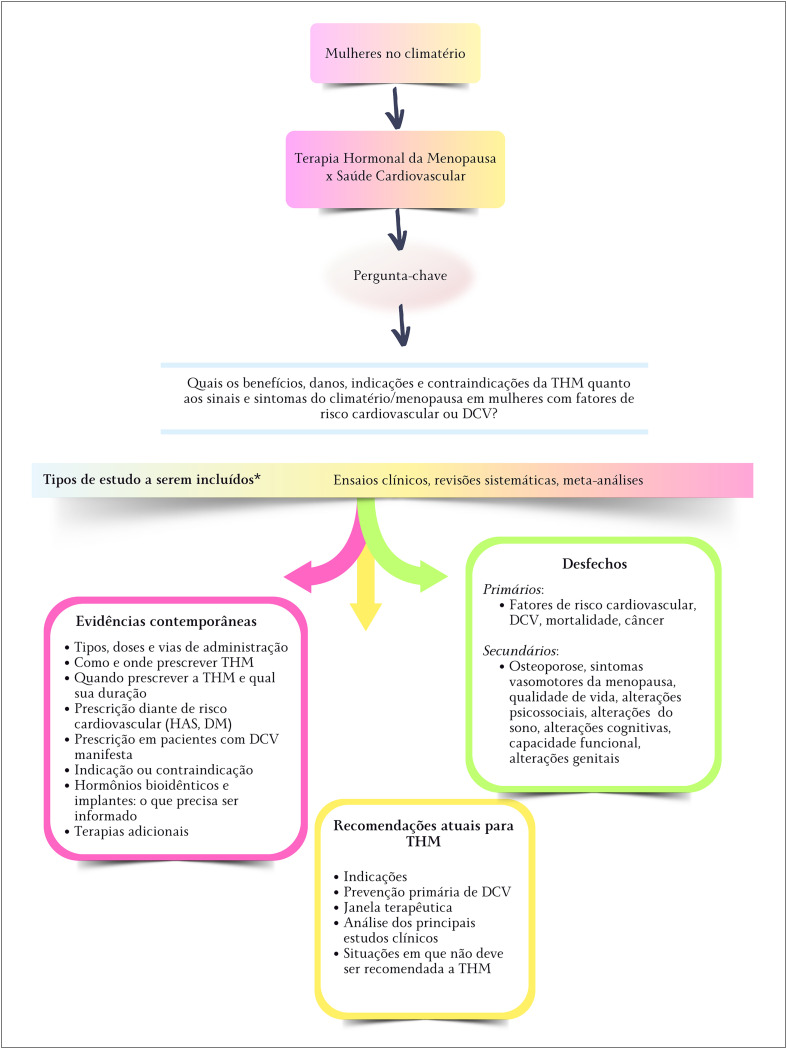
Estrutura da Revisão Sistemática que norteou a Diretriz de Climatério e Menopausa. DCV: doença cardiovascular; DM: diabetes mellitus; HAS: hipertensão arterial sistêmica; THM: terapia hormonal da menopausa.

Para a realização deste documento, procedeu-se a uma revisão sistemática (Anexo 1), registrada no PROSPERO 2024 CRD42024504299 *Available from*: https://www.crd.york.ac.uk/prospero/display_record.php?ID=CRD42024504299. Os métodos empregados para sua realização estão descritos no apêndice dessa diretriz (Figura 1.1).

A seguir descrevemos os destaques de cada capítulo.

## 1.1. Highlights

### Hormônios Sexuais (Estrogênio, Progesterona, Testosterona) e suas Funções ao Longo da Vida

A esteroidogênese ovariana inicia-se na puberdade, quando os hormônios atuam nos caracteres sexuais secundários e na regulação da gestação. Os hormônios sexuais (estrogênios, androgênios e progesterona), através de seus receptores presentes em quase todo o organismo feminino, exercem ações e funções específicas.

A síndrome do climatério é o conjunto de sintomas e sinais resultantes da interação entre fatores socioculturais, psicológicos e endócrinos. Seu diagnóstico em mulheres acima de 45 anos, na presença de queixas sugestivas de hipoestrogenismo, não requer confirmação por outros exames complementares.

A deficiência do estradiol na menopausa contribui para a disfunção endotelial pela perda de suas funções vasculares, tais como aumento da síntese de óxido nítrico, ação antioxidante e propriedades anti-inflamatórias.

Na transição menopáusica, iniciam-se alterações no perfil lipídico, como aumento no colesterol total, LDL colesterol e triglicerídeos.

O hipoestrogenismo leva a alteração no armazenamento e na distribuição de gordura corporal feminina, com aumento da adiposidade central (forma androide) e do risco cardiovascular.

### Relação entre Climatério/Menopausa e Fatores de Risco Cardiovascular Tradicionais e/ou Emergentes

A redução da função protetora do HDL colesterol e o aumento da concentração de Lp (a) na perimenopausa contribuem para o aumento do risco cardiovascular. Alterações do metabolismo glicídico associadas ao aumento da adiposidade central predispõem ao desenvolvimento de diabetes mellitus, que, na presença de menopausa precoce, leva ao maior aumento do risco cardiovascular.

O risco de doença isquêmica do coração aumenta na menopausa, além de piorar o prognóstico das portadoras da doença previamente, com maiores taxas de revascularização e evolução para insuficiência cardíaca.

Mulheres com hipertensão arterial sistêmica têm maior incidência de hipertrofia ventricular esquerda na pós-menopausa, com maior risco de disfunção diastólica. A hipertensão sistólica isolada nessa fase está relacionada à maior rigidez aórtica.

O sedentarismo na pós-menopausa leva a pior condicionamento físico e menor controle dos fatores de risco cardiovascular, além de maior incidência de fraturas e de mortalidade. O tabagismo aumenta o risco de menopausa precoce e a probabilidade de doença cardiovascular, acidente vascular cerebral, osteoporose, diabetes mellitus e mortalidade por todas as causas.

Na transição menopáusica, há maior risco de depressão e ansiedade. Gatilhos emocionais associados ao estresse crônico levam à ativação sustentada do eixo hipotálamo-hipófise-adrenal, com desregulação do perfil metabólico e inflamação sistêmica, acelerando a aterosclerose e aumentando o risco cardiovascular.

### Relação entre o Climatério/Menopausa e Doenças Cardiovasculares

A estratificação do risco cardiovascular desde o climatério é importante ferramenta para identificar os fatores e marcadores de risco e implementar medidas para prevenção e redução da mortalidade nas mulheres. Na ausência de escores específicos na perimenopausa e pós-menopausa, utilizam-se os escores tradicionais, podendo ser refinados pela identificação de fatores potencializadores de risco e marcadores de aterosclerose subclínica.

Mulheres têm carga global de aterosclerose menor e maior disfunção microvascular em coronárias. A menopausa precoce está associada ao aumento da mortalidade por doença isquêmica do coração.

As mulheres idosas, de etnia negra e menor nível socioeconômico têm maior incidência de acidente vascular cerebral, sendo a hipertensão arterial o principal fator de risco associado. Hipertensão arterial, diabetes mellitus e tabagismo têm maior impacto negativo nas mulheres, que também apresentam mais desfechos negativos e taxa de mortalidade aumentada após acidente vascular cerebral.

Na pós-menopausa, são mais frequentes a insuficiência cardíaca sistólica e diastólica e o remodelamento concêntrico do ventrículo esquerdo, sendo suas incidências aumentadas na menopausa precoce.

Múltiplos fatores sugerem correlação entre menopausa e risco aumentado de fibrilação atrial, como hipertensão arterial sistólica, obesidade, sedentarismo, ingesta excessiva de álcool, doença valvar, multiparidade e acidente vascular cerebral. Sugere-se que menopausa precoce aumente o risco de fibrilação atrial, assim como estresse, ansiedade, insônia e sintomas depressivos.

### Menopausa e Risco de Morbidade e Mortalidade por Outras Doenças

Existe risco cardiovascular aumentado em mulheres na pós-menopausa tratadas de câncer de mama, exacerbado pelo controle inadequado dos fatores de risco e pela cardiotoxicidade do tratamento.

Em mulheres com câncer, pode haver menopausa precoce, dependendo da reserva ovariana basal, da gonadotoxicidade e da duração da exposição aos agentes cancerígenos (terapia oncológica e/ou terapia endócrina).

O avanço da idade, o perfil genético e a presença de doença vascular sistêmica são os principais fatores de risco não modificáveis para o desenvolvimento de demência. Sua prevalência é maior entre as mulheres.

As disfunções tireoidianas são significativamente mais comuns em mulheres e sua incidência aumenta com o envelhecimento. O hipertireoidismo manifesto e o subclínico aumentam o risco de osteoporose, especialmente na pós-menopausa.

A perda do estrogênio na menopausa leva a remodelamento ósseo negativo e perda óssea, aumentando o risco de osteoporose. A terapia hormonal da menopausa deve ser indicada para mulheres com insuficiência ovariana prematura e na menopausa natural, podendo ser indicada para prevenção de osteoporose, especialmente na presença de sintomas vasomotores associados.

### Risco Cardiovascular e Hormônios Sexuais

Na estratificação de risco cardiovascular, é necessário incluir a avaliação de antecedentes ginecológicos e o uso de hormônios sexuais ao longo da vida.

Menarca precoce, síndrome dos ovários policísticos e uso de contraceptivos hormonais devem ser reconhecidos como fatores de risco cardiovascular adicionais.

A contracepção hormonal oral combinada exerce efeito protetor no sistema cardiovascular. Em ciclos anovulatórios por hipoestrogenismo e disfunção hipotalâmica, porém, aumenta o risco de aterosclerose coronariana e eventos cardiovasculares.

A suplementação da testosterona em mulheres não deve ser indicada para melhora do risco cardiovascular.

Independentemente dos possíveis efeitos adicionais no risco cardiovascular da terapia hormonal para afirmação de gênero, devemos manter o foco da prevenção nos pilares clássicos da saúde cardiovascular.

### Recomendações Atuais para Terapia Hormonal da Menopausa

- A Sociedade Brasileira de Cardiologia, a Federação Brasileira das Associações de Ginecologia e Obstetrícia e a Associação Brasileira de Climatério recomendam a **FAVOR** da adoção da terapia hormonal da menopausa para as mulheres climatéricas sintomáticas sem contraindicações. (**Força da recomendação a FAVOR. Recomendação FORTE. Nível de certeza ALTO**).
- Essa terapia consiste na administração de diferentes hormônios sexuais que devem ser individualizados de acordo com os riscos e benefícios de cada mulher. As várias formulações, doses e vias de administração de terapia hormonal têm alta eficácia no alívio de sintomas do climatério. (**Força da recomendação a FAVOR. Recomendação FORTE. Nível de certeza ALTO**).
- A terapia hormonal da menopausa deve ser iniciada na "janela de oportunidade", isso é, nos primeiros 10 anos após o início da menopausa e/ou antes dos 60 anos de idade.* Contrariamente, iniciar a terapia hormonal da menopausa com mais de 60 anos de idade ou mais de 10 anos após a menopausa pode elevar o risco absoluto de doença coronariana, tromboembolismo venoso e acidente vascular cerebral. (**Força da recomendação a FAVOR. Recomendação FORTE. Nível de certeza ALTO**).
- Não há indicação de iniciar a terapia hormonal da menopausa com o objetivo de prevenção primária cardiovascular nos múltiplos cenários. (**Força da recomendação a FAVOR. Recomendação FORTE. Nível de certeza ALTO**).

### Evidências Contemporâneas da Terapia Hormonal em Mulheres

Mulheres menopausadas com fatores de risco para doença cardiovascular necessitam de uma avaliação criteriosa antes de iniciar a terapia hormonal da menopausa.

Mulheres com hipertensão arterial sistêmica controlada e sintomas vasomotores moderados a intensos podem utilizar terapia hormonal da menopausa por qualquer via, mas deve-se preferir a terapia estrogênica transdérmica na presença de obesidade, dislipidemia, diabetes mellitus e síndrome metabólica. Recomenda-se a utilização da progesterona micronizada (via oral ou vaginal) nas mulheres não histerectomizadas.

Não se recomenda terapia hormonal da menopausa sistêmica em mulheres com doença cardiovascular manifesta, histórico prévio de infarto agudo do miocárdio ou acidente vascular cerebral. A terapia hormonal da menopausa transdérmica é recomendada para aquelas com histórico prévio de tromboembolismo venoso, a depender do fator que ocasionou o evento.

Para mulheres com contraindicação ou que não desejam usar terapia hormonal da menopausa, terapias não hormonais podem ajudar no alívio dos sintomas vasomotores.

Hormônios bioidênticos manipulados ou implantes hormonais não são recomendados pela falta de evidência científica de eficácia e segurança desses compostos.

### Menopausa e a Mulher No Mercado de Trabalho – Dificuldades e Oportunidades de Melhorias

As mulheres constituem grande parte da força de trabalho global, sendo que quase metade delas se encontra na idade da peri- ou pós-menopausa.

Os sintomas da menopausa prejudicam a qualidade de vida, o desempenho e a assiduidade no trabalho. Portanto, os empregadores precisam estar atentos ao desconforto ocasionado por esses sintomas, propiciando um ambiente de trabalho humanizado e confortável.

É preciso criar políticas institucionais de apoio às trabalhadoras que se encontram na menopausa (educação sobre o tema, consultas médicas quando necessário, adaptações no ambiente de trabalho).

É preciso promover abertura para a abordagem sobre o tema junto às lideranças, em busca de soluções para os problemas apresentados.

Medidas, como horários flexíveis, áreas mais ventiladas e mais próximas a banheiros, uniformes mais leves e confortáveis, são eficazes e custo-efetivas. Essas medidas devem ser priorizadas nas políticas dos empregadores para mulheres que se encontram na peri- ou pós-menopausa e se mantêm ativas profissionalmente.

### Menopausa e Climatério na América Latina – Situação Atual, Desafios e Oportunidades de Intervenção

Em países de baixa e média renda, há um aumento na prevalência de insuficiência ovariana prematura, ou seja, antes dos 40 anos de idade, e menopausa precoce, antes dos 45 anos, ambas consideradas fatores de risco para a prevalência de doença e mortalidade cardiovascular.

A média para o início da menopausa na América Latina é de 47,24 anos, com elevação progressiva na prevalência da insuficiência ovariana prematura e menopausa precoce.

Os sintomas vasomotores estão entre os de maior prevalência (55%) em mulheres latino-americanas em transição menopáusica e costumam ser graves em uma parcela importante dessa população.

Além dos sintomas vasomotores, distúrbios do sono, distúrbios urogenitais, dores músculo-articulares e alterações do humor (depressão, ansiedade, irritabilidade) são frequentes e comprometem a qualidade de vida de mulheres em transição menopáusica e na pós-menopausa.

A prescrição de terapia hormonal da menopausa na América Latina ocorre em 12,5% das mulheres menopáusicas (via oral, 43,7%; transdérmica, 17,7%; terapias alternativas, 19,5%).

## 2. Hormônios Sexuais (Estrogênio, Progesterona, Testosterona) e suas Funções ao Longo da Vida

Na vida intrauterina, entre a sexta e a oitava semana de gestação, em embrião do sexo cromossômico feminino, 46XX, ocorre a diferenciação da gônada embrionária, bipotencial, em ovários. Na ausência do cromossomo Y, o feto desenvolve ovários e, na ausência de níveis de testosterona semelhantes aos masculinos, surge o fenótipo feminino.^16^ Os ovários iniciam sua esteroidogênese na puberdade e seus hormônios, fundamentalmente o estradiol e a progesterona, são responsáveis pelo desenvolvimento dos caracteres sexuais secundários e pela regulação da gestação.^17^

Na verdade, endocrinologicamente, o primeiro sinal para a puberdade é dado pelas suprarrenais (adrenarca). Com a maturação e o crescimento da zona reticular adrenal, ocorre um aumento dos androgênios adrenais, dehidroepiandrosterona (DHEA) e sulfato de dehidroepiandrosterona (DHEAS), que resultará em aumento da testosterona. Esse aumento da testosterona é responsável pela maturação das glândulas sudoríparas apócrinas, levando ao odor tipo adulto, desenvolvimento de acne e pelos pubianos e axilares. Portanto, os pelos pubianos se desenvolvem independentemente da ativação do sistema hipotálamo-hipófise-gonadal.^18,19^

A partir da puberdade, com ativação do eixo hipotálamo-hipófise, os ovários irão secretar estrogênios, em especial o estradiol, pelas células da granulosa dos folículos, sendo que, para a sua síntese, é necessária a produção de androgênios, especialmente a testosterona, pelas células da teca. Sabe-se que, nos primeiros 1,5-2 anos de atividade ovariana, os ciclos são anovulatórios e, portanto, desprovidos da produção de progesterona. O estradiol estimula o desenvolvimento das mamas (telarca), o crescimento do esqueleto e o desenvolvimento dos órgãos genitais internos (útero, tubas uterinas e segmento superior da vagina) e externos (vulva e terço inferior da vagina), que culminam com o início das menstruações (menarca). Quando os ciclos ovarianos se tornam ovulatórios, o corpo lúteo resultante da ovulação passa a secretar progesterona juntamente com estradiol. A progesterona é responsável pelas mudanças, sobretudo do endométrio, necessárias para manutenção da gestação.^18^

Existem receptores para os hormônios sexuais (estrogênios, androgênios e progesterona) em praticamente todos os tecidos e órgãos do corpo da mulher. Dessa forma, eles atuam e apresentam funções específicas em todo o organismo feminino.

Há muito se sabe que os estrogênios desempenham um papel crucial na coordenação de muitos eventos neuroendócrinos que controlam o desenvolvimento sexual, o comportamento sexual e a reprodução. O estradiol é fundamental para a diferenciação sexual do cérebro. Na verdade, ele organiza circuitos neurais e regula a apoptose de neurônios, levando a diferenças de longo prazo no cérebro feminino. Além de seu papel no desenvolvimento, o estradiol previne a morte de células neuronais em uma variedade de modelos de lesão cerebral, modula o aprendizado e a memória, promove a formação de sinapses e influencia na síntese de neurotransmissores, bem como na apoptose celular. A testosterona, agindo no cérebro, parece regular a reprodução, sexualidade e comportamentos emocionais em ambos os sexos em um contexto diferente relacionado ao gênero. A progesterona por sua ação no sistema nervoso central possui efeito hipnótico/sedativo, ansiolítico e anestésico/analgésico.^18,20,21^

O estradiol exerce um efeito cardioprotetor positivo através de sua influência na função endotelial, miocárdica, vascular e metabólica. Vasos coronarianos e periféricos contêm receptores de estrogênio que permitem que o estradiol desempenhe um papel regulador na vascularização. O estrogênio estimula a síntese de óxido nítrico (NO) através de efeitos genômicos e não genômicos, causando vasodilatação. Os hormônios sexuais influenciam os mecanismos envolvidos na regulação da pressão arterial (PA), sendo que os estrogênios, mas não os androgênios, exercem um efeito favorável sobre os níveis da PA a longo prazo, sobretudo por mecanismos relacionados aos rins.^22,23^

Os estrogênios influenciam os efeitos vasculares do LDL colesterol (LDL-c). Estradiol, que é um fenol com propriedades antioxidantes, previne a oxidação de LDL-c e VLDL colesterol (VLDL-c) e protege a vasculatura contra os efeitos deletérios dos lipídios. Ele atenua o acúmulo de LDL-c minimamente modificado e LDL-c oxidado na parede arterial e previne oxidação e acumulação, mediada por fator de necrose tumoral α, de LDL-c na parede arterial. Além disso, aumenta a expressão de receptor de LDL-c, aumenta a depuração de VLDL-c, diminui a produção de LDL-c, diminui o tamanho das partículas de LDL-c, aumenta a depuração de LDL-c leve e densa, entre outros.^24^

O processo de remodelação óssea, que mantém um esqueleto saudável, pode ser considerado um programa de manutenção preventiva, removendo continuamente os ossos mais velhos e substituindo-os com osso novo. Os estrogênios são essenciais na promoção do equilíbrio entre os eventos da remodelação óssea, reabsorção e formação.^25^

Portanto, desde a puberdade e durante toda a fase reprodutiva da mulher (menacme), os hormônios sexuais exercem efeitos específicos e fundamentais não somente no sistema reprodutor, mas em todos os órgãos e sistemas do corpo feminino. Sempre considerar os estrogênios, especialmente o estradiol, como o ator principal, a progesterona essencial na manutenção da gestação e a testosterona como coadjuvante em algumas funções específicas.

### 2.1. Alterações Hormonais na Menopausa

As mulheres nascem com todo o seu conjunto de folículos, cerca de 1-2 milhões. No início da puberdade, a massa de células germinativas já foi reduzida para 300-500 mil unidades. Durante os próximos 35-40 anos de vida reprodutiva, 400-500 serão selecionados para ovular e os folículos primários acabarão por se esgotar até próximo à menopausa, quando restarão apenas algumas centenas.^16,17^

Durante o período reprodutivo, os oócitos (folículos) são gradualmente esgotados através da ovulação e da atresia (apoptose – morte celular programada). A diminuição do número de oócitos resulta na menor secreção de inibina B, diminuindo o *feedback* negativo ovariano sobre o hormônio folículo-estimulante (FSH). O aumento resultante no nível de FSH leva a um maior recrutamento folicular e a uma perda folicular acelerada, com preservação dos níveis de estradiol na transição precoce da menopausa. Quando as mulheres estão na faixa dos 40 anos, a anovulação torna-se mais prevalente em face da qualidade e da capacidade reduzidas dos folículos envelhecidos e, como consequência, ocorre a ausência de produção de progesterona. Quando todos os folículos ovarianos estão esgotados, o ovário é incapaz de responder mesmo a níveis elevados de FSH e os níveis de estrogênio diminuem. O período pós-menopausa é caracterizado por um FSH elevado (> 30 mUI/mL) e níveis baixos de estradiol (< 30 pg/mL).^17^

O ovário pós-menopáusico secreta principalmente androstenediona e testosterona. Após a menopausa, o nível circulante de androstenediona é cerca de metade do observado antes da menopausa. A maior parte dessa androstenediona pós-menopáusica é derivada da glândula adrenal, com apenas uma pequena quantidade secretada pelo ovário, embora a androstenediona seja o principal esteroide secretado pelo ovário pós-menopáusico. A produção de testosterona diminui aproximadamente 25% após a menopausa, mas o ovário pós-menopáusico na maioria das mulheres, mas não em todas, secreta mais testosterona do que o ovário na pré-menopausa.^17,26^

O nível circulante de estradiol após a menopausa é de aproximadamente 10-20 pg/mL, a maior parte do qual é derivada da conversão periférica de estrona, que, por sua vez, é derivada principalmente da conversão periférica de androstenediona. O nível circulante de estrona em mulheres na pós-menopausa é maior que o de estradiol, aproximadamente 30-70 pg/mL. A taxa média de produção de estrogênio na pós-menopausa é de aproximadamente 45 μg/24 horas, sendo quase todos, se não todos, estrogênios derivados da conversão periférica da androstenediona. A proporção androgênio/estrogênio muda drasticamente após a menopausa devido ao declínio mais acentuado do estrogênio, sendo comum o aparecimento de hirsutismo leve, refletindo essa mudança acentuada na proporção dos hormônios sexuais.^17,26^

### 2.2. Definição e Classificação

A "menopausa natural" é definida como a data do último episódio de sangramento menstrual de uma mulher.^27^ Ocorre em média aos 51 anos e 90% das mulheres passam por esse período entre 45 anos e 55 anos de idade.^28^ A menopausa espontânea que ocorre entre 40 anos e 45 anos atinge cerca de 5% das mulheres e é denominada "*early menopause*" em inglês.^29^ A "menopausa induzida" é a interrupção da menstruação que ocorre após ooforectomia bilateral cirúrgica ou perda da função ovariana iatrogênica decorrente de quimioterapia (Qt) ou radioterapia.^29^ A insuficiência ovariana prematura (IOP) é uma síndrome resultante da perda da atividade ovariana antes dos 40 anos de idade,^30^ afetando aproximadamente 1% das mulheres.^31^ O termo "menopausa prematura" pode ser usado para se referir aos casos definitivos de menopausa antes dos 40 anos, como os decorrentes de ooforectomia bilateral.^29^ O termo "transição menopáusica" (TM) refere-se ao período da vida em que ocorrem alterações do ciclo menstrual em decorrência da diminuição da função ovariana, começando com a variação na duração do ciclo menstrual e terminando com o último episódio de sangramento menstrual.^29^

**Figura 1.2:**
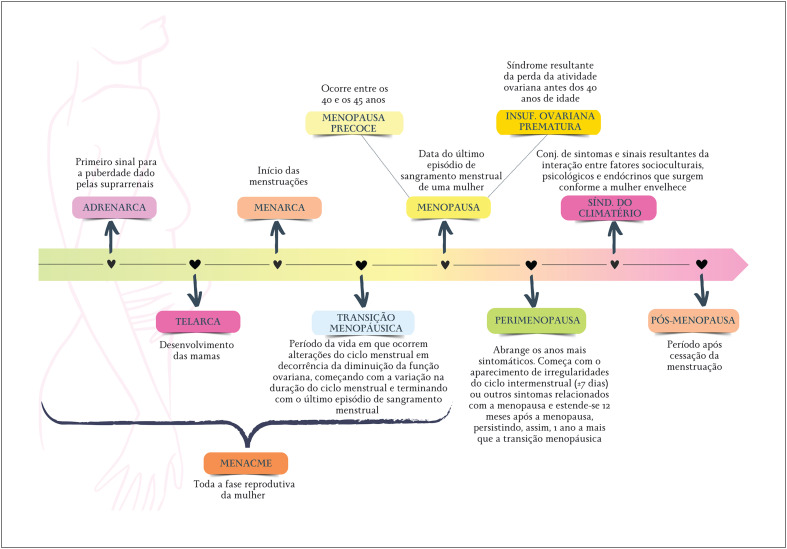
Nomenclaturas relacionadas aos ciclos de vida da mulher empregados nessa diretriz.

**Figura 2.1:**
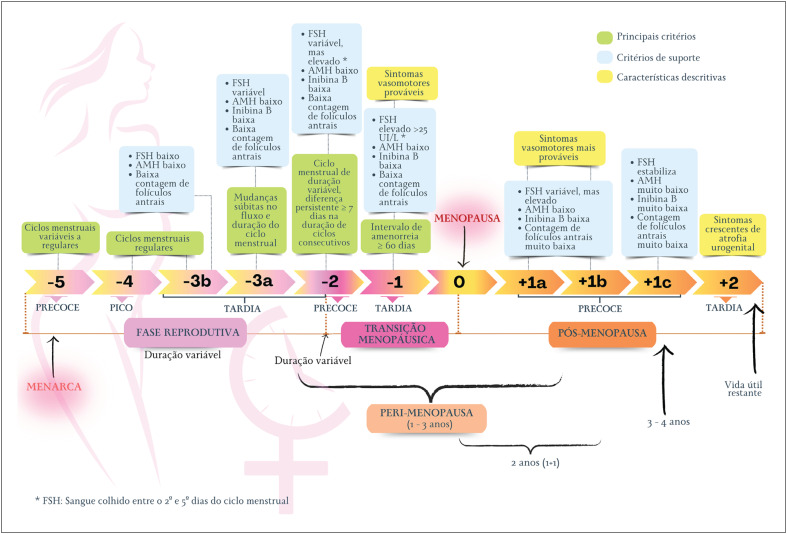
Sistema STRAW de classificação dos estágios reprodutivos. FSH: hormônio folículo-estimulante; AMH: hormônio antimülleriano.

A expressão "síndrome do climatério" engloba o conjunto de sintomas e sinais resultantes da interação entre fatores socioculturais, psicológicos e endócrinos que surgem conforme a mulher envelhece.^27^ A Figura 1.2 ilustra as nomenclaturas relacionadas aos ciclos de vida da mulher utilizados nessa Diretriz, desde a puberdade ao fim da vida reprodutiva. Para padronizar a definição dos diversos estágios do envelhecimento reprodutivo, foi criado o sistema STRAW (*Stages of Reproductive Aging Workshop* - Oficina sobre Estágios do Envelhecimento Reprodutivo, em tradução livre).^32^ Com base em padrões de sintomas e achados laboratoriais, o sistema STRAW classifica o envelhecimento reprodutivo nas seguintes fases: reprodutiva, TM e pós-menopausa. A Figura 2.1 ilustra os detalhes do sistema STRAW.^32^

### 2.3. Diagnóstico Clínico e Laboratorial

O processo de envelhecimento leva à falência ovariana progressiva, resultando na interrupção dos ciclos ovulatórios e no término do sangramento menstrual. Com frequência, mulheres buscam assistência devido a alterações no ciclo menstrual durante a TM. Devido à redução da produção de inibina B pelos ovários ao final da quarta década de vida, é possível observar um aumento nas concentrações séricas de FSH e estradiol no início do ciclo, resultando no encurtamento da fase folicular. Além disso, a qualidade do corpo lúteo piora, levando a uma diminuição nos níveis de progesterona na fase secretora. O encurtamento do intervalo entre as menstruações é um dos primeiros sinais da diminuição da função ovariana.^33^

À medida que os anos avançam, o processo de depleção folicular persiste e a anovulação torna-se cada vez mais comum. Devido à falta de contraposição progestacional, o intervalo entre os ciclos menstruais se estende, chegando a 40-50 dias. Esse aumento médio no intervalo entre os ciclos menstruais ocorre por volta dos 47 anos.^33^ Episódios mais prolongados de amenorreia começam a ocorrer, intercalados por episódios de sangramento menstrual de volume variável. Esse padrão de sangramento menstrual pode persistir por um período de um a três anos antes da menopausa.^33^

Os sintomas vasomotores (SVM), conhecidos também como fogachos ou ondas de calor, são os mais comumente vinculados à TM. Esses sintomas envolvem sensações abruptas de calor na região central do corpo, especialmente na face, tórax e pescoço, e têm uma duração média de 3-4 minutos.^34^ Frequentemente, esses episódios são acompanhados por um aumento na frequência cardíaca (FC), vasodilatação periférica, elevação da temperatura da pele e sudorese. Se ocorrerem durante a madrugada, podem estar associados a distúrbios do sono, como insônia.^35^ Os SVM moderados/severos ocorrem em até 80% das mulheres.^36^ No entanto, apenas cerca de 20-30% delas buscam assistência médica para tratamento.^33^ No início do declínio da função ovariana, os SVM podem ser leves, ocorrendo no nadir da secreção de estradiol, durante as fases lútea tardia e folicular inicial. A ocorrência de SVM aumenta significativamente durante a TM, atingindo aproximadamente 40% na transição precoce e elevando-se para 60-80% durante a transição tardia da menopausa e nos estágios iniciais da pós-menopausa.^37^ Na pós-menopausa tardia, a ocorrência dos SVM tende a diminuir; entretanto, até 30% das mulheres podem apresentar SVM moderados/severos após 10 anos da menopausa.^36^

A caracterização da data da menopausa é feita retrospectivamente após 12 meses de amenorreia em uma mulher na faixa etária esperada para a TM.^23^ O diagnóstico da síndrome do climatério é estabelecido por meio de uma anamnese detalhada, complementada por um exame físico minucioso.^38^ Para mulheres com mais de 45 anos que apresentam queixas sugestivas de hipoestrogenismo, como SVM e alterações típicas do padrão menstrual (sangramento uterino pouco frequente), o diagnóstico da síndrome do climatério é clínico e não requer confirmação por outros exames complementares.^38^ Em casos em que há dúvidas quanto à sintomatologia decorrente da queda na produção ovariana de estradiol, a dosagem de FSH na fase folicular inicial pode ser útil para confirmar o diagnóstico. Valores acima de 25 mUI/mL podem indicar o início da TM. No entanto, é importante notar que as concentrações diárias podem variar de maneira considerável nessa fase. Recomenda-se, quando necessário, realizar duas dosagens com um intervalo de 4-6 semanas entre elas.^38^ Vale ressaltar que a maioria das mulheres em contracepção hormonal à base de progestagênios isolados terá padrões de sangramento alterados ou amenorreia, dificultando a orientação precisa sobre o *status* menopáusico. Se necessário, mulheres em contracepção hormonal com progestagênios isolados podem realizar medições séricas de FSH para avaliar o *status* menopáusico.^39^ Níveis > 25 mUI/mL são atribuíveis ao declínio da função ovariana. No entanto, os progestagênios isolados, como o acetato de medroxiprogesterona de depósito e os implantes hormonais, podem suprimir o FSH, o que significa que uma mulher em uso dessas medicações pode estar na perimenopausa sem mostrar aumento nos níveis de FSH.^39^ O momento ideal para medir os níveis de FSH em uma mulher usando acetato de medroxiprogesterona de depósito é logo antes de uma nova administração da medicação.^40^ Mulheres usando contraceptivos hormonais combinados têm níveis significativamente suprimidos de FSH, mesmo durante a fase livre de hormônios, o que os torna inadequados para informar aconselhamento sobre o *status* da menopausa. Além disso, os SVM são menos frequentes em decorrência dos efeitos do componente estrogênico do contraceptivo.^39^ Em mulheres usuárias de contraceptivos combinados em que seja necessário dosar o FSH, orienta-se suspender a medicação de 2-4 semanas antes da coleta sanguínea.^33^

Padrões de sangramento que não se encaixam nos previstos para o declínio da função ovariana, como sangramento muito frequente, em volume aumentado, com coágulos, demandam a investigação do endométrio com ultrassonografia e/ou biópsia endometrial.^38^ Para mulheres com idade inferior a 45 anos que apresentem queixas de sangramento uterino anormal, com padrão irregular e ciclos menstruais pouco frequentes, mesmo que o quadro clínico sugira hipoestrogenismo, recomenda-se uma investigação adicional para avaliar os sintomas e excluir outras causas de irregularidade menstrual, como gravidez, distúrbios tireoidianos e hiperprolactinemia.^35^

### 2.4. Relação com a Mortalidade Cardiovascular

A doença arterial coronariana (DAC) é a mais comum causa de morte em mulheres na pós-menopausa, maior do que casos de câncer de mama ou outro câncer ginecológico. Os fatores de risco (FR) tradicionais para DAC incluem idade, tabagismo, estilo de vida sedentário, má alimentação, índice de massa corporal (IMC) elevado, hipertensão arterial sistêmica (HAS), diabetes mellitus (DM), dislipidemia (DLP) e história familiar de DAC. Mulheres na pré-menopausa têm baixa prevalência de DAC, provavelmente devido aos efeitos protetores dos estrogênios em mulheres.^41^ Há um aumento acentuado na incidência de DAC em mulheres após a menopausa, normalmente encontrado cerca de 10 anos após o último período menstrual.^42^

É pouco provável que a menopausa por si só conduza a essa mudança, sendo que outros FR, como DLP, resistência à insulina, redistribuição de gordura e HAS, podem causar alterações metabólicas e vasculares contribuindo para risco acelerado de DAC e doenças cardiovasculares (DCV). Estas situações clínicas podem estar relacionadas a efeitos periféricos adversos da função endotelial.

O envelhecimento vascular é caracterizado por enrijecimento progressivo das artérias com declínio na capacidade de vasodilatação, que progride de forma diferente em homens e mulheres. No início da menopausa, ocorre de forma acelerada, diferentemente da perda gradual da função vascular observada com o avançar da idade. A disfunção endotelial e o envelhecimento vascular contribuem para o desenvolvimento de HAS e aterosclerose, favorecendo o aumento das DCV na menopausa.^43,44^

O estradiol é crucial para a manutenção da função endotelial normal, aumentando a síntese de NO pelo endotélio vascular, processo conhecido como vasodilatação dependente do endotélio, cuja perda é uma característica marcante da disfunção endotelial e que é rapidamente afetada pelo declínio dos hormônios ovarianos com o envelhecimento reprodutivo na menopausa.^45^

Estudos demonstraram que o estradiol apresenta propriedades antioxidantes e anti-inflamatórias. A deficiência de estrogênio regula positivamente o estresse oxidativo ou inflamação sistêmica, levando à diminuição da função endotelial.^46^ Assim, o estrogênio tem múltiplas funções, como aumento da síntese de NO, antioxidante e propriedades anti-inflamatórias. Sua deficiência na menopausa contribui para a disfunção endotelial.^47^

As alterações no perfil lipídico das mulheres começam ainda no período da TM, com aumentos no colesterol total (CT), LDL-c, triglicerídeos (TG). O *Women's Health Across the Nation* (SWAN) foi um estudo prospectivo da TM em caucasianas e representantes das minorias (afro-americanas, hispânicas, japonesas, chinesas) e que não estavam em terapia hormonal. Esse estudo forneceu evidências de que a TM está ligada a perfis lipídicos adversos. Foi demonstrado que TG, LDL-c e apolipoproteína-B aumentam já no intervalo de 1 ano próximo ao último período menstrual, independentemente da idade na qual isso ocorre. Todos esses fatores estão diretamente ligados à disfunção endotelial e levam à aterosclerose. Um aumento no LDL-c na TM está ligado ao aparecimento de placas carotídeas na pós-menopausa.^48-50^ Essas mudanças são distintas das mudanças lineares relacionadas ao envelhecimento cronológico.

A síndrome metabólica (SM) é definida como a coexistência de vários FR metabólicos como HAS, DLP, intolerância à glicose e adiposidade central. O estradiol desempenha um papel importante no armazenamento e na distribuição de gordura. Antes da menopausa, a gordura é principalmente depositada em coxas, nádegas e quadris. As mulheres tendem a ganhar peso (gordura corporal total) durante a meia-idade em função da cronologia do envelhecimento. No entanto, quando as mulheres passam pela TM, há uma mudança na composição corporal bem como na distribuição de gordura, com aumento da adiposidade central.^51^ A TM pode, assim, contribuir para o aumento da gordura abdominal, resistência à insulina, DM e doenças inflamatórias, levando ao desenvolvimento ou agravamento da SM em mulheres.^51-53^

A evolução do processo aterosclerótico parece ser o resultado final de uma interação complexa entre DCV, FR e sua acentuação durante o período da perimenopausa. O aumento do risco cardiovascular (RCV) na menopausa decorre de importantes mudanças na fisiologia do sistema CV que afetam a vasculatura periférica, cardíaca e sistemas cerebrovasculares. Mudanças no perfil lipídico, rigidez vascular, parâmetros metabólicos e estresse oxidativo contribuem para o agravamento do RCV em mulheres na TM.

**Figura 2.2:**
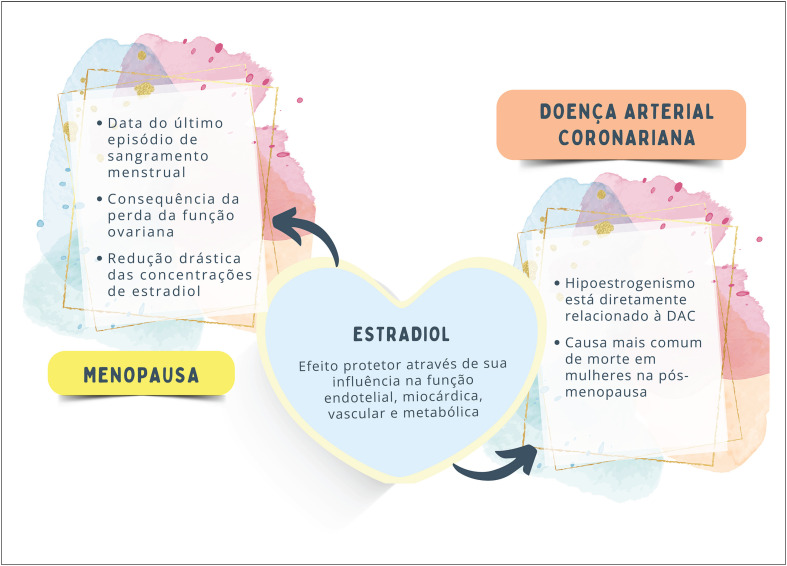
Hipoestrogenismo e doença arterial coronariana (DAC).

As estratégias de tratamento devem incluir controle rigoroso dos fatores de risco cardiovascular (FRCV) para prevenir o avanço da doença aterosclerótica em mulheres na menopausa. A Figura 2.2 sintetiza as interações entre hipoestrogenismo e DAC.

## 3. Relação entre Climatério/Menopausa e Fatores de Risco Cardiovascular Tradicionais e/ou Emergentes

### 3.1. Introdução

Até o ano de 2025, haverá mais de 1,1 bilhão de mulheres na pós-menopausa em todo o mundo, representando 12% de toda a população mundial. Com o envelhecimento populacional, as mulheres poderão passar cerca de metade de suas vidas nessa fase. A TM é um marco na vida da mulher, associada a sintomas incômodos, como ondas de calor, suores noturnos, problemas de sono e distúrbios de humor, que comprometem a qualidade de vida. A menopausa também está associada a doenças crônicas, como DCV, DM, neoplasias e osteoporose.^54^

O aumento das DCV na perimenopausa seria devido ao envelhecimento cronológico ou ao envelhecimento ovariano? Dados recentes de estudos longitudinais mostraram que fatores relacionados à menopausa, como idade mais precoce da menopausa e a menopausa cirúrgica, estão relacionados a mais desfechos CV. A perimenopausa também está associada aos FR cardiometabólicos, incluindo composição corporal, acúmulo de gordura visceral, HAS, DLP, SM, estresse crônico, sedentarismo, tabagismo e os determinantes sociais de saúde.^54,55^

A TM é um momento de aceleração do risco de DCV, sendo importante a monitoração da saúde das mulheres durante a meia-idade, por representar uma janela crítica para implementação de estratégias de intervenção precoce para reduzir o risco de DCV. Desse modo, torna-se muito importante a discussão dos FRCV associados à TM, à perimenopausa e à pós-menopausa.^55^

### 3.2. Hipertensão

A HAS é o FRCV mais prevalente e modificável e o que está associado às maiores taxas de morte e DALYs (anos de vida ajustados por incapacidade; do inglês, *Disability-Adjusted Life Years*) no mundo e no Brasil, em ambos os sexos. A prevalência de HAS aumenta com a idade em ambos os sexos, mas esse aumento é acentuado em mulheres após a menopausa e acima dos 65 anos, superando a dos homens da mesma faixa etária.^56^ A HAS que ocorre nessa fase do ciclo de vida das mulheres parece ser mais sensível à carga de sal e está mais frequentemente associada à SM e ao aparecimento de efeitos adversos de medicamentos, em comparação à HAS nos homens com a mesma idade.^57^

De acordo com dados do Vigitel para 2021, em relação à HAS autorreferida, a mais elevada prevalência de HAS no país, 61% (intervalo de confiança - IC 95%, 59,0-63,0), foi observada em indivíduos com idade a partir de 65 anos, sendo que, nesse grupo etário, as mulheres apresentaram maior prevalência do que os homens, 63,7% (IC 95%, 61,6-65,8) e 57,1% (IC 95%, 53,4-60,7), respectivamente.^58^ Nessa faixa etária, menos da metade das mulheres na pós-menopausa têm a HAS controlada.^58^

As mulheres com HAS na pós-menopausa têm maior incidência de hipertrofia ventricular esquerda e maior risco de desenvolver disfunção diastólica em comparação com as mulheres adultas jovens. A HAS sistólica isolada em mulheres na pós-menopausa está relacionada à maior rigidez aórtica, provavelmente causada por proliferação de células de músculo liso, acúmulo de colágeno e aumento dos níveis de moléculas vasoconstritoras na parede dos vasos sanguíneos devido à falta do efeito protetor do estrogênio.^59,60^

A falta do estradiol pode interferir negativamente na vasodilatação devido aos efeitos sobre o sistema renina-angiotensina-aldosterona (SRAA), o sistema do NO, a endotelina e o sistema imunológico. A falta de estradiol também pode afetar a biodisponibilidade do NO, devido à atividade reduzida da superóxido dismutase, e a resposta imune humoral e celular.^59,60^ Por outro lado, o declínio nos níveis de progesterona pode estar, pelo menos em parte, associado com a ocorrência de HAS nas mulheres na pós-menopausa, dado que a progesterona atua como um hormônio vasoativo, prevenindo a ação de vasoconstrição induzida pela noradrenalina, agindo diretamente nas células da musculatura lisa vascular. Além disso, em mulheres na pós-menopausa, baixos níveis de DHEAS, andrógeno e precursor de hormônios esteroides, foram associados com maior mortalidade CV e por todas as causas.^61^ Cabe ressaltar que foram descritas duas mudanças cruciais na regulação autonômica durante a menopausa que são capazes de facilitar o desenvolvimento de HAS: aumento do fluxo simpático central e aumento da sensibilidade adrenérgica nos vasos sanguíneos periféricos.^55^

Embora o estrogênio tenha um papel protetor em mulheres na pré-menopausa, a administração de estrogênios exógenos a mulheres na menopausa não tem efeito na PA e não afeta o risco de desfechos CV. Após o início da THM, será necessário monitorar a PA e, caso não se observe controle adequado, a THM deve ser interrompida.^59^

A absorção, a distribuição, o metabolismo e a excreção de medicamentos anti-hipertensivos são diferentes entre mulheres e homens provavelmente devido à influência dos hormônios sexuais na absorção (P-glicoproteína), ao volume de distribuição, à atividade de citocromo P450 (CYPs) e à depuração renal.^62^ Os efeitos adversos dos medicamentos anti-hipertensivos são relatados com mais frequência em mulheres, em especial na menopausa, como a tosse induzida por inibidores da enzima de conversão da angiotensina, o edema de tornozelo com bloqueadores dos canais de cálcio, a hipocalemia e a hiponatremia com diuréticos. Esses efeitos adversos podem explicar a menor adesão das mulheres, na menopausa, ao tratamento da HAS.^57-60,62^

### 3.3. Sobrepeso/Obesidade

As alterações fisiológicas e metabólicas associadas à menopausa são um efeito direto da deficiência de estrogênio, que afeta o metabolismo lipídico, o consumo de energia, a resistência insulínica e a composição de gordura corporal, com uma transição de um formato corporal ginecoide para um androide, com aumento do acúmulo de gordura abdominal e visceral, diagnosticados pela medida da circunferência da cintura e da relação cintura-quadril. Essas alterações foram associadas ao aumento dos riscos metabólico e CV e daqueles relacionados ao diabetes tipo 2 (DM2), ao LDL-c e ao câncer de endométrio e de mama.^63^

Resultados de estudos de coorte de longa duração com um grande número de mulheres, como o SWAN e o WHI, sugerem que o aumento da obesidade no climatério, medido pelo IMC, seja uma consequência da idade e que esse aumento ocorre tanto em mulheres previamente obesas quanto em não obesas, após a menopausa. Observou-se ausência ou modesta associação entre obesidade e início tardio da menopausa.^64,65^ Mulheres na pós-menopausa com obesidade têm um risco 4 vezes maior de mortalidade CV.^64,65^ Os estudos longitudinais SWAN e WHI mostraram que existem diferenças étnicas nas mudanças físicas e metabólicas que ocorrem durante o climatério.^64,65^

Estudos demonstraram que mulheres na perimenopausa com obesidade apresentam SVM menos intensos do que mulheres com peso normal, possivelmente devido a níveis mais baixos de estradiol e FSH, pela aromatização de andrógenos em estrogênios no tecido adiposo, que retroalimenta negativamente o hipotálamo e glândulas pituitárias, diminuindo o FSH e a secreção ovariana de estrogênio. Outros sintomas presentes em mulheres obesas após a menopausa, particularmente aqueles associados com o aumento da circunferência abdominal, são a apneia e outros distúrbios do sono e sintomas geniturinários.^66,67^

Mulheres com obesidade têm maior probabilidade de serem sintomáticas na perimenopausa e necessitarem de THM. Porém, seu uso está associado com maior risco de tromboembolismo venoso (TEV), complicações CV e câncer de mama e do endométrio, especialmente em obesas. Desse modo, impõe-se rigorosa avaliação do risco-benefício dessa terapêutica, mesmo quando indicada. Nesse caso, sugere-se o uso de adesivos com progesterona micronizada e baixa dose de estrogênio, empregados por curto período.^63,68^ Estudos demonstraram que mudanças no estilo de vida previnem a adiposidade visceral associada com a perimenopausa e melhoram os sintomas e os riscos cardiometabólicos adversos.^63,67^

### 3.4. Síndrome Metabólica

Estudos transversais demonstraram que, em comparação com mulheres na pré-menopausa, mulheres na pós-menopausa têm significativamente mais obesidade visceral e SM. Meta-análise realizada com artigos publicados entre 2004 e 2017 (119 estudos, n = 95.115) demonstrou prevalência de SM na pós-menopausa de 37,17% (IC 95%, 35,00%-39,31%). A *odds ratio* (OR) agrupada para SM em mulheres na pós-menopausa em comparação com mulheres na pré-menopausa (23 estudos, n = 66.801) foi 3,54 (IC 95%, 2,92-4,30). As chances de glicemia de jejum elevada (OR 3,51; IC 95% 2,11-5,83), HDL colesterol (HDL-c) baixo (OR 1,45; IC 95%, 1,03-2,03), PA elevada (OR 3,95; IC 95%, 2,01-7,78), TG elevados (OR 3,2; IC 95%, 2,37-4,31) e circunferência da cintura aumentada (OR 2,75; IC 95%, 1,80-4,21) foram todas mais elevadas em mulheres na pós-menopausa do que em mulheres na pré-menopausa.^69^

Mulheres na pré-menopausa tendem a ter maiores depósitos periféricos de gordura acumulando-se na região gluteofemoral ("em forma de pera"). Contudo, durante o período da da menopausa e pós-menopausa, a deposição de gordura tende a acumular-se centralmente. Este dado, associado à perda do efeito protetor do endotélio pela privação estrogênica, contribui para a disfunção endotelial, estado inflamatório e rigidez arterial, resultando em aumento do risco de DCV. Além disso, as mulheres na pós-menopausa têm tendência a atingir níveis mais elevados de CT, LDL-c, TG, lipoproteína (a) [Lp(a)] e níveis mais baixos de HDL-c em comparação com mulheres na perimenopausa, o que representa uma mudança para um perfil lipídico pró-aterogênico e pró-coagulante fortemente ligado ao aumento da gordura visceral e demais FR classicamente importantes para DCV.^70^

Estudo utilizando os dados de 1.470 mulheres da *Atherosclerosis Risk in Communities cohort* (ARIC), com acompanhamento de 10 anos através de 4 visitas, reportou aumentos graduais na gravidade da SM ao longo do tempo, sendo que as mulheres negras exibiram progressão mais rápida na gravidade da SM durante os períodos de TM e perimenopausa do que durante o período pós-menopausa, no qual foram observadas alterações favoráveis na taxa de variação da circunferência da cintura, TG, HDL-c e glicose. Esses dados sugerem que a maior prevalência de SM em mulheres na pós-menopausa pode ser causada mais por alterações durante a TM do que na pós-menopausa, sugerindo maior RCV da SM no período da perimenopausa.^71^

A presença e a gravidade da SM parecem estar associadas a um risco aumentado de DM2 no período perimenopausa. Por outro lado, a menopausa cirúrgica está fortemente associada à maior incidência de SM. Curiosamente, as mulheres com síndrome dos ovários policísticos (SOP) têm um risco aumentado de SM durante os anos reprodutivos e, durante a TM, o risco de SM torna-se semelhante ao de mulheres sem SOP.^72^

### 3.5. Sedentarismo

O sedentarismo é um dos FRCV e marcador de prognóstico independente de mortalidade.^73,74^ Foi demonstrado que as mulheres sedentárias exibiam, na pós-menopausa, piores condicionamento físico e controle dos demais FRCV quando comparadas às que praticavam exercícios físicos.^75^ No último posicionamento da *American Heart Association* (AHA) sobre o constructo da saúde CV, foi introduzido o oitavo elemento: o comportamento do sono.^76^ Os estudos demonstraram associação entre piora da qualidade do sono e sedentarismo na pós-menopausa^77^

Os resultados do estudo WHI evidenciaram que mulheres na menopausa com tempo sedentário maior que 9,5 horas/dia, tiveram risco significativamente maior de 24% de hospitalização incidente por insuficiência cardíaca.^78^ Uma das justificativas é o aumento da atividade do sistema nervoso simpático e do SRAA.^79^

A cessação da função ovariana após a menopausa ocasiona declínio significativo do estrogênio, causando aceleração da perda óssea e osteoporose em 20-30% das mulheres, aumentando a probabilidade de fraturas e a mortalidade. Os exercícios físicos melhoram a força muscular e o equilíbrio para prevenir quedas, restauram a autoconfiança e a coordenação e, adicionalmente, mantém a massa óssea, estimulando a formação óssea e diminuindo a reabsorção.^80^ Os exercícios físicos são recomendados também na prevenção do câncer de mama.^81^

As mulheres, em todos os períodos da vida, devem evitar comportamento sedentário para melhorar a qualidade de vida e reduzir as complicações do sedentarismo para a saúde.^82^

De acordo com as diretrizes da Organização Mundial da Saúde (OMS), adultos de meia-idade ativos devem realizar pelo menos 150 minutos/semana de exercícios aeróbicos de intensidade moderada, ou pelo menos 75 minutos de intensidade vigorosa, e associar exercícios físicos resistidos no mínimo duas vezes por semana, envolvendo maiores grupos musculares.^83^

### 3.6. Tabagismo

O tabagismo é considerado importante FR para as DCV. Os estudos demonstraram associação com a idade precoce da menopausa. As mulheres fumantes apresentaram o dobro do risco de desenvolver menopausa precoce e as ex-fumantes tiveram risco de 15% maior de IOP e menopausa precoce. Existe relação positiva com a intensidade, duração, dose cumulativa e início precoce do tabagismo.^84^

A idade precoce da menopausa está associada ao aumento da probabilidade de DCV, acidente vascular cerebral (AVC), osteoporose, DM e mortalidade por todas as causas. As mulheres fumantes morrem 11 anos antes das que nunca fumaram e têm maior prevalência de DCV e mortalidade por DCV e por todas as causas, reforçando a necessidade do abandono do vício.^64^

### 3.7. Estresse Crônico

O estresse crônico prejudica a saúde CV. As mulheres parecem ter comportamento mais extenuante às adversidades das relações sociais com trabalho, família e cônjuge.^85^ Alguns mecanismos são envolvidos na fisiopatologia para a DCV, como a ativação sustentada do eixo hipotálamo-hipófise-adrenal, a desregulação dos processos metabólicos e a inflamação sistêmica, que contribuem para o aumento da PA e no processo de aterosclerose.

As mulheres com relato de estresse crônico durante a meia-idade apresentaram espessura médio-intimal (EMI) da carótida significativamente maior do que as mulheres que nunca o relataram.^85^ A depressão foi associada à elevação do escore de cálcio coronariano (CAC) em mulheres na pós-menopausa,^86^ sendo considerada FR independente para morte por DCV e todas as causas.^87^ As mulheres apresentam maior risco de depressão e ansiedade durante a TM.^88^

O fator "estresse mental crônico" foi significativamente associado ao aumento do número de plaquetas CD63+ e à bioatividade plaquetária pró-inflamatória, sendo uma das possíveis explicações para a ligação entre transtornos mentais e somáticos na menopausa.^89^ A diminuição dos níveis de estrogênio em mulheres na pós-menopausa aumenta a suscetibilidade à cardiomiopatia de Takotsubo.^90^

Intervenções no estilo de vida, como alimentação saudável, exercícios físicos, tempo e qualidade do sono adequados e práticas de meditação e ioga para reduzir o estresse psicológico crônico na menopausa, reforçam a interligação entre as saúdes mental e CV.^64^

### 3.8. Dislipidemia

A menopausa resulta em vários distúrbios lipídicos devido às alterações hormonais, como diminuição dos níveis de estrogênio e aumento dos níveis de andrógenos circulantes. As alterações no metabolismo lipídico e o excesso de tecido adiposo desempenham um papel fundamental na síntese do excesso de ácidos graxos, adipocitocinas, citocinas pró-inflamatórias e espécies reativas de oxigênio, que causam peroxidação lipídica e resultam no desenvolvimento de resistência à insulina, adiposidade abdominal e DLP.^91^ O risco populacional atribuível à DLP é maior nas mulheres quando comparado a todos os demais FRCV. Entretanto os benefícios da redução dos níveis de LDL-c na regressão da aterosclerose têm a mesma magnitude nas mulheres e nos homens.^92^

Há também uma relação bidirecional dos FRCV e eventos CV com a ocorrência de menopausa precoce. Dados do *Framingham Heart Study* mostraram que o aumento do CT e da PA, além de outros FRCV, antes da menopausa foi associado à menopausa precoce, independentemente do tabagismo.^93^ Além disso, em uma análise agrupada de 177.131 mulheres de 9 estudos, um primeiro evento de DCV antes dos 35 anos de idade foi associado a uma duplicação do risco de menopausa precoce.^94^

Particularmente relevante é a relação entre HDL-c e menopausa. O estudo de SWAN^95^ sugere que a função antiaterogênica do HDL-c, ou seja, a capacidade de promover o transporte reverso de colesterol, pode diminuir durante a menopausa, estando associada a uma aparente inversão na direção da associação entre HDL-c e RCV, com níveis mais elevados de HDL-c associados a menos aterosclerose carotídea antes da menopausa, mas com maior aterosclerose carotídea após a menopausa.

Ainda no campo dos distúrbios lipídicos, a concentração de Lp(a) aumenta durante a gravidez e desde o início da menopausa (cerca de 50 anos). Além disso, níveis elevados de Lp(a) são mais comuns em mulheres do que em homens após os 50 anos, o que pode impactar o risco de DCV. Todas essas particularidades dos distúrbios lipídicos na menopausa sugerem que as atuais recomendações das diretrizes para DLP possam ser inadequadas para mulheres.^96^

### 3.9. Diabetes Mellitus

Além das alterações lipídicas, outros fatores metabólicos e clínicos secundários à menopausa, como resistência à insulina, redistribuição de gordura, disglicemia e DM, contribuem para o risco acelerado de envelhecimento e DCV. Durante a TM ocorrem várias alterações fenotípicas e metabólicas, afetando o peso corporal, a distribuição do tecido adiposo e o gasto energético, bem como a secreção e sensibilidade à insulina. Em conjunto, esses fatores podem predispor as mulheres ao desenvolvimento de DM.^97^

Mulheres com DM têm um risco 45% maior de desenvolver DIC, com risco de DAC fatal em mulheres com DM2 sendo 3 vezes maior do que nas não diabéticas, em especial na menopausa. A presença de DM resulta em menor indicação de revascularização e, consequentemente, maior ocorrência de insuficiência cardíaca em comparação aos homens, aumentando na menopausa.^92^

O DM, juntamente com a menopausa precoce, pode resultar em risco ainda maior de DCV em mulheres. O risco associado à menopausa precoce (< 45 anos) em comparação com a menopausa em idade normal foi estimado por Yoshida *et al*. durante seguimento de 15 anos.^98^ As taxas de risco ajustadas para evento CV na menopausa precoce foram maiores em mulheres com DM *versus* aquelas sem DM (DAC: 1,15 *versus* 1,09; AVC: 1,21 *versus* 1,10; doença aterosclerótica cardiovascular: 1,29 *versus* 1,10; insuficiência cardíaca: 1,18 *versus* 1,09).

Outro aspecto relevante é que embora seja mais prevalente nos homens, o DM2 confere maior aumento relativo, embora não necessariamente absoluto, do RCV em mulheres do que em homens, de todas as idades. Em parte, isso pode estar relacionado à maior adiposidade, já que as mulheres são tipicamente menos ativas fisicamente e têm um IMC mais elevado do que os homens, bem como a FR específicos do sexo para DM como, por exemplo, SOP e diabetes gestacional.^96^

**Figura 3.1:**
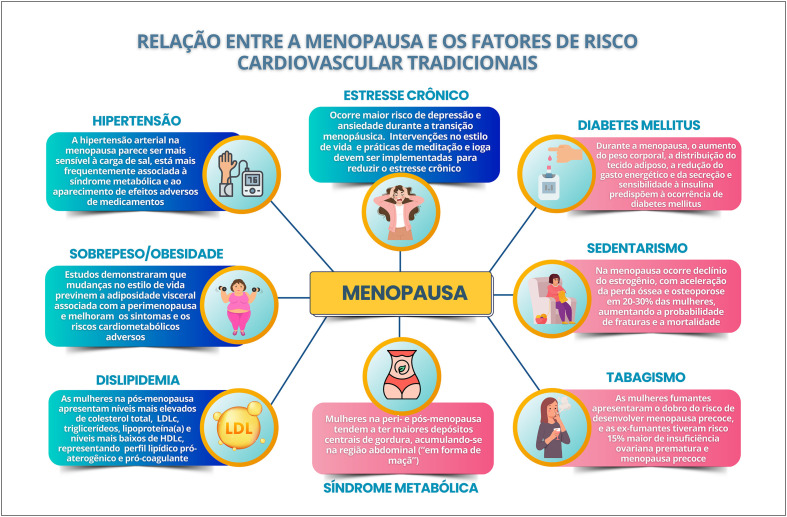
Relação entre menopausa e fatores de risco cardiovascular tradicionais.

A Figura 3.1 ilustra a relação entre a menopausa e os FRCV tradicionais.

### 3.10. Situação Econômica e Emprego

**Figura 3.2:**
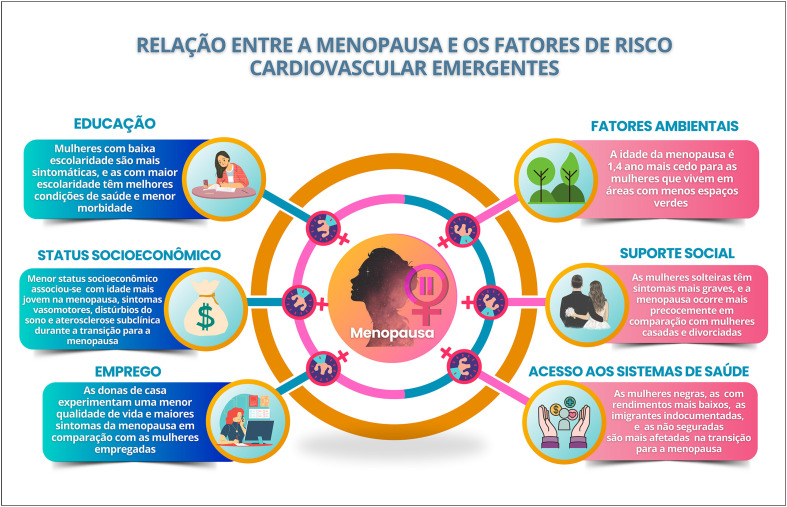
Relação entre a menopausa e os fatores de risco cardiovascular emergentes.

Os FR emergentes representam um desafio na DCV e o reconhecimento e a quantificação da associação com os desfechos CV são de difícil avaliação na atualidade. Sua modificação envolve não apenas ações individuais, mas coletivas e governamentais. Os riscos sociais associados ao envelhecimento refletem o enfraquecimento dos cuidados sociais e de saúde, que se tornam piores nos idosos quando sozinhos. Os determinantes sociais da saúde envolvem as condições sociais em que as pessoas nascem, vivem e trabalham e são críticos na morbidade e mortalidade CV (Figura 3.2).^98^

As donas de casa experimentam uma pior qualidade de vida e mais sintomas da menopausa em comparação às mulheres empregadas. Observou-se que a melhor condição financeira melhora a qualidade de vida na menopausa, possivelmente por maior acesso a serviços de saúde e aconselhamento sobre o controle dos sintomas da menopausa. Acredita-se que uma condição socioeconômica desfavorável leve à menopausa precoce e que essa ocorrência na infância também se associe a com aumento de tabagismo e menopausa precoce.^99,100^

Em 2020, quase 20% das mulheres de meia-idade nos Estados Unidos viviam na pobreza. O pior *status* socioeconômico foi relacionado à idade mais jovem na menopausa, SVM mais frequentes, distúrbios do sono e aterosclerose subclínica durante a TM.^101^

### 3.11. Baixa Educação em Saúde

De acordo com algumas pesquisas, há uma relação negativa entre o nível de conhecimento e a gravidade dos sintomas da menopausa. Mulheres com mais baixa escolaridade tendem a ser mais sintomáticas, enquanto aquelas com maior escolaridade apresentam melhores condições de saúde e menor morbidade. Ou seja, as mulheres com ensino superior estão mais conscientes dos sintomas da menopausa e das estratégias para seu enfrentamento, adotando um estilo de vida mais saudável, com menos disfunção sexual durante a menopausa, o que pode influenciar diretamente na satisfação sexual.^100,101^

Além disso, a idade média da menopausa em mulheres instruídas é maior do que a daquelas sem instrução. Adicionalmente, a educação do marido também afeta a qualidade de vida das mulheres na pós-menopausa, em especial na dimensão psicossocial, o que pode ser devido a uma melhor compreensão e apoio do cônjuge.^99,101^

### 3.12. Discriminação Racial

As mulheres negras tendem a entrar na menopausa em idade mais precoce do que as brancas não latinas e podem ter uma transição mais longa. Com relação à carga de sintomas, mulheres negras latinas e não latinas apresentam SVM mais frequentes, distúrbios do sono e depressão, enquanto as asiáticas não latinas são mais propensas a relatar diminuição da libido.^100,101^

### 3.13. Acesso aos Sistemas de Saúde

Numa amostra de mulheres predominantemente latinas que não têm abrigo e/ou não têm seguro de saúde, os investigadores descobriram que as mulheres sem abrigo relataram mais sintomas de menopausa em comparação àquelas com habitação. As mulheres negras, as que têm rendimentos mais baixos e as imigrantes indocumentadas são mais afetadas durante a TM. Foi demonstrado que mulheres não seguradas relatam sintomas de menopausa mais incômodos do que mulheres seguradas.^99,101^

### 3.14. Fatores Ambientais

Dados do Inquérito de Saúde Respiratória da Comunidade Europeia, uma coorte internacional de base populacional, mostrou que a idade da menopausa é 1,4 anos mais cedo nas mulheres que vivem em áreas com menos espaços verdes em comparação com as mulheres que vivem em áreas mais arborizadas.^102^

### 3.15. Suporte Social

As mulheres solteiras apresentam sintomas mais graves na menopausa e apresentam idade mais precoce da menopausa em comparação com mulheres casadas e divorciadas, com maior risco de osteoporose e DCV, provavelmente pelas relações sociais e apoio familiar. Ainda, as mulheres casadas têm melhor qualidade de vida na menopausa do que as solteiras e viúvas. Maior idade da última gravidez e maior número de gravidezes e partos retardam o início da menopausa, possivelmente devido ao aumento da secreção de estrogênio e progesterona pela atividade uterina e ovariana e à amamentação.^99,101^

### 3.16. Conclusão

A TM se traduz em diferentes experiências para as mulheres, influenciadas por crenças pessoais, normas culturais, comportamentos, assim como pelo ambiente social e pelos FRCV tradicionais. Os diversos fatores coexistem em vários níveis: individual, interpessoal, comunitário e coletivo, implicando no acesso desigual aos sistemas de saúde. Muitos desses fatores não foram contemplados nos ensaios clínicos, que precisam incluir mais mulheres durante os vários períodos da menopausa para que as estratégias diagnósticas e terapêuticas possam ser transpostas para essa fase da vida das mulheres.

## 4. Relação entre Climatério/Menopausa e Doenças Cardiovasculares

### 4.1. Cálculo do Risco Cardiovascular na Menopausa – Peculiaridades dos Estratificadores de Risco e Métodos de Imagem

Uma em cada três mulheres morre de DCV no mundo,^103^ risco que aumenta substancialmente após a menopausa.^104^

A mulher desenvolve DIC vários anos após o homem, com um notável aumento na TM.^105^ Entretanto, a estratificação do RCV em mulheres desde o climatério é uma importante ferramenta para identificar os principais fatores e marcadores de risco, visando primordialmente implementar estratégias e medidas terapêuticas na prevenção e redução da mortalidade. Como não existe um escore específico para estratificação de risco para mulheres na perimenopausa e pós-menopausa, os escores tradicionais são utilizados.

Os principais fatores que influenciam o RCV nas mulheres são a raça/etnia, história reprodutiva, como passado de diabetes gestacional e pré-eclâmpsia, saúde CV na pré-menopausa, atividade física, dieta, consumo de álcool, tabagismo e genética, além da idade da menopausa natural, tipo e estágios da menopausa, estrogênios endógenos, SVM, depressão e distúrbios do sono.^55^

Dados do *West Pomeranian Voivodeships*, usando os escores ASCVD, SCORE2 e POL-SCORE em mulheres em diferentes estágios da menopausa,^106^ mostraram que a maioria das participantes era de baixo RCV. Idade da menopausa, tempo desde a menopausa e presença de SM associaram-se a maior RCV (OR = 1,186; 1,267 e 13,812, respectivamente). Mulheres que apresentam menopausa antes dos 45 anos de idade têm maior mortalidade por DCV e mortalidade por todas as causas, porém mais estudos são necessários para definir se os desfechos CV negativos e a mortalidade relacionam-se ao tempo desde o início da menopausa ou aos mecanismos que levaram à menopausa precoce, como fatores genéticos, reprodutivos (paridade e idade da menarca) e relacionados ao estilo de vida (tabagismo, etilismo e IMC).

Nas mulheres, existem fatores potencializadores de risco (FPR),^107^ como doenças autoimunes (lúpus eritematoso sistêmico e artrite reumatoide), que aumentam de 2-3 vezes o RCV, além de outras menos comuns, como esclerose sistêmica, síndrome de Sjögren, polimialgia reumática, síndrome antifosfolípide e arterite de células gigantes. Importante ressaltar que o tratamento do câncer de mama com radioterapia e Qt com antraciclinas e trastuzumabe associa-se a maior risco de DCV, mesmo anos após o término do tratamento.

A estratificação de risco pode ser refinada com marcadores de aterosclerose subclínica, tais como CAC, índice tornozelo-braquial (ITB), EMI ou placa carotídea e angiotomografia coronariana (angio-TC) com presença de placa com obstrução < 50%, se persistir incerteza no manejo clínico com hipolipemiantes em prevenção primária após a inclusão de FPR.^107^

O estudo Multi-Ethnic Study of Atherosclerosis (MESA)^108^ demonstrou que ausência de calcificação coronária (CAC = 0 ocorreu em > 50% das mulheres) associou-se a risco de doença aterosclerótica CV baixo a moderado em 10 anos, sendo maior na menopausa precoce; CAC = 1-99 ou > 100 UA associou-se a maior incidência de doença aterosclerótica CV, sendo, porém, semelhante em mulheres com ou sem menopausa precoce.

**Figura 4.1:**
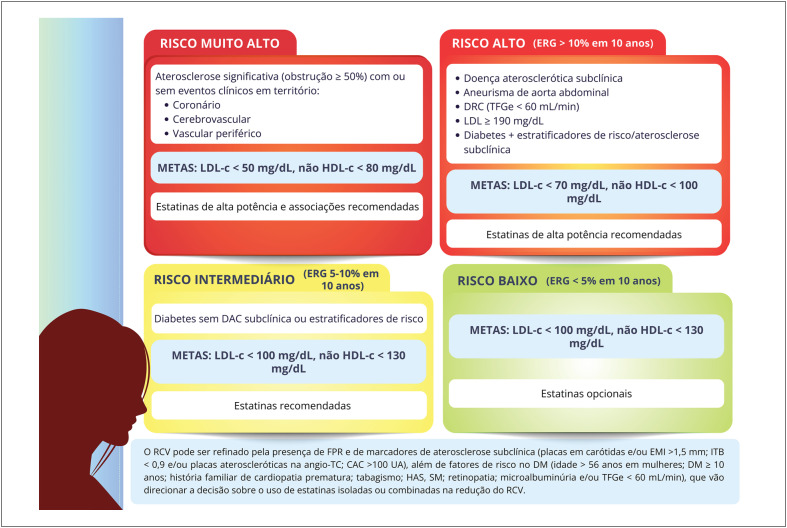
Estratificação do risco cardiovascular e metas terapêuticas em mulheres no climatério e na menopausa. Adaptado de Oliveira et al.^13^ angio-TC: angiotomografia coronariana; CAC: escore de cálcio coronariano; DAC: doença arterial coronariana; DM: diabetes mellitus; DRC: doença renal crônica; EMI: espessamento médio-intimal; ERG: escore de risco global; FPR: fatores potencializadores de risco; HAS: hipertensão arterial sistêmica; ITB: índice tornozelo-braquial; RCV: risco cardiovascular; SM: síndrome metabólica; TFGe: taxa de filtração glomerular estimada.

Assim, para definição terapêutica na estratificação do RCV em mulheres no climatério e menopausa (Figura 4.1), deve-se inicialmente considerar as situações de muito alto risco, como presença de doença aterosclerótica CV manifesta, e de alto risco (aterosclerose subclínica, aneurisma de aorta abdominal, doença renal crônica, diabetes com estratificação de risco e hipercolesterolemia severa). Nessas situações, estatinas de alta potência, isoladas ou combinadas, são fortemente recomendadas.

O risco intermediário e o baixo risco baseiam-se no escore de risco global (ERG). Nessas situações, o RCV pode ser refinado pela presença de FPR e de marcadores de aterosclerose subclínica (placas em carótidas e/ou EMI > 1,5 mm; ITB < 0,9 e/ou placas ateroscleróticas na angio-TC; CAC >100 UA), além dos FR no DM (idade > 56 anos em mulheres; DM ≥ 10 anos; história familiar de cardiopatia prematura; tabagismo; HAS, SM; retinopatia; microalbuminúria e/ou taxa de filtração glomerular estimada (TFGe) < 60 mL/min), que vão direcionar a decisão sobre o uso de estatinas isoladas ou combinadas na redução do RCV.^13^

Entretanto, estudos de desfechos em prevenção primária e secundária da doença aterosclerótica CV permanecem elusivos nas mulheres, sendo necessários para elaborar recomendações especificamente dirigidas às mulheres no climatério e na menopausa.^105^

### 4.2. Doença Isquêmica do Coração Aguda e Crônica

**Figura 4.2:**
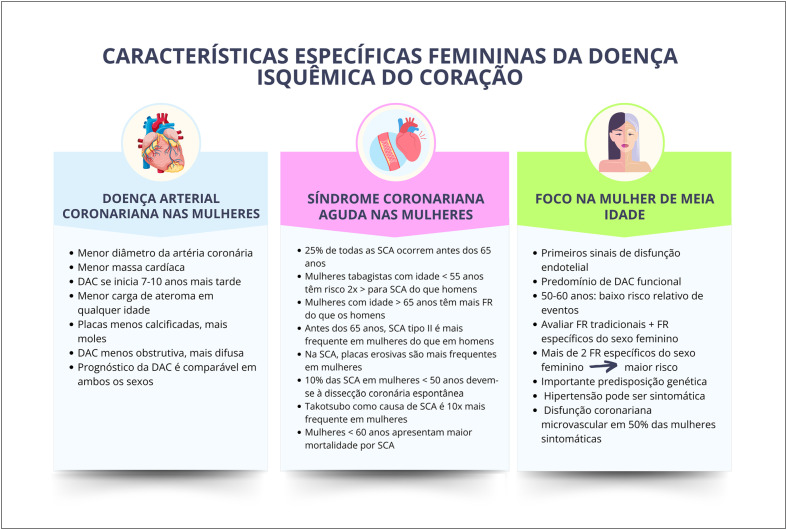
Características específicas da doença isquêmica do coração feminina em comparação à masculina. Adaptado de Elias-Smale et al.^121^ DAC: doença arterial coronariana; FR: fatores de risco; SCA: síndrome coronariana aguda.

Estudos recentes têm mostrado grandes avanços em relação à compreensão sobre a DIC em mulheres, que tem características específicas em relação aos sintomas e fisiopatologia, com impacto positivo nas taxas de mortalidade (Figura 4.2). Faz-se, porém, necessária uma investigação mais aprofundada dessa patologia no climatério e na menopausa.

O risco de eventos CV em mulheres mais jovens na pré-menopausa é menor; contudo, essa tendência se inverte com o envelhecimento. Mulheres na pré-menopausa apresentam um risco relativamente menor de DIC em comparação com homens da mesma faixa etária, mas essa discrepância de gênero diminui após a menopausa.^109^

#### 4.2.1. Alterações Anátomo-funcionais Coronarianas

A carga global de aterosclerose é menor nas mulheres, exibindo um padrão de DAC mais difuso e menos obstrutivo. No entanto, mais de 50% das mulheres sintomáticas na meia-idade demonstram disfunção microvascular coronariana.^109^

No climatério, as placas caracterizam-se por apresentar menor calcificação, o que está associado ao estrogênio, que exerce propriedades protetoras no processo de envelhecimento arterial. Contudo, observa-se um aumento gradual de placas mais vulneráveis e calcificadas após os 60 anos.^22^

No estudo Women's Ischemia Syndrome Evaluation (WISE), uma análise realizada em mulheres na pós-menopausa com suspeita de DIC revelou que aquelas com histórico de menstruações irregulares e evidências bioquímicas de hiperandrogenismo tiveram maior prevalência de DIC angiográfica e pior sobrevida em eventos CV. De maneira semelhante, o *Rancho Bernardo Study* concluiu que a DIC estava associada à SOP (histórico de menstruações irregulares, hiperandrogenismo, infertilidade, obesidade central e resistência à insulina) em uma extensa coorte de mulheres caucasianas na pós-menopausa.^110^

#### 4.2.2. Momento da Menopausa e Desenvolvimento de Doença Isquêmica do Coração Crônica

Estudos indicam que a menopausa precoce natural está associada a DCV, possivelmente pela perda dos efeitos vasoprotetores do estrogênio. Kalarantidou *et al*. demonstraram que mulheres com IOP apresentam função endotelial anormal, avaliada pela dilatação mediada por fluxo da artéria braquial, sendo possível alterar essa condição com terapia hormonal cíclica de estrogênio/progestagênio.^111^

Nos últimos anos, diversas pesquisas investigaram a relação entre a idade da menopausa natural e o risco de DIC, observando um aumento da mortalidade por DIC em mulheres com menopausa precoce, apesar de existirem especulações em relação ao risco aumentado em mulheres com menopausa muito tardia.^112^

Uma pesquisa envolvendo 302.632 mulheres chinesas revelou que a idade da menopausa e os anos totais de vida reprodutiva estavam inversamente associados a DCV fatais e não fatais, em especial DAC, com o aumento do risco com o decorrer do tempo desde a menopausa.^113^

#### 4.2.3. Doença Isquêmica do Coração Aguda

Na síndrome coronariana aguda (SCA), mulheres mais jovens apresentam uma probabilidade duas vezes menor de apresentar lesões significativas nas coronárias em comparação com os homens.^114^ Em homens e em mulheres a partir da TM, observa-se comumente o padrão clássico de ruptura de placa seguida pela formação de trombos. Entretanto, em mulheres mais jovens, a SCA frequentemente manifesta-se com erosões de placa e SCA tipo II (doença coronariana funcional).^115^

A dissecção coronária espontânea é mais prevalente em mulheres jovens, representando 10% de todas as SCA em idade inferior a 50 anos. Pode ocorrer em mulheres sem FR aparentes, associando-se a uma combinação de doença tecidual ou displasia fibromuscular. Além disso, está relacionada à pré-HAS durante a gravidez ou após o parto, sendo frequentemente desencadeada por situações de estresse.^116^

Quanto à apresentação clínica da SCA, sintomas indeterminados de dor torácica e dispneia são comuns em mulheres de meia-idade. A disfunção endotelial vascular emerge como a primeira manifestação do envelhecimento arterial, caracterizando-se por um desequilíbrio na vasodilatação e vasoconstrição, podendo resultar nas primeiras manifestações de dor torácica e dispneia.^117^ No estudo WISE, constatou-se que, em mais de 50% das mulheres de meia-idade, os sintomas de dor torácica estavam relacionados a disfunções vasculares em coronárias epicárdicas e microcirculação, e não à DAC obstrutiva.^118-120^

### 4.3. Doença Cerebrovascular

Entre as etiologias da doença cerebrovascular, o AVC é a mais prevalente, chegando a afetar 94 em cada 100 mil pessoas por ano em todo o mundo.^122,123^ Além disso, após um ataque isquêmico transitório ou um AVC isquêmico menor, 6,2% dos pacientes são afetados por novo AVC dentro de um ano, sendo que o risco de recorrência aumenta para uma taxa cumulativa estimada de 12,9% ao longo de cinco anos.^124^

A doença cerebrovascular é importante causa mundial de morbimortalidade e tem particularidades nas mulheres,^122^ representando a segunda causa de morte e a terceira causa de incapacidade. As mulheres têm maior incidência de AVC do que os homens nas idades mais avançadas, o que pode ser parcialmente explicado pelo maior tempo de vida delas.^122^ Foram ainda relatadas disparidades significativas por raça/etnia nessa faixa etária, sendo que mulheres negras e hispânicas com idade ≥ 70 anos apresentam um risco 76-77% maior de AVC em comparação com mulheres brancas após ajuste para idade.^122^ No Brasil, as doenças cerebrovasculares também estão entre as principais causas de morte e incapacidade. O AVC é mais prevalente em indivíduos de baixo nível socioeconômico e pode ser parcialmente explicado pelo acesso insuficiente a serviços de saúde e menor controle de FR, como HAS, DM2 e tabagismo. Apesar de a mortalidade por AVC no Brasil apresentar uma tendência de declínio, o que pode ser atribuído a melhorias em prevenção, diagnóstico e tratamento, ainda existem desigualdades regionais significativas, com taxas mais altas no Norte e Nordeste do país.

De forma significativa, os desfechos do AVC tendem a ser mais graves em mulheres, com maiores taxas de mortalidade e pior recuperação funcional.^125^

#### 4.3.1. Fatores de Risco Comuns para o AVC

**Figura 4.3:**
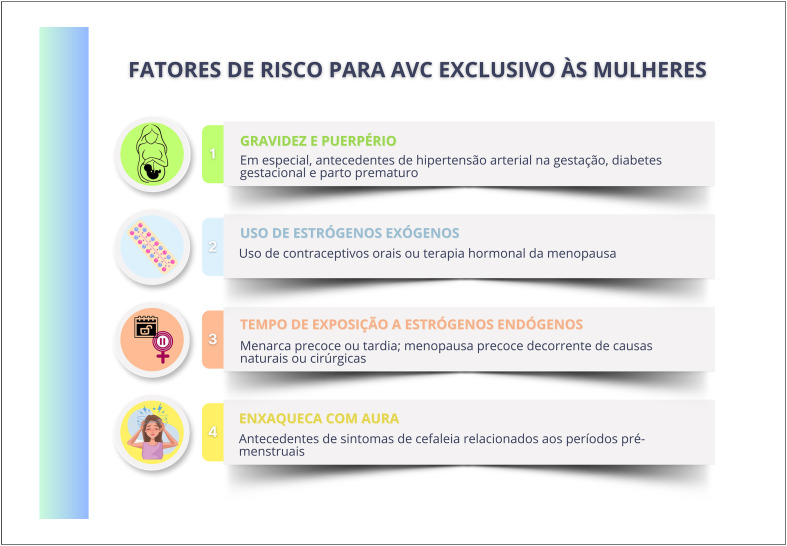
Fatores de risco para acidente vascular cerebral (AVC) exclusivo às mulheres.

No estudo INTERSTROKE, sugeriu-se que dez FR comuns poderiam explicar aproximadamente 90% do risco atribuível da população para AVC.^126^ A HAS é o FR mais prevalente, e estudos recentes demonstraram um impacto negativo maior nas mulheres. O DM2 é um importante FR para o AVC isquêmico e hemorrágico, com maior risco nas mulheres.^122^ Dados recentes não identificaram diferenças entre homens e mulheres quanto ao impacto da DLP na prevalência de AVC, e há dados controversos quanto ao incremento de AVC isquêmico e hemorrágico na população feminina na presença de obesidade. O tabagismo tem relação direta com a prevalência de AVC e esse impacto é maior nas mulheres.^127^ Alguns FR são exclusivos às mulheres (Figura 4.3).

### 4.4. Insuficiência Cardíaca

Alterações fisiológicas da menopausa influenciam diversos órgãos e sistemas, sendo o sistema CV um dos mais afetados.^55^ Em comparação com mulheres na pré-menopausa, na pós-menopausa são mais frequentes a disfunção sistólica e diastólica do ventrículo esquerdo (VE), maior espessura relativa da parede, remodelamento concêntrico do VE e alteração do relaxamento ventricular.^128^ Estudo de coorte com mais de 1,4 milhão de mulheres na pós-menopausa mostrou que a menopausa se associou a um risco 33% maior de insuficiência cardíaca comparado à ausência desse histórico após ajuste para os FRCV, e que a idade mais precoce da menopausa aumenta a incidência de insuficiência cardíaca gradualmente.^129^

**Figura 4.4:**
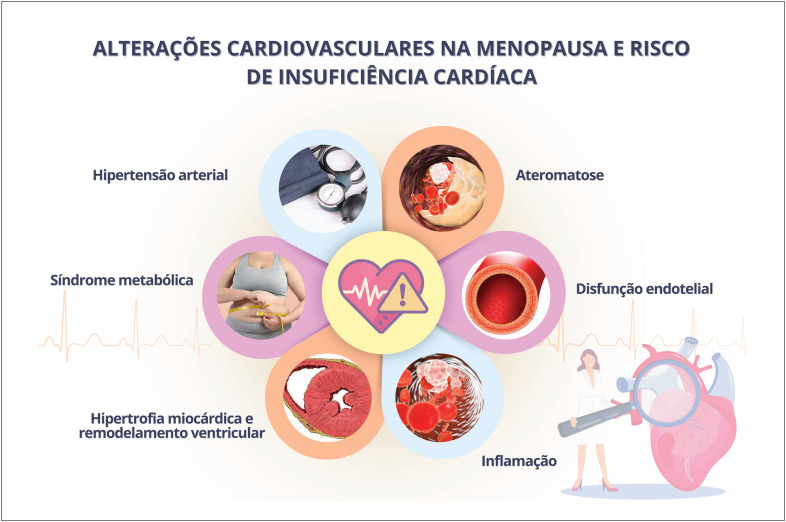
Alterações cardiovasculares na menopausa e risco de insuficiência cardíaca. Adaptado de Muka et al.^132^

As alterações CV da pós-menopausa concorrem com múltiplos fatores para o risco de desenvolvimento de insuficiência cardíaca.^130^ A deficiência de estrogênio predispõe ao maior risco pelo seu efeito direto ou indireto na disfunção diastólica, sendo essa uma das principais causas de insuficiência cardíaca em mulheres. À medida que os níveis de estrogênio diminuem, mulheres na menopausa tornam-se mais predispostas a FR cardiometabólicos.^131^ A perda de estrogênio na pós-menopausa pode ativar o SRAA que, por sua vez, ativa vias de sinalização intracelular, resultando em disfunção endotelial, inflamação, dano vascular, remodelamento ventricular esquerdo e eventual disfunção diastólica, levando à insuficiência cardíaca (Figura 4.4).^132^

Quanto maior o período de privação estrogênica na menopausa precoce, maior o risco cumulativo de insuficiência cardíaca, principalmente na presença de FRCV prévios, conforme demonstrado em alguns estudos.^133,134^

Devido ao fardo da insuficiência cardíaca nas mulheres e ao aumento na sua prevalência, são necessárias pesquisas para estabelecer a causalidade e compreender os mecanismos subjacentes ao início precoce da menopausa e como essa pode contribuir para a insuficiência cardíaca. Essas informações são relevantes para implementar intervenções visando melhorar a saúde CV das mulheres na pós-menopausa.

### 4.5. Tromboembolismo Venoso

O TEV, incluindo a trombose venosa profunda (TVP) e a embolia pulmonar (EP), apresenta uma incidência de cerca de 1 por 1.000 mulheres-ano na pós-menopausa. Aproximadamente 10% dos casos podem ser fatais, sendo a EP a principal causa de morte.^135^

A menopausa leva a alterações no sistema CV que potencialmente contribuem para o aumento do RCV, sem haver uma associação direta dessa fase da vida da mulher com um maior risco de TEV. Entretanto esse risco aumenta exponencialmente com a idade e pode, ainda, estar associado com a maior prevalência de outros FR para TEV, como obesidade, câncer, hospitalização ou outras comorbidades presentes em mulheres idosas.^136^

Importante ressaltar que em mulheres com risco aumentado para TEV, aquelas com idade acima de 60 anos de idade e/ou com menopausa há mais de 10 anos devem evitar a THM pela potencialização do risco de eventos tromboembólicos.^136-138^

### 4.6. Arritmias

Com base em recentes dados observacionais, fatores reprodutivos (menarca, IOP e menopausa precoce, perdas recorrentes gestacionais, momento e número de gestações) associam-se a risco de DCV nas mulheres, sendo a menopausa o mais forte marcador de RCV. Quando ocorre de forma prematura, ou seja, antes dos 40 anos de idade, aumenta o risco de infarto do miocárdio (IM), AVC, insuficiência cardíaca e mortalidade CV. Quanto às arritmias, porém, há poucos dados na literatura correlacionando-as com menopausa. A fibrilação atrial (FA) é uma das doenças mais comuns do envelhecimento e está associada a múltiplos fatores como eventos CV, inflamação, maior frequência de trombose, desregulação hormonal, sugerindo correlação entre menopausa e risco aumentado de FA.^139^

#### 4.6.1. Fibrilação Atrial e Menopausa

**Figura 4.5:**
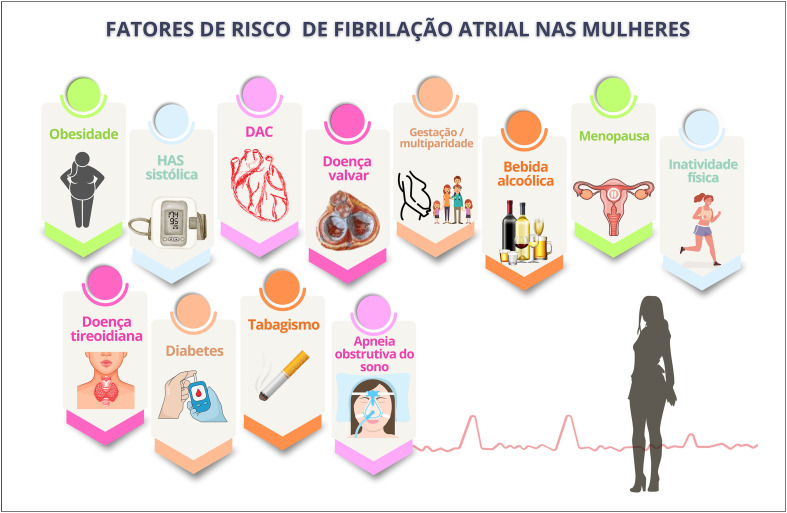
Fatores de risco para fibrilação atrial nas mulheres. DAC: doença arterial coronariana; HAS: hipertensão arterial sistêmica; THM: terapia hormonal da menopausa.^13^

Estima-se que 29,4 milhões de mulheres tenham FA ao redor do mundo. Embora a incidência seja maior entre homens, mulheres idosas têm mais FA por apresentarem maior expectativa de vida.^140^ A mulher apresenta FR próprios para FA, como HAS sistólica, obesidade, sedentarismo, ingesta excessiva de álcool, doença valvar, multiparidade e DAC (Figura 4.5).^13^

Atualmente, muito se tem questionado quanto ao aumento no risco de FA em mulheres na menopausa ou sob THM.^141^ Um estudo avaliou prospectivamente a relação entre idade da menopausa, THM e incidência de FA. Foram seguidas por 20,5 anos 30.034 mulheres saudáveis sem histerectomia e/ou ooforectomia prévia à menopausa, que se deu próximo aos 50 anos. Foram observados 1.350 eventos de FA, porém a idade de ocorrência da menopausa não agregou risco àquele inerente à própria idade.^142^

Em contrapartida, um estudo realizado em 1.401.175 mulheres na pós-menopausa, com seguimento de 9,1 anos, avaliou a associação entre menopausa precoce, idade da menopausa e risco de FA. Foram observados 44.834 (3,2%) novos casos de FA e a história de menopausa precoce associou-se a risco aumentado de FA. Houve maior tendência de FA quando a idade da menopausa era inferior a 50 anos em comparação com superior a 50 anos, principalmente nas mulheres com menos de 40 anos (IOP). Os resultados mostraram que, quanto mais cedo ocorrer a menopausa, maior o risco de FA, indicando atenção quanto à prevenção e cuidado nesse grupo específico de mulheres.^129^ Recente meta-análise com 9 estudos incluindo 6.255.783 mulheres pós-menopáusicas evidenciou que aquelas com menopausa precoce (antes dos 45 anos) ou prematura (antes dos 40 anos) tiveram risco aumentado de FA quando comparadas àquelas com menopausa em idade habitual. No entanto, o exato mecanismo ainda não está elucidado e requer estudos prospectivos futuros.^143^ Um estudo em que 16.729 mulheres foram seguidas por 8,5 anos mostrou que 3.943 desenvolveram FA e que níveis de proteína C reativa e interleucina (IL) estiveram associados a FA, porém sem relação com IL-1β na análise multivariada.^144^

Na menopausa, ocorrem mudanças comportamentais importantes, onde estresse, ansiedade, insônia e sintomas depressivos podem ativar fatores inflamatórios e neuro-hormonais, potenciais ao desenvolvimento de FA. Um estudo recente examinou a correlação entre citocinas e incidência de FA em mulheres pós-menopáusicas, onde foram avaliadass 83.736 mulheres com idade média de 63,9±7,0 anos, seguidas por 10,5±6,2 anos, observando-se 23.954 casos de FA. Insônia e eventos estressantes da vida foram os maiores fatores psicossociais associados à arritmia.^145^

Fatores como idade do início da menopausa, FR associados como estresse, ansiedade, depressão e qualidade do sono, precisam ser considerados a fim de atentar para medidas que visem reduzir o RCV, notadamente a insuficiência cardíaca e FA.^143^

## 5. Menopausa e Risco de Morbidade e Mortalidade por Outras Doenças

### 5.1. Câncer

A intersecção entre DCV, câncer e menopausa representa um campo crescente de interesse pela medicina. As DCV e o câncer são as principais causas de morte no mundo em homens e mulheres, aumentando substancialmente após a menopausa, tendo em comum alguns FR como a idade, obesidade, tabagismo, história familiar e dieta.^13,55^

Tanto a obesidade como a SM estão associadas ao aumento da incidência de DM2, DCV e câncer de mama (pós-menopausa), além de outros cânceres.^146^

O RCV em mulheres na pós-menopausa tratadas de câncer de mama é maior que em mulheres sem câncer de mama. Sobreviventes de câncer de mama na pós-menopausa mostraram uma forte associação com SM, DM, doença aterosclerótica, hipertrigliceridemia, HAS e obesidade abdominal, principais FRCV quando comparadas com mulheres na pós-menopausa sem câncer de mama. Nas mulheres na pós-menopausa com câncer de mama em estágio inicial, o risco aumenta acentuadamente, fazendo com que as taxas de mortalidade por DCV em 10 anos sejam semelhantes às taxas de mortalidade pelo próprio câncer.^147^

O aumento do RCV em mulheres menopáusicas com câncer não é apenas devido ao inadequado controle dos FRCV, mas também decorre do tratamento do câncer pelos seus efeitos secundários cardiotóxicos, como disfunção ventricular, HAS, arritmias, isquemia miocárdica, distúrbios valvares, doença tromboembólica, hipertensão pulmonar e pericardite, além de ateromatose.

A Qt com antraciclinas e trastuzumabe pode causar disfunção cardíaca a curto, médio e longo prazo. A radioterapia no hemitórax esquerdo pode levar a efeitos CV secundários, como aterosclerose coronariana, que podem ocorrer mais de 5 anos após a exposição, com o risco persistindo por até 30 anos. A terapia hormonal com inibidores da aromatase também aumenta o risco de doença aterosclerótica.^148^

Os efeitos CV tardios do câncer desenvolvem-se ao longo de várias décadas, o que para muitas mulheres pode se sobrepor a eventos reprodutivos e do ciclo de vida. Assim, as mulheres necessitam de cuidados cardio-oncológicos longitudinais que antecipem e respondam à evolução do seu RCV.^149^

Em mulheres com câncer, a menopausa pode ser precoce, gradual ou rápida, dependendo da reserva ovariana basal, da gonadotoxicidade e da duração da exposição aos agentes cancerígenos (terapia oncológica e/ou terapia endócrina).^150^

Mulheres sobreviventes de câncer infantil correm risco de desenvolver menopausa precoce decorrente de IOP após tratamento oncológico.^151^

O RCV é maior na menopausa precoce devido à privação prolongada de estrogênio endógeno, levando a uma variedade de efeitos metabólicos e na função vascular, incluindo intolerância à glicose, DLP, HAS e disfunção endotelial.^152^

A IOP não só confere risco de DIC após ajuste para FR convencionais, mas também prevê piores desfechos isquêmicos e maior mortalidade.^153^

A menopausa induzida pelo tratamento oncológico pode ser causada por ooforectomia cirúrgica bilateral, Qt, radioterapia para a pelve e/ou terapia supressora hormonal. A ooforectomia bilateral causa menopausa aguda e permanente, sendo que, antes dos 50 anos de idade, aumenta o risco de DCV global (risco relativo [RR]: 4,55; IC 95%, 2,56-8,01), insuficiência cardíaca e AVC.^154^

Os quimioterápicos e radioterapia nos ovários podem levar a disfunção ovariana e consequente menopausa secundária, que pode ser temporária ou permanente, dependendo da idade da paciente, do tipo e da dose do medicamento, duração do tratamento e, no caso da radioterapia, do local da aplicação e da dose realizada.^155^

Algumas terapias supressoras hormonais ou terapias endócrinas, sejam com inibidores da aromatase ou com moduladores seletivos de receptores de estrogênio, podem impedir temporariamente a ovulação e causar menopausa temporária. Tratamento com tamoxifeno e inibidores da aromatase por 5 anos melhoraram a taxa de sobrevivência de 20 anos até 85%, com risco de recorrência de 22%.^156^

A terapia endócrina é um tratamento comum, pois 65–70% de todas as pacientes com câncer de mama precoce e metastático desenvolvem doença com receptor hormonal positivo. A terapia endócrina envolve a redução dos níveis ou a inibição de sua atividade biológica, parando/retardando ou prevenindo o crescimento do câncer. Moduladores seletivos de receptores de estrogênio (tamoxifeno, toremifeno) ou inibidores da aromatase (letrozol, anastrozol ou exemestano) são recomendados no câncer de mama precoce de acordo com o *status* da menopausa, comorbidades e risco de recidiva da doença.^155^

O tamoxifeno é a terapia endócrina de escolha para mulheres na pré-menopausa, enquanto as estratégias em mulheres na pós-menopausa podem incluir tamoxifeno, inibidores da aromatase ou uma combinação sequencial, com avaliação cuidadosa dos benefícios e gerenciamento dos riscos de toxicidade.^148^

O uso de inibidores da aromatase aumenta o risco de DLP, SM, HAS, insuficiência cardíaca e IM.^156^ No estudo Arimidex, Tamoxifen, Alone or in Combination (ATAC), pacientes portadoras de DIC pré-existente tratadas com anastrozol apresentaram mais eventos CV (17% vs. 10%) e elevação do nível de colesterol (9% vs. 5%) do que aquelas tratadas com tamoxifeno.^157^ O aumento significativo do risco de doença tromboembólica foi consistentemente demonstrado com tamoxifeno, que não é, portanto, recomendado em pacientes com risco aumentado de trombose. Os riscos de doença tromboembólica, hipercolesterolemia e DCV devem ser discutidos com as pacientes, embora se reconheça que os benefícios absolutos da prevenção da recorrência do câncer de mama geralmente superam os RCV.^158^

Concluindo, existe associação entre câncer e aumento do RCV em mulheres pós-menopáusicas, assim como barreiras psicossociais e físicas distintas no acesso aos cuidados CV.

Portanto, torna-se importante que mulheres na menopausa e com histórico de câncer sejam monitoradas por profissionais especializados, para avaliar seu RCV, realizar exames complementares e recomendar medidas preventivas e, se necessário, medicamentosas com o objetivo de reduzir a morbimortalidade CV nessas pacientes.

### 5.2. Demência

O envelhecimento da população mundial tem como consequência o aumento da prevalência e da incidência de doenças crônicas e neurodegenerativas.

Atualmente, estima-se que existam 50 milhões de pessoas acometidas por alguma forma de demência no mundo e 10 milhões de novos diagnósticos por ano. No Brasil, estima-se que existam cerca de 1,7 milhão de idosos com demência, com uma prevalência de aproximadamente 1.036/100 mil habitantes.^159^ As estimativas globais de prevalência da demência são de até 7% dos indivíduos com mais de 65 anos e estimativas futuras apontam maior prevalência nos países de baixa e média renda.^160,161^

**Figura 5.1:**
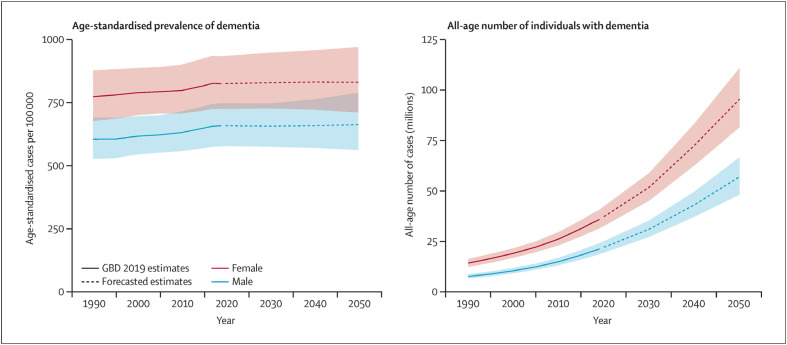
Tendências estimadas na prevalência global de demência padronizada por idade (A) e número de casos em todas as idades (B). Fonte: GBD 2019.^162^

Dados do *Global Burden of Disease* (GBD) mostram que, em 2019, havia mais mulheres com demência do que homens (razão de 1.69 mulher/homem) e que esse padrão continuaria em 2050 (razão de 1.67 mulher/homem)^162^ (Figura 5.1). Apesar da maior prevalência de riscos vasculares nos homens do que nas mulheres, esses padrões existem, sugerindo mecanismos de neutralização potencialmente fortes que impulsionam essas desigualdades. Embora a diferença entre os sexos possa ser explicada em parte pela maior expectativa de vida nas mulheres, evidências anteriores sugerem potenciais diferenças entre os sexos também em mecanismos biológicos subjacentes.

**Figura 5.2:**
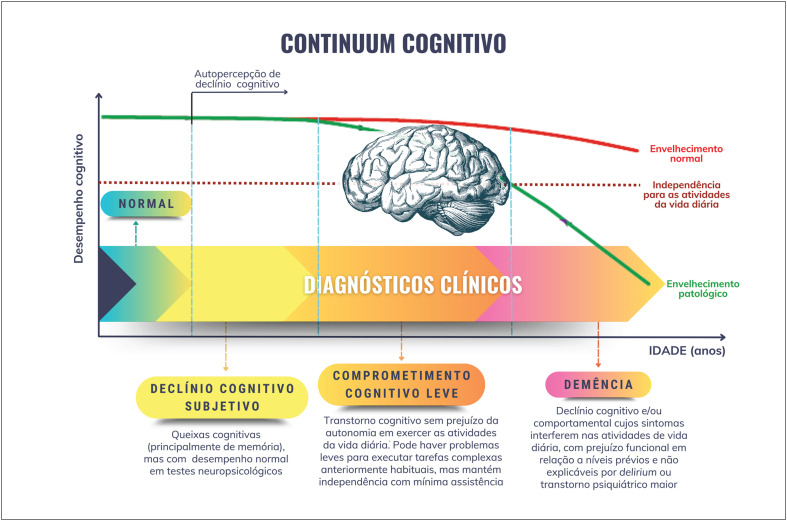
Continuum do declínio cognitivo no envelhecimento normal e patológico.^159^

O declínio cognitivo ocorre em todos os indivíduos com o avançar da idade, manifestando-se desde o comprometimento leve e, portanto, sem prejuízo de sua autonomia ou um declínio subjetivo, onde os testes neuropsicológicos são normais, até o desfecho final, a demência. Esse processo se faz de forma contínua com o envelhecimento normal e em situações patológicas, onde há prejuízo da autonomia em exercer as atividades da vida diária (Figura 5.2).

Dentre as demências degenerativas comuns em idosos,^160^ a doença de Alzheimer é a forma mais prevalente, correspondendo a 70-80% dos casos. A demência vascular corresponde a 15%, estando associada a FRCV. Outras causas são a demência com corpos de Lewy, a degeneração lobar frontotemporal e a doença de Parkinson, essa última correspondendo a 10% dos casos. Deficiência de vitaminas (B12 e tiamina), hipotireoidismo, hidrocefalia de pressão normal, abuso crônico de álcool, disfunção cognitiva relacionada à Qt, massas intracranianas (hematomas subdurais, tumores cerebrais), lesão cerebral traumática e doença psiquiátrica (depressão/ansiedade profunda)^160^ são causas comuns de comprometimento cognitivo leve não neurodegenerativo e demência, podendo ocorrer ao longo da vida. Dentre as demências de causa mista, a doença de Alzheimer associada à demência vascular é a mais comum.^160^

O avanço da idade, o perfil genético (alelo APOE ε4) e a presença de doença vascular sistêmica são os principais FR não modificáveis para o desenvolvimento de demência, sendo que a etnia e o gênero também merecem destaque.^162^ Apesar de a demência não fazer parte do envelhecimento normal, a idade é o maior FR conhecido, sendo que sua incidência aumenta proporcionalmente ao envelhecimento da população. Apesar disso, em alguns países, a incidência de demência por idade reduziu provavelmente devido à melhoria na educação, nutrição, cuidados de saúde e mudanças no estilo de vida. Em relação ao gênero, sua prevalência é maior entre as mulheres, não apenas por constituírem o maior contingente de idosos, mas também por sofrerem maior impacto de FR modificáveis que afetam a reserva cognitiva. Quanto à etnia, muitos FR agrupam-se em torno de desigualdades, que ocorrem particularmente em negros, asiáticos e minoritários, e em populações vulneráveis. O início precoce da doença de Alzheimer associa-se a fatores genéticos, porém o gene mais conhecidamente associado ao aparecimento mais tardio da doença de Alzheimer é o alelo APOE ε4.^162^

Entre os FR modificáveis, destacam-se a baixa escolaridade, HAS, deficiência auditiva, tabagismo, obesidade, depressão, sedentarismo, DM, isolamento social, etilismo, traumatismo cranioencefálico e poluição do ar. Cerca de 40% das demências podem ser prevenidas ou postergadas com a intervenção sobre esses FR. A presença de FR precoces, ou seja, que aparecem antes dos 45 anos de idade, como a menor escolaridade, afeta a reserva cognitiva. Os FR que aparecem na meia-idade (45-65 anos) e na idade avançada (acima dos 65 anos) influenciam a reserva e o desencadeamento de estados neuropatológicos.^162^

#### 5.2.1. Envelhecimento Reprodutivo Feminino e Declínio Cognitivo

A TM é um processo de envelhecimento neuroendócrino da meia-idade que culmina com a senescência reprodutiva, ocorrendo em estágios caracterizados por propriedades endócrinas únicas que impactam as trajetórias de envelhecimento de múltiplos sistemas orgânicos, incluindo o cérebro. Sendo assim, a TM é considerada um estado de transição reprodutiva e neurológica, como evidenciado pelo fato de muitos sintomas da menopausa serem de natureza neurológica, tais como SVM, perturbações do sono, alterações de humor e esquecimento.^163^

Os hormônios esteroides sexuais gonadais, em especial o 17β-estradiol, são conhecidos reguladores da função reprodutiva e neural e, durante a TM, seus níveis diminuem substancialmente no corpo e no cérebro.

A TM tem efeitos pronunciados na estrutura, na conectividade e no metabolismo energético cerebrais e na deposição de proteína amiloide β (Aβ) no cérebro. Estudo de neuroimagem multimodal foi realizado em mulheres em diferentes estágios de TM (pré-, peri- e pós-menopausa) para investigar seus efeitos na estrutura das substâncias cinzenta e branca do cérebro.^163^ Os resultados indicam que a TM impacta significativamente em biomarcadores cerebrais em regiões envolvidas em funções cognitivas de ordem superior. Os efeitos, independentemente da idade e do uso de terapia hormonal, foram específicos do envelhecimento endócrino da menopausa e não do envelhecimento cronológico, conforme determinado pela comparação com homens da mesma idade. Notavelmente, a cognição foi preservada na pós-menopausa, o que se correlacionou com a recuperação do volume da substância cinzenta e a produção cerebral de adenosina trifosfato, destacando potenciais mecanismos compensatórios. Finalmente, a deposição de Aβ foi maior em mulheres na peri- e pós-menopausa portadoras do genótipo APOE-4, indicando efeitos específicos desse gene no risco de doença de Alzheimer com início na perimenopausa.^163^

#### 5.2.2. Conclusão

Pela ausência de tratamentos recentes e eficazes em modificar a evolução da demência, os esforços imediatos para reduzir a sua prevalência no futuro deverão ser direcionados para a prevenção, através de intervenções nos FR modificáveis. Intervenções que alteram a prevalência desses FR podem reduzir em até 40% a esperada prevalência da demência nos próximos anos, segundo resultados da atualização da Comissão Lancet de 2020 sobre prevenção, intervenção e cuidados com a demência.

Concluindo, sugere-se que grandes mudanças na exposição a FR tenham potencial de alterar consideravelmente as estimativas previstas e reduzir o fardo futuro da demência em todo o mundo.^162^

### 5.3. Disfunção Tireoidiana

Os distúrbios da tireoide são significativamente mais comuns em mulheres e sua incidência aumenta com o envelhecimento, já que a produção fisiológica dos hormônios tireoidianos decresce com o avançar da idade.^164^

É provável que quase uma em cada oito mulheres tenha algum tipo de disfunção tireoidiana durante a vida,^165^ sobretudo durante o período de peri- e pós-menopausa.^166^

Existem poucos estudos sobre a relação entre menopausa e função tireoidiana e, portanto, não podemos esclarecer se a menopausa tem efeito na tireoide independentemente do envelhecimento.^166^

Entretanto, a atividade da tireoide é idade-dependente,^167^ uma vez que, com o envelhecimento, ocorre redução da captação de iodo pela glândula, do T4 livre, da síntese de T3 livre e do catabolismo do T4 livre. Embora o T3 reverso aumente, o nível de TSH permanece normal, às vezes com tendência a limites mais elevados.^168^

Diante das evidências bem conhecidas sobre o efeito do *status* da tireoide na função cognitiva, no RCV, na remodelação óssea e na longevidade, não fica difícil entender o risco das disfunções tireoidianas nesse grupo de pacientes.^165,166^

**Figura 5.3:**
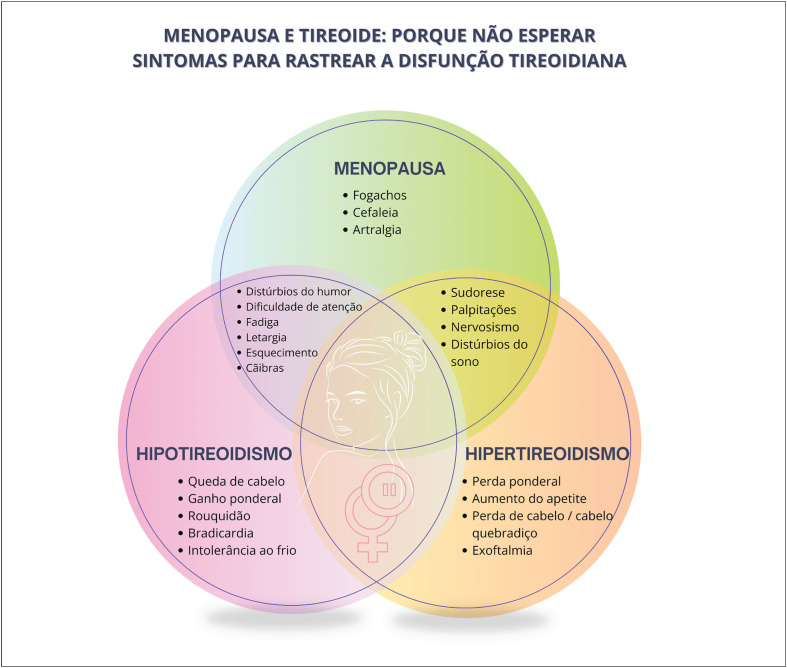
Sobreposição de sintomas das disfunções tireoidianas e da menopausa.

Sendo assim, recomenda-se triagem de rotina para tireoidopatias em mulheres na menopausa, sobretudo porque muitas vezes os sintomas da doença tireoidiana e os da pós-menopausa se sobrepõem, podendo postergar o diagnóstico da disfunção tireoidiana (Figura 5.3).^167^

#### 5.3.1. Hipotireoidismo

A ocorrência de hipotireoidismo, bócio e nódulos tireoidianos aumenta com a idade, sendo o hipotireoidismo mais prevalente em idosos: 2-20% dos quais apresentam algum tipo de hipotireoidismo.^165^

A prevalência do hipotireoidismo subclínico (HipoSC) na população é de aproximadamente 4-10%, sendo maior em mulheres e idosos e inversamente proporcional ao teor de iodo na dieta.^169^ Os riscos potenciais do HipoSC em idosos incluem progressão para hipotireoidismo manifesto, efeitos CV, DLP e efeitos neurológicos e neuropsiquiátricos.

Fadiga, letargia, esquecimentos, dificuldade de atenção ou concentração e distúrbios de humor são características comuns tanto ao hipotireoidismo quanto à menopausa, fazendo com que, muitas vezes, o hipotireoidismo passe despercebido (sintomas ficam atribuídos exclusivamente à menopausa, a efeitos colaterais de medicamentos ou ao próprio envelhecimento).^166^

Entre as causas de hipotireoidismo, a deficiência de iodo e a doença autoimune se destacam. No entanto, iodoterapia, radioterapia para malignidade da cabeça e pescoço e hipotireoidismo central por tumores hipofisários ou hipotalâmicos devem ser mencionados como causa em mulheres, principalmente após a menopausa.^165,170^

Em geral, o HipoSC é assintomático nessa população e não está associado a efeitos na função cognitiva, depressão ou ansiedade. Também não há evidências consistentes sobre as consequências na estrutura cardíaca e nas funções sistólica e diastólica em estudos populacionais.^169^ Há evidências consistentes de associação de HipoSC com risco de DIC, especialmente para valores de TSH ≥ 10 mU/L, apesar de não serem observados esses dados em pacientes acima de 65 anos.^169^ Ainda é incerto se mulheres de meia-idade com HipoSC devem ser tratadas.^13^ As Diretrizes da Sociedade Latino-Americana de Tireoide não recomendam o tratamento de rotina para idosos com HipoSC se o TSH for < 10 mU/L. Também não recomendam o tratamento do HipoSC para melhora da função cognitiva em idosos, podendo o tratamento, no entanto, ser considerado individualmente.^170^

Devido à falta de dados em estudos robustos que demonstrem benefícios quanto ao RCV e ao risco de mortalidade, o tratamento do HipoSC permanece controverso. Pode ser considerado no HipoSC persistente e após confirmação dos níveis séricos de TSH ≥ 10 mU/L após 3 a 6 meses, pelo maior risco de progressão para hipotireoidismo manifesto, insuficiência cardíaca, DAC e mortalidade.^169^ Estudos de coorte mostram evidência indireta de benefícios do tratamento do HipoSC sobre RCV e mortalidade, além de efeito favorável no CT em pacientes com HipoSC e TSH > 10 mU/L.^169,171,172^

Uso de medicamentos e doenças hepáticas e renais podem afetar o metabolismo dos hormônios tireoidianos ou alterar as proteínas de ligação a esses hormônios. Portanto, a monitorização do TSH sérico deve ser feita com o uso ou descontinuação de estrogênios e andrógenos orais uma vez que tais medicamentos podem alterar a necessidade de levotiroxina, aumentando a globulina de ligação à tireoide que, por sua vez, reduz o T4 livre.^164,166^

A terapia com levotiroxina pode induzir melhora relevante em alguns parâmetros CV, como aumento do débito cardíaco, diminuição da resistência vascular sistêmica e do volume diastólico final, efeitos mais evidentes na doença clínica do que na subclínica. Por outro lado, pode aumentar o consumo de oxigênio e, assim, induzir isquemia miocárdica em pacientes com DAC subjacente.^170,172^ Evidências recentes sugerem um aumento do risco de fratura em pacientes > 70 anos em doses usuais. Portanto, em pacientes idosos e nos portadores de DIC ou insuficiência cardíaca, a diretriz sugere iniciar levotiroxina em doses de 12,5-25 µg/dia, especialmente em pacientes com HipoSC.^170^

#### 5.3.2. Hipertireoidismo

O hipertireoidismo ocorre numa proporção de 5 mulheres:1 homem. Sua prevalência (aproximadamente 1,3%) aumenta para 4-5% em mulheres idosas, sendo o bócio nodular tóxico a causa mais comum nessa faixa etária. Porém, o hipertireoidismo induzido por drogas, como contraste e amiodarona, deve ser lembrado como causa.^166^

O hipertireoidismo em pacientes idosos pode ser apático em vez da apresentação clássica de hiperatividade do sistema simpático, com tremores e palpitações.^166^ Em estudos transversais, pacientes idosos tiveram um risco reduzido para sintomas clássicos (intolerância ao calor, tremor, nervosismo) e maior prevalência de perda de peso e falta de ar em comparação com pacientes mais jovens, assim como uma taxa mais alta de oftalmopatia moderada a grave e FA. No entanto, a taquicardia pode estar ausente por doença do sistema de condução concomitante.^166^

Os sintomas típicos do hipertireoidismo mimetizam os sintomas relacionados à menopausa, mas, com o avanço da idade, os sintomas clínicos diminuem.

Slopien *et al*.^167^ observaram que a concentração sérica de TSH tem correlação negativa com sintomas como sudorese, palpitações e fraqueza, enquanto a de T4 livre tem correlação positiva com palpitações, parestesias e nervosismo.

**Figura 5.4:**
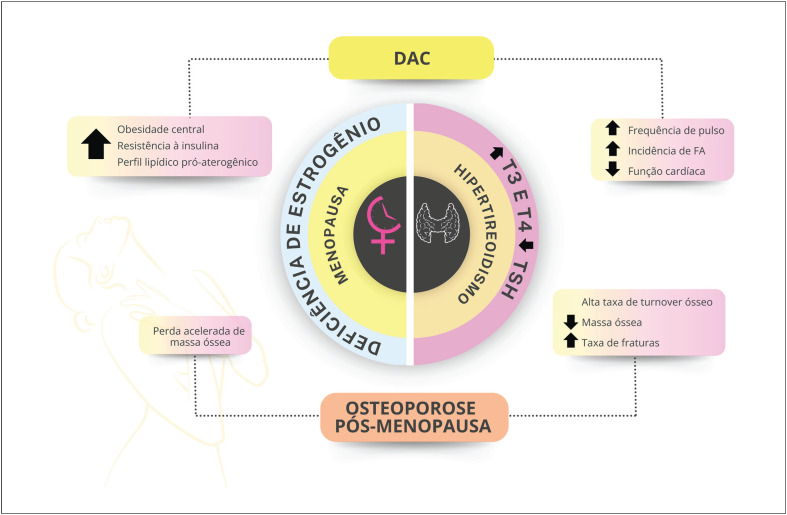
Menopausa e hipertireoidismo: acúmulo de riscos. DAC: doença arterial coronariana; FA: fibrilação atrial.

No hipertireoidismo, os ciclos de remodelação óssea são reduzidos, resultando em uma alta taxa de *turnover* ósseo. Com a reabsorção óssea superando a mineralização, há perda de aproximadamente 10% de massa óssea por ciclo. Além disso, ocorre redução da absorção de cálcio pelo intestino e aumenta a excreção renal de cálcio, resultando num balanço negativo desse eletrólito. Há evidências claras de que tanto o hipertireoidismo manifesto quanto o hipertireoidismo subclínico (HiperSC) aumentam o risco de osteoporose, especialmente na na pós-menopausa (Figura 5.4).^164^

Embora o tratamento do hipertireoidismo manifesto seja sempre recomendado, o manejo do HiperSC é menos claro. Vários estudos mostraram efeitos deletérios semelhantes tanto no HiperSC quanto no manifesto. Meta-análise recente encontrou risco aumentado de DAC no HiperSC (HR = 1,44 [1,06; 1,94]).^164^ Os efeitos da supressão de TSH a longo prazo incluem aumento da FC em repouso, arritmias frequentes (especialmente FA), bem como função cardíaca reduzida. Esse maior RCV estende-se também para supressão de TSH em pacientes em tratamento com levotiroxina.^164^

Estudos mostraram um aumento da mortalidade CV no HiperSC^166^ e dados recentes indicam que pacientes portadores dessa patologia apresentam risco de desenvolver insuficiência cardíaca, especialmente os mais idosos e com menores níveis de TSH.^166^

Um estudo populacional mostrou que o HiperSC aumentou o risco de AVC em indivíduos com mais de 50 anos (HR = 3,39), embora uma meta-análise tenha sido inconclusiva.^166^

#### 5.3.3. Autoimunidade Tireoidiana e Insuficiência Ovariana Prematura

A etiologia autoimune constitui aproximadamente 5% dos casos de IOP, sendo a doença autoimune da tireoide presente em 14-27% das mulheres no primodiagnóstico. Assim, recomenda-se determinar os níveis de TSH e a presença de anticorpos anti-peroxidase tireoidiana (anti-TPO) nessas pacientes.^166^

### 5.4. Depressão e Ansiedade

**Figura 5.5:**
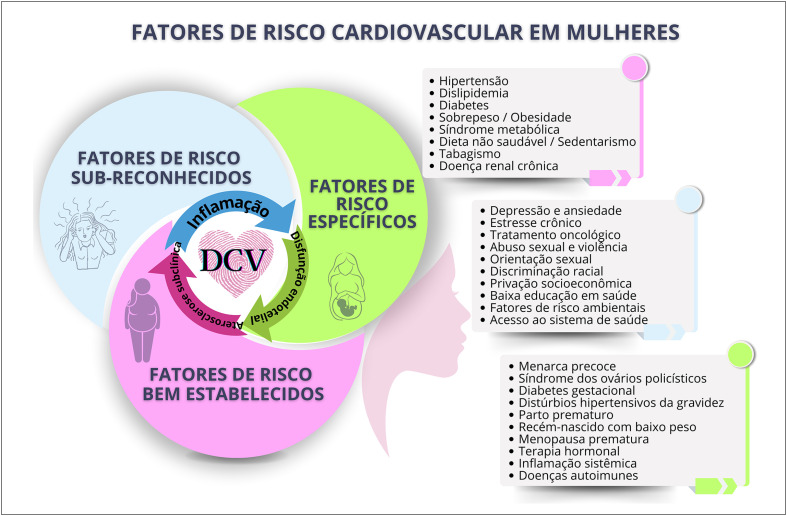
Correlação entre fatores de risco específicos, sub-reconhecidos e bem estabelecidos da doença cardiovascular (DCV) na mulher.^173^

Estudos têm mostrado a importância dos FR clássicos nas DCV. Nas mulheres, porém, os FR específicos do gênero e os sub-reconhecidos, como depressão e ansiedade, também têm grande impacto no RCV^13^ (Figura 5.5).

Um em cada cinco adultos apresenta algum tipo de distúrbio psiquiátrico ao longo da vida. Nas mulheres, porém, os fatores intrínsecos e extrínsecos ao gênero têm o poder de intensificar a ocorrência desses distúrbios, que estão diretamente relacionados ao aumento do RCV nessa população.^173,174^

Estudos recentes correlacionam doenças mentais, como transtorno de ansiedade generalizada e depressão, com o aumento do RCV, devido às alterações metabólicas desencadeadas por estresse e injúrias do cotidiano em consequência de tais comorbidades.

Padrões distintos de alterações nos hormônios sexuais e na distribuição de gordura corporal, de lipídios e de lipoproteínas ao longo da menopausa relacionam-se com o potencial de acelerar o desenvolvimento das DCV nessa fase de vida da mulher.^55^

Mulheres portadoras de transtorno de ansiedade generalizada ou depressão podem desenvolver algum grau de prejuízo social devido ao próprio mecanismo de adoecimento, o que pode ser intensificado de maneira natural em certas fases da vida, como na menopausa. Atualmente essa correlação tem sido estudada com uma frequência cada vez maior. Importante ressaltar que sintomas provocados pelo climatério, como SVM, distúrbios do sono e da libido e alterações cognitivas, podem tanto intensificar quadros de comorbidades mentais, como mascarar o seu diagnóstico, tornando o tratamento tardio.^173^

Em relação à perimenopausa, "gatilhos" como mudanças de carreira, relacionamentos, conscientização do envelhecimento, transformação da aparência física, doença pessoal e familiar e síndrome do ninho vazio, podem corroborar com sentimentos de tristeza, menos-valia, insuficiência, medo e frustração, aflorados pelas síndromes depressivas/ansiosas. O mecanismo fisiopatológico envolve o sistema nervoso autônomo e o eixo hipotálamo-hipófise-adrenal, levando ao aumento de cortisol e catecolaminas e a alterações da homeostase correspondentes.^173,174^ Além do mais, a presença de transtornos psiquiátricos associa-se com comportamentos pouco saudáveis, que contribuem para elevar o RCV, como a compulsão alimentar, tabagismo, etilismo, sedentarismo e menor adesão ao tratamento de comorbidades.^175^

O papel prejudicial do transtorno de ansiedade generalizadae da depressão na saúde CV foi demonstrado por estudos recentes, evidenciando que pessoas ansiosas têm um risco de morte cardíaca duas vezes maior em relação à população geral.^176^ Em contraponto, o apoio emocional está descrito como potencial efeito cardioprotetor.^175^

Portanto, o acompanhamento longitudinal da saúde da mulher por equipe multiprofissional é de fundamental importância para se diferenciar a fase de menopausa saudável de uma associada à depressão ou ansiedade. A intervenção precoce com estratégias de prevenção, diagnóstico e tratamento adequados visam à diminuição do RCV na mulher nessa fase de vida.^174,176^

### 5.5. Osteoporose e Menopausa

O comprometimento da saúde CV e da saúde óssea está diretamente relacionado à idade em que se instala a menopausa. Existe uma forte correlação de IOP e menopausa precoce com o aumento de RCV, osteoporose, fraturas e mortalidade nas mulheres acometidas.^177^ Seu mecanismo fisiopatológico está relacionado ao efeito direto do estrogênio sobre a função endotelial e sobre o metabolismo ósseo, uma vez que o impacto de FR tradicionais, como HAS, DLP, obesidade e hiperglicemia, frequentes nas mulheres que entram na menopausa em idade habitual, é menor nessas condições.^177^

Os efeitos sistêmicos da privação estrogênica aumentam o risco de depressão, demência e osteoporose e, consequentemente, de mortalidade por todas as causas. A IOP e a menopausa precoce podem estar relacionadas a fatores genéticos ou ambientais e, pelo aumento do risco de desfechos adversos à saúde da mulher, devem ser consideradas um marcador de risco.^132^

O estrogênio tem importante papel na homeostase dos ossos, através de seus receptores localizados em células precursoras de osteoblastos e osteoclastos, mantendo assim o balanço entre reabsorção e formação óssea. O estrogênio suprime a reabsorção e a velocidade de remodelamento ósseo através da redução do número de osteoclastos e da sua vida útil, via apoptose de osteoclastos, mantendo, assim, a massa óssea. Além disso, parece atenuar a apoptose de osteoblastos e osteócitos.^178^

A perda do estrogênio na menopausa leva a remodelamento ósseo negativo e consequente perda óssea, como evidenciado em estudos que mostram aumento da formação de osteoclastos e reabsorção óssea, em parte como resultado do aumento de apoptose de osteócitos.^178^ Portanto, quanto mais precoce for a menopausa, maior risco de osteoporose, fraturas e aumento de mortalidade por todas as causas.^177^

Em mulheres menopáusicas, várias intervenções têm sido propostas para otimizar a sua saúde óssea e CV. Mudanças no estilo de vida, como atividade física regular, dieta equilibrada, suplementação alimentar e controle de peso são fundamentais. A THM deve ser indicada para mulheres com IOP, porém existe pouca evidência em relação ao seu uso nas mulheres com menopausa precoce. Nas que entram na menopausa em idade habitual, a THM pode ser indicada para prevenção de osteoporose, especialmente na presença de SVM associados ao período.^179^

**Figura 5.6:**
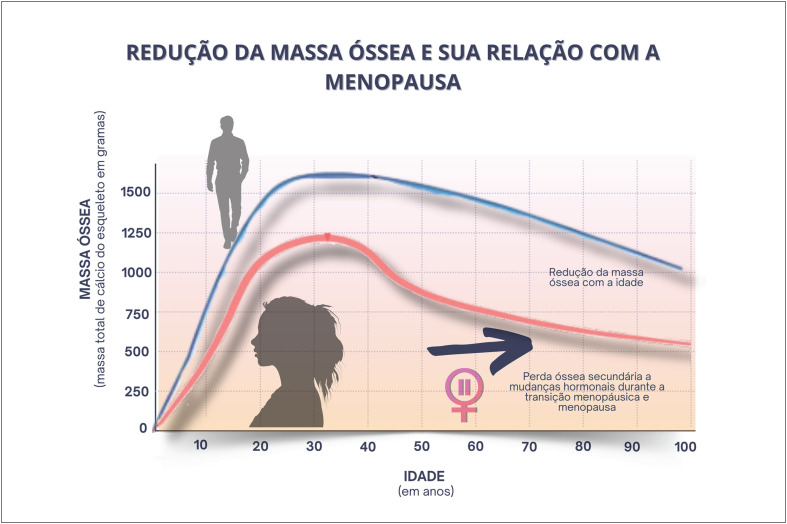
Massa óssea em homens versus mulheres. Note que, em homens, há redução da massa óssea com o avançar da idade, enquanto, em mulheres, observa-se um declínio mais acentuado relacionado à transição menopáusica/menopausa.

A Figura 5.6 ilustra a massa óssea em homens e mulheres, denotando perda óssea mais acentuada nas mulheres após a menopausa.

## 6. Risco Cardiovascular e Hormônios Sexuais

### 6.1. Introdução

As DCV são a principal causa de mortalidade nas mulheres e um constante empenho de força-tarefa multidisciplinar tem sido realizado para mudança desse cenário. FR tradicionais para DCV, como HAS, DM, obesidade, DLP e tabagismo, são reconhecidos em ambos os sexos, sendo, entretanto, crucial destacar FR específicos, que ainda não recebem a devida atenção no momento da anamnese médica.^180,181^ Neste capítulo serão abordados os temas que devem ser considerados nos algoritmos de estratificação do RCV da mulher, cis- ou não, destacando-se: menarca precoce, contracepção hormonal, SOP, uso de testosterona e hormonioterapia na transexualidade.

### 6.2. Menarca Precoce

No Brasil, a idade média de menarca é de 12,4 anos e pode ser influenciada por raça, nível socioeconômico, fatores comportamentais, climáticos, genéticos, além de sobrepeso/obesidade.^182^

A menarca antes dos 10 anos é considerada precoce, mas, quando acontece antes dos 12 anos, já é relacionada a maior RCV, com maior frequência dos FR, mais eventos CV, principalmente DIC e AVC, além de maior mortalidade geral na vida adulta.^183-185^

Fatores relacionados à nutrição fetal, sobrepeso e maior IMC na infância foram associados a menarca precoce e à SM na vida adulta.^183,184,186^ Entretanto, após ajuste para IMC, a menarca precoce está associada com níveis desfavoráveis de insulina, glicemia, hemoglobina glicada, PA e pior perfil lipídico na vida adulta. Ademais, foi registrado maior risco de HAS, DM e doença hepática gordurosa não alcoólica.^183,184,187^

Cada ano de atraso da menarca ocasionou redução de 2% a 4% na mortalidade por todas as causas.^184^ Entretanto, um estudo com 34 mil mulheres mostrou que o tabagismo elimina essa proteção e as não tabagistas tiveram redução da incidência de HAS, DM, obesidade e insuficiência cardíaca.^188,189^

O estudo WISE avaliou 648 mulheres americanas e considerou o tempo em que foram expostas ao estrogênio endógeno (gravidez) ou ao estrogênio exógeno (contraceptivo oral). Apesar de observar aumento do RCV nas pacientes com menarca precoce, principalmente quando anterior aos 10 anos de idade, não conseguiu mostrar diferença na incidência de DCV de acordo com a exposição estrogênica ao longo da vida.^190^

Paradoxalmente, a menarca tardia, após os 17 anos, também parece exercer influência no aumento do RCV.^190,191^ As hipóteses sugerem que o menor tempo de exposição ao estrogênio ao longo da vida e a SOP podem explicar parcialmente o aumento do RCV.^180,184^ Contudo, o assunto ainda é controverso e merece mais investigações.^188,190^

Incluir a idade da menarca na investigação da DCV é uma janela de oportunidade para estratificação de risco, permitindo a implementação de medidas preventivas mais ostensivas, incluindo mudança no estilo de vida e, quando necessário, a terapêutica farmacológica para reduzir eventos CV.

### 6.3. Contraceptivos Hormonais

O desenvolvimento da contracepção hormonal foi aclamado como uma das mais importantes conquistas de saúde pública do século 20. Desde 1960, a "pílula" tem sido usada por milhões de mulheres para prevenção da gravidez indesejada e suas consequências, como aborto inseguro e morte materna por todas as causas.

A contracepção hormonal está disponível em diferentes vias de administração e apresenta-se sob duas formas de composição: combinação do estrogênio (geralmente etinilestradiol – EE) com progestagênio (derivado da progesterona ou testosterona); e progestagênio isolado. Vale destacar que o anticoncepcional hormonal combinado oral (AHCO) é a forma mais utilizada de contracepção hormonal reversível.

#### 6.3.1. Inovação da Contracepção Hormonal e Risco Cardiovascular

Nos anos 60, as evidências científicas mostravam uma "robusta" associação entre doses altas de EE contidas nos AHCO e a ocorrência de tromboembolismo, IM e AVC.^192^

Admite-se que os principais efeitos adversos CV associados aos AHCO derivam da ação do estrogênio no metabolismo lipídico e na hemostasia, ambos mediados pelos receptores hepáticos do estrogênio.^193,194^ Esses indicadores incentivaram a produção de AHCO contendo doses mais baixas de EE, com objetivo de diminuir RCV.^195^

Os progestagênios derivados da testosterona (noretindrona-1^a^ geração, levonorgestrel-2^a^ geração) contidas nos AHCO estão associadas a efeitos colaterais androgênicos com impacto negativo no metabolismo dos carboidratos e lipídios, enquanto os progestagênios derivados da progesterona e espironolactona (clormadinona, drospirenona-4^a^ geração) apresentam ligação mais seletiva aos receptores de progesterona, reduzindo os efeitos colaterais androgênicos, estrogênicos e dos glicocorticoides.^196^

Recentemente, formulações dos AHCO, valerato de estradiol com dienogeste-4^a^ geração e estradiol com acetato de nomegestrol-4^a^ geração, substituíram os hormônios sintéticos por componentes esteroides análogos às moléculas endógenas. Essa inovação pressupõe que esses compostos mais semelhantes aos hormônios naturais devam apresentar um perfil de segurança CV mais favorável.^197^

#### 6.3.2. Contraceptivos Hormonais e Doença Cardiovascular

A associação entre uso de contraceptivos hormonais e DCV é um assunto complexo e ainda motivo de debate e investigações. A falta de evidências e estudos de alta qualidade resultam em conclusões inconsistentes e contraditórias.^198^ Enfim, selecionou-se para este documento estudos que merecem destaque, apesar das limitações.

O risco de IM em usuárias de AHCO foi investigado numa meta-análise de 24 estudos, que mostrou RR de 1,6 (IC 95%, 1,3-1,9) proporcional ao aumento da dose de EE (> 50 mcg) mas não modificado pelo tipo de progestagênio (drospirenona *versus* levonorgestrel).^199^ Uma segunda meta-análise de 11 estudos realizados após a década de 90 constatou um risco agregado de 1,7 vez para IM entre as usuárias dos AHCO.^200^

Estudo específico sobre o risco de tromboembolismo, AVC e IM em usuárias de AHCO com EE < 30 mcg constatou que, para a mesma dose de EE, o risco de TEV foi maior com o desogestrel-3^a^ geração e o gestodene-3^a^ geração, em comparação ao levonorgestrel. Esse resultado não foi observado para tromboembolismo arterial. Além disso, para o mesmo tipo de progestagênio, a dose de EE 20 mcg *versus* EE 30-40 mcg foi associada a menor risco de eventos.^201^

A associação entre HAS e uso de AHCO foi estudada na meta-análise contendo 24 estudos com 270.284 participantes que mostrou RR de 1,47 (IC 95%, 1,25-1,73) para usuárias de AHCO e uma relação dose-resposta linear de aumento de 13% (RR 1,13; IC 95%, 1,03-1,25) de casos "novos" de HAS para cada cinco anos de uso dos AHCO.^202,203^

A meta-análise sobre a relação entre AVC e uso de AHCO revelou riscos de acordo com as seguintes variáveis: 1ª) dose de EE: a cada acréscimo de 10 mcg, houve aumento na OR de 1,19 (IC 95%, 1,16-1,23) para AVC; 2ª) duração do uso do AHCO: a cada 5 anos de uso de AHCO houve aumento na OR de 1,20 (IC 95%, 1,05-1,37); e 3ª) tempo após suspensão do AHCO: para cada 5 anos após a interrupção do uso, a OR foi de 0,82 (IC 95%, 0,68-0,98), reforçando a correlação já conhecida entre estrogênio e RCV.^204^

Com relação aos contraceptivos com progestagênios isolados, a revisão sistemática e meta-análise mostrou que os RR agrupados para IM e AVC foram 0,98 (IC 95%, 0,66-1,47) e 1,02 (IC 95%, 0,72-1,44), respectivamente.^205^

As evidências indicam risco mais elevado de TEV em usuárias de AHCO com doses altas de EE (>50 mcg) em comparação às formulações com doses baixas (<35 μg). Há evidências razoáveis que usuárias de AHCO de 3ª geração têm um risco ligeiramente maior de TEV do que usuárias de AHCO de 2ª geração. A administração do combinado hormonal, em adesivo ou anel vaginal, não teve resultados diferentes para o risco de TEV quando comparada à oral.^206-208^

Em relação aos progestagênios isolados, a análise estratificada de acordo com a via de administração indicou que a via injetável apresentou risco maior de TEV (RR 2,62; IC 95%, 1,74-3,94), não observado para a oral (RR 1,06; IC 95%, 0,7-1,62), e risco diminuído para dispositivo intrauterino hormonal de levonorgestrel (DIUH) (RR 0,53; IC 95%, 0,32-0,89). Não foi encontrado efeito do uso de progestagênio isolado na PA, mas houve tendência de risco aumentado de DM para a forma injetável.^209^

#### 6.3.3. Situações Subjacentes de Risco Cardiovascular e Uso dos Contraceptivos

Há evidências que tabagismo, idade acima de 35 anos, obesidade, presença de trombofilias hereditárias, tais como mutações do fator V de Leiden e protrombina, G20210A, e deficiências das proteínas C, S ou antitrombina já apresentam riscos intrínsecos para eventos trombóticos, contraindicando os AHCO.^206,210^

Publicações sobre uso de contraceptivos hormonais em mulheres com cardiopatias são limitadas. Um estudo prospectivo ao longo de 39 meses em mulheres com lesões cardíacas estruturais (reumática e congênitas) observou 11,5% de eventos CV (HAS e isquemia transitória) nas usuárias de AHCO com EE 30 mcg + desogestrel e 7,4% nas usuárias de depo-medroxiprogesterona injetável trimestral.^211^

Enfim, a prescrição da contracepção hormonal nas situações que apresentam riscos implícitos para DCV deve seguir a orientação dos Critérios de Elegibilidade da OMS^212^ (Tabela 6.1).

**Tabela 6.1:** Critérios de elegibilidade segundo a OMS para uso dos contraceptivos hormonais em portadoras de doenças cardiovasculares (DCV)

CONDIÇÃO	AHCO PATCH ANEL	INJETÁVEL COMBINADO	POP		INJETÁVEL DMPG	IMPLANTE SUBDÉRMICO		DIUH	
**TABAGISMO**									
< 15 CIGARROS/DIA	3	2	1		1	1		1	
≥ 15 CIGARROS/DIA	4	3	1		1	1			
**MÚLTIPLOS FATORES DE RISCO PARA DCV**	3/4	3/4	2		3	2		2	
e.x. idade avançada, tabagismo, diabetes, hipertensão e dislipidemias									
**HIPERTENSÃO**	3	3	2		2	2		2	
História de hipertensão/pressão arterial não pode ser avaliada (inclui hipertensão gestacional)									
Hipertensão arterial controlada Quando a pressão arterial pode ser avaliada	3	3	1		2	1		1	
Pressão arterial elevada									
- PAS 140-159 mmHg ou PAD 90-99 mmHg	3	3	1		2	1		1	
- PAS > 160 ou PAD > 100 mmHg	4	4	2		3	2		2	
**DOENÇA VASCULAR**	4	4	2		3	2		2	
TVP / EP									
História de TVP/EP	4	4	2		2	2		2	
TEV/EP aguda	4	4	3		3	3		3	
TEV/EP em terapia anticoagulante	4	4	2		2	2		2	
Cirurgia de grande porte com imobilização prolongada	4	4	2		2	2		2	
**MUTAÇÕES TROMBOGÊNICAS RECONHECIDAS**	4	4	2		2	2		2	
Fator V Leiden, mutação da protrombina, proteína S, proteína C e deficiência antitrombina									
**DOENÇA ISQUÊMICA CARDÍACA**	4	4	I	C	3	I	C	I	C
Atual e história			2	3		2	3	2	3
**ACIDENTE VASCULAR CEREBRAL**	4	4	I	C	3	I	C	2	
Atual e história			2	3		2	3		
**DOENÇA VALVAR CARDÍACA**	4	4	1		1	1		2	
Fatores complicadores das valvopatias (hipertensão pulmonar, risco de fibrilação atrial, história de endocardite infecciosa)									

AHCO: anticoncepcional hormonal combinado oral; DCV: doença cardiovascular; patch: patch transdérmico; anel: anel vaginal; POP: pílulas orais de progesterona; DMPG: depo-medroxiprogesterona; DIUH: dispositivo intrauterino com levonorgestrel; TVP: trombose venosa profunda; EP: embolia pulmonar; PAD: pressão arterial diastólica; PAS: pressão arterial sistólica; TEV: tromboembolismo venoso; C: uso contínuo; Categoria 1: Condição sem restrição ao uso do método; Categoria 2: Condição em que as vantagens superam as desvantagens do uso do método; Categoria 3: Condição em que os riscos superam as vantagens do método; Categoria 4: Condição com inaceitável risco à saúde pelo uso do método.

#### 6.3.4. Doença Cardiovascular Tardia e Uso de Contraceptivos Hormonais

O estudo prospectivo de 11,8 anos que incluiu 161.017 mulheres mostrou que o histórico de uso dos AHCO, independentemente dos FR tradicionais, foi associado a menor risco de morte por todas as causas e de incidência de eventos CV tardios, como doença coronariana, IM, insuficiência cardíaca e FA, após ajuste para idade (todos com significância estatística p<0,05).^212-214^

Admite-se que o tratamento com os AHCO dos distúrbios reprodutivos na idade fértil exerce efeito protetor do sistema CV. Essa concepção vem ao encontro de diversas evidências que mostram que ciclos anovulatórios causados por hipoestrogenismo e disfunção hipotalâmica aumentam o risco de aterosclerose coronariana e eventos CV.^215^

### 6.4. Síndrome do Ovário Policístico

A infertilidade é uma condição que acomete 9-18% da população em idade reprodutiva e é reconhecidamente um FRCV para mulheres na faixa etária do climatério. Uma de suas causas mais comuns é a SOP que pode causar a elevação dos hormônios androgênicos e SM.^216^ A SOP afeta 6-10% das mulheres em idade reprodutiva globalmente e é definida como a presença de dois dos seguintes critérios: excesso de androgênios, presença de cistos ovarianos e oligoanovulação.^217^

As mulheres portadoras de SOP têm risco aumentado de distúrbios metabólicos reconhecidamente relacionados à aterosclerose e DCV, como obesidade, HAS, intolerância à glicose, DLP e apneia obstrutiva do sono. A DLP é a anormalidade metabólica mais frequente na SOP e, em geral, apresenta-se com baixos níveis de HDL-c e altas concentrações de TG, podendo também cursar com aumento do LDL-c.^218^

Os mecanismos biológicos que ligam a SOP ao aumento do RCV são multifatoriais. A resistência à insulina, altamente prevalente em mulheres com SOP, tanto magras quanto obesas, aumenta a lipólise do tecido adiposo, levando a DLP e vasoconstrição, que é mediada pela redução da produção de NO no endotélio vascular. A hiperinsulinemia pode levar à elevação da atividade simpática com consequente aumento da retenção renal de água e da PA, enquanto os defeitos na secreção e na ação periférica da insulina contribuem para um risco aumentado de DM.^219^

A relação de SOP com doença aterosclerótica já foi demonstrada em 2014 no estudo CARDIA (*Coronary Artery Risk Development in Young Adults*)^220^ que avaliou a presença de hiperandrogenismo e anovulação e correlacionou com o desenvolvimento de calcificações de artérias coronárias e aumento da EMI carotídea, classificando essas mulheres como portadoras de doença aterosclerótica subclínica (OR 2,70; IC 95%, 1,31-5,60).

Em uma meta-análise mais recente com 10 estudos e 166.682 mulheres incluídas, houve um aumento do risco de IM, DIC e AVC (OR 1,66; IC 95%, 1,32-2,08), mas não de mortalidade CV ou por todas as causas nas pacientes portadoras de SOP.^221^

Uma meta-análise de 32 estudos mostrou que mulheres com SOP tiveram risco 1,3 vez maior de desenvolver um composto de DCV e esse risco aumentado foi mantido quando avaliados DIC e AVC em separado, mas, novamente, não se comprovou aumento da mortalidade CV.^222^

Para mulheres que se encontram no climatério, é necessário que seus médicos conheçam seu passado de fertilidade, porque a presença de SOP pode modificar seu RCV. Esse dado deve constar na avaliação dos FRCV, assim como os antecedentes obstétricos, menopausa precoce, depressão, doenças autoimunes, entre outros FR emergentes para uma determinação de risco precisa e individualizada.

### 6.5. Uso de Testosterona em Mulheres

Testosterona é um importante esteroide sexual que atua de forma direta como andrógeno ou indireta como precursor de estrogênio em mulheres.^223^ As DCV são a principal causa de morte no mundo e, embora mulheres sejam menos afetadas que homens durante a fase reprodutiva, o RCV aumenta na fase de TM devido à perda da proteção dos estrogênios ovarianos e circulação de andrógenos.^224^

Ainda é inconclusivo se o uso de testosterona exógena traz risco ou dano de DCV em mulheres, pois não foram realizados ensaios clínicos randomizados direcionados para avaliar esses efeitos. Dados observacionais em relação à testosterona endógena e morbimortalidade em mulheres por DCV são inconsistentes, sendo alguns positivos, outros negativos e outros sem associação, talvez por diferenças entre os desenhos dos estudos.^225^ Até recentemente, o uso de androgênios em mulheres vem sendo considerado com ceticismo. O hiperandrogenismo é típico de mulheres com hirsutismo e SOP, sendo associado a RCV aumentado e distúrbios metabólicos.

No *Women's Health Study,* observou-se que um alto índice de androgênios livres estava relacionado a aumento do risco de DIC em mulheres na pós-menopausa, embora essa associação não fosse independente de outros FRCV. Também no *Cardiovascular Health Study*, encontrou-se uma associação de altos níveis de testosterona com risco de DAC. No entanto, um estado hipoandrogênico também é prejudicial à saúde CV. Baixos níveis de androgênios estão associados a aterosclerose e DAC, além de dano em parede arterial em idosos de ambos os sexos.^226^ Foi proposto que nível baixo de testosterona sérica em mulheres é prejudicial à saúde CV, dado que a testosterona em concentrações fisiológicas tem efeitos favoráveis sobre tônus vasomotor, função endotelial e resistência vascular periférica.^227^

Um subestudo do Aspirin in Reducing Events in the Elderly (ASPREE), estudo (Sex Hormones in Older Women), realizado em mulheres australianas com idade mínima de 70 anos e que não usavam nenhum tipo de reposição hormonal ou esteroide, comparou concentrações mais baixas de hormônios sexuais *versus* mais altas e avaliou como desfechos primários os eventos CV adversos maiores e mortalidade por todas as causas. Níveis séricos de testosterona e DHEA acima do quartil mais baixo em mulheres mais velhas foram associados a um risco reduzido de um primeiro evento CV adverso maior.^228^ Lopez *et al.*,^229^ em estudo observacional, avaliaram a associação de terapia de reposição de testosterona e desfechos CV em mulheres cisgênero e população transgênero, além de determinar se essa associação varia com o *status* da menopausa. Nesse estudo, o uso de terapia de reposição de testosterona aumentou o risco de DCV, DAC e AVC entre mulheres cisgênero, mas não na população transgênero do estudo.^229^

Atualmente, apenas uma indicação para suplementação de testosterona é baseada em evidências: tratamento do transtorno do desejo sexual hipoativo em mulheres na pós-menopausa. Nesse cenário, não deve-se utilizar via oral por estar associada a efeitos adversos do metabolismo lipídico, devendo a dose prescrita resultar em níveis fisiológicos de testosterona sanguínea próximos aos da pré-menopausa.^227^

A terapia de reposição de testosterona não está indicada e nem deve ser usada para a melhora de saúde cardiometabólica ou musculoesquelética, SVM ou alterações de humor, pois não temos evidências para seu uso em mulheres na pré-menopausa. Nas mulheres na pós-menopausa, a reposição de testosterona dentro dos níveis fisiológicos pode melhorar o bem-estar geral, mas os dados são ainda inconclusivos e não temos estudos suficientes sobre o impacto dos andrógenos na saúde CV em mulheres na pós-menopausa, nem se podem ser utilizados para tratamento CV. Ainda temos uma grande lacuna do conhecimento científico em relação à terapia de reposição de testosterona, necessitando estudos robustos randomizados e com avaliações específicas para os grupos populacionais nos diversos cenários atuais.

### 6.6. Terapia Hormonal e Transexualidade

Transgênero ou incongruência de gênero descreve situacão onde o gênero individual difere do sexo atribuído ao nascimento. Cerca de 0,6-1,1% da população mundial e 2,7% dos adolescentes são transgênero. A disforia de gênero seria o desconforto entre identidade de gênero e seu sexo registrado ao nascimento. A identidade de gênero seria o cuidado afirmativo, podendo incluir terapia hormonal para afirmação de gênero (THAG) e cirurgias, além de outros procedimentos.^230^

A OMS reclassifica a incongruência de gênero do capítulo de saúde mental para um novo e estabelecido "capítulo de saúde sexual", refletindo a compreensão atual de identidade de gênero. Essa reclassificação visa diminuir estigma e facilitar cuidados de saúde afirmativos de gênero.^231^

A THAG ajuda a melhorar a disforia de gênero e promover bem-estar. É difícil avaliar os efeitos CV desses tratamentos devido à variabilidade de esquemas hormonais, escassez de estudos longitudinais centrados nos resultados CV e fatores de confusão que sabidamente aumentam RCV nessa população, como estresse psicológico, tabagismo, abuso de álcool, infecção por HIV, discriminação, pobreza, sendo alguns passíveis de intervenção. Não existe consenso sobre a comparabilidade do RCV entre um indivíduo transgênero e um cisgênero; ademais, devemos buscar problemas médicos anteriores à THAG.^232^

Intervenções afirmativas de gênero incluem supressão da puberdade, hormonioterapia e cirurgia afirmativa de gênero. Neste capítulo vamos nos ater à THAG e seus efeitos sobre FRCV.^233^

#### 6.6.1. Terapia Hormonal para Afirmação de Gênero

Terapia hormonal em indivíduo transgênero visa diminuir níveis de hormônios endógenos e manter níveis hormonais compatíveis com aqueles do gênero oposto, principalmente para obtenção de caracteres sexuais secundários do gênero desejado, amenizando os do sexo biológico. Essas mudanças visam proporcionar bem-estar físico, mental e emocional.^234^

Na Tabela 6.2, encontramos os principais hormônios envolvidos na THAG, efeitos desejados e adversos.

**Tabela 6.2:** Principais hormônios envolvidos na terapia hormonal para afirmação de gênero, efeitos desejados e adversos

HORMÔNIO	VIA DE USO	DOSE	EFEITOS DESEJADOS	EFEITOS ADVERSOS GERAIS
**MULHER TRANSGÊNERO**				
Valerato de estradiol	Oral	2-6 mg/dia	Hormônio adequado para as mudanças: suprime gonadotrofinas e, consequentemente, produção dos andrógenos Essas mudanças podem ser definitivas e devem ser esclarecidas com relação à fertilidade	Terapia com estrogênios em geral: Alterações no metabolismo hepático Alterações no colesterol têm resultados conflitantes Aumento de triglicérides Redução de homocisteína sérica Estado protrombótico dependente da dose e via de administração Maior incidência de IM vs. mulheres cis Maior incidência de IM vs. homens cis Maior incidência de TEV vs. mulheres e homens cis
Estradiol	Transdérmico	0,025-0,2 mg/dia		
Estradiol (valerado ou cipionato)	Parenteral	2-10 mg intramuscular/semana		
**ANTIANDROGÊNIOS**				
Espironolactona	Oral	100-300 mg/dia		Efeito antimineralocorticoide, podendo alterar níveis pressóricos Em doses altas: potencial aumento de meningiomas; depressão e hiperprolactinemia
Acetato de ciproterona	Oral	25 mg/dia		
Triptorrelina	Subcutâneo	3,75 mg/mês		
Finasterida	Oral			
**HOMENS TRANSGÊNEROS**				
Testosterona cipionato ou enantato	Parenteral	100-200 mg a cada 4 semanas		Terapia com testosterona no geral: Alterações adversas no metabolismo de colesterol: aumento de LDL, redução de HDL e leve aumento de triglicérides Aumento de resistência à insulina Aumento da homocisteína sérica Aumento da espessura mediointimal com uso continuado Apesar dos efeitos adversos descritos, não parece aumentar desfecho CV em homens trans na maioria das séries. Entretanto, alguns estudos demonstram aumento de IM em homens trans vs. gêneros cis no geral
Testosterona decanoato	Parenteral	1000 mg a cada 12 semanas		
Testosterona gel 1,6%	Transdérmico	50-100 mg/dia		
Testosterona Patch	Transdérmico	25-75 mg/dia		

AVC: acidente vascular cerebral; CV: cardiovascular; IM: infarto do miocárdio; TEV: tromboembolismo venoso.

##### A – Terapia transexual feminina com estrogênios

O estradiol é o hormônio adequado para as mudanças, suprimindo gonadotrofinas e, consequentemente, a produção dos andrógenos. Essas mudanças podem ser definitivas e devem ser esclarecidas com relação à fertilidade.

Etinilestradiol é um estrogênio sintético que foi amplamente utilizado. Entretanto, devido ao seu potencial pró-trombótico e o seu possível papel na DCV, a maioria dos esquemas atuais usa o valerato de estradiol oral, cutâneo ou intramuscular. Faltam estudos que comparem a segurança e a eficácia a longo prazo entre as diferentes formulações de estradiol.^230,235^

##### B – Terapia de supressão de andrógenos

Espironolactona reduz a síntese de testosterona e sua ação no receptor androgênico. Agonistas liberadores de gonadotrofinas, como triptorrelina e acetato de ciproterona, bloqueiam os receptores de androgênio. Finasterida melhora a alopecia androgenética em mulheres transgênero.^236^

##### C – Terapia transexual masculina com testosteronas

O principal tratamento hormonal é a testosterona, com objetivo de parar a menstruação e induzir virilização, alteração de voz e aquisição de contornos físicos masculinos. Essas mudanças podem ser definitivas e devem ser esclarecidas com relação à fertilidade. Deve-se monitorizar peso, níveis de PA e valor do hematócrito, pois eritrocitose pode ocorrer com o uso de alguns tipos de testosterona.^231,237^

#### 6.6.2. Transexualidade e Saúde Cardiovascular

Em toda a literatura referente a indivíduos transgênero e RCV, observa-se preocupação com os FR confundidores presentes em quase 50% dessa população: tabagismo, estresse, alterações do sono e, especialmente, os determinantes psicossociais. Temos de salientar que, muitas vezes, a THAG se inicia na adolescência, aumentando o tempo de exposição aos hormônios e FR psicossociais nessa população. Assim, não só o cardiologista, mas toda a equipe multidisciplinar envolvida nesses cenários deve procurar abordar e intervir positivamente nesses pilares da saúde CV que são formados de variáveis qualitativas de difícil análise e avaliação de impacto, mas de grande importância. As lacunas do conhecimento nesse tema são amplas, necessitando futuras pesquisas bem conduzidas.^181,231^

## 7. Recomendações Atuais para Terapia Hormonal da Menopausa

Nos últimos 20 anos, estudos longitudinais com mulheres na transição para a menopausa enfatizaram o aumento do risco de DCV nesse período. Esse aumento do risco decorre de alterações endógenas dos hormônios sexuais e mudanças desfavoráveis na distribuição de gordura corporal, lipídios e lipoproteínas, bem como nas medidas funcionais da saúde vascular na TM, descritas nos capítulos anteriores. Esses dados enfatizam a importância da monitorização para se intervir na saúde CV das mulheres na TM.^238^

Por esses motivos, a partir do final da década de 1980, houve uma mudança da prescrição de terapia de estrogênio de curto prazo para os sintomas das mulheres na TM, optando-se pela prescrição de longo prazo (mais de 5 anos) para prevenção de DCV, especialmente DAC. Essa estratégia de prevenção foi baseada em mais de 30 estudos observacionais, quase todos demonstrando um efeito protetor do estrogênio em relação às DCV.^238^ Além dos dados observacionais, estudos angiográficos e de autópsia sugeriram um efeito antiaterogênico do estrogênio.^239,240^

Embora os estudos observacionais iniciais sugerissem benefícios da THM para a prevenção primária e secundária da DCV, isso não foi confirmado em grandes ensaios clínicos subsequentes. O estudo WHI foi um conjunto de ensaios clínicos, incluindo dois ensaios de terapia com estrogênio, em mulheres saudáveis na pós-menopausa com idades entre 50 anos e 79 anos. Um dos ensaios que testou o regime combinado contínuo de estrogênio-progestagênio [estrogênio conjugado 0,625 mg e acetato de medroxiprogesterona 2,5 mg/dia] *versus* placebo em mais de 16 mil mulheres foi descontinuado devido a um risco aumentado de câncer de mama, AVC, DAC e TEV durante um acompanhamento médio de 5,2 anos.^1^ Outro estudo com estrogênio conjugado 0,625 mg *versus* placebo em cerca de 11 mil mulheres submetidas a histerectomia também foi descontinuado, devido a risco aumentado de AVC e ausência de benefício para a saúde CV das mulheres na menopausa.^241^

Fatores incluindo a idade avançada dos participantes da população do WHI, bem como o tipo de estrogênio, a via de administração e a dose empregada foram hipóteses elencadas para explicar os resultados discordantes.^242^ Outros fatores propostos para os resultados adversos observados foram IMC elevado, comorbidades associadas, como diabetes, história familiar e tabagismo, e o tempo de TM até o início da THM. Estudos subsequentes demonstraram que o risco absoluto de qualquer evento adverso (câncer de mama, DAC, AVC ou TEV) foi baixo, 19 eventos adicionais por ano por 10 mil mulheres em uso de THM *versus* placebo.^243^

Sabidamente, o estrogênio é o tratamento mais eficaz disponível para o alívio dos sintomas da menopausa, principalmente os SVM. A THM, estrogênio isolado ou combinado com progestagênio, é atualmente indicada para o tratamento dos sintomas da menopausa. O uso a longo prazo para prevenção de doenças não é mais recomendado.^244^

Dados de um modelo de primata, estudos observacionais em mulheres na pós-menopausa, meta-análise de ensaios clínicos, estudo de angiografia coronariana e análises secundárias do WHI sugerem que o momento da exposição à THM é um fator importante na influência do RCV subsequente. O uso de THM nos primeiros anos da menopausa não parece estar associado a um aumento do risco de DCV quando comparado ao de mulheres mais velhas na pós-menopausa.^238^ A população do estudo WHI era mais velha (média de idade, 63 anos) do que a da maioria dos estudos observacionais. A idade mais avançada no momento do início da THM poderia estar associada com mais aterosclerose subclínica no início do estudo, com lesões ateroscleróticas avançadas ou complexas que podem ser mais suscetíveis aos efeitos pró-trombóticos e pró-inflamatórios do estrogênio, em especial quando utilizado pela via oral. Em contraste, iniciar a THM logo após a menopausa pode não causar danos (ou possivelmente ser benéfico).

Uma meta-análise de 19 ensaios de THM oral (incluindo o WHI) com mais de 40 mil mulheres na pós-menopausa, com análises de subgrupos em mulheres que iniciaram a THM menos de 10 anos após a menopausa, reportou menor risco de DAC e mortalidade por DCV.^245^ Uma meta-análise de ensaios semelhantes de 2017 concluiu que os riscos do uso prolongado da THM para prevenção de doenças crônicas superavam quaisquer benefícios.^246^

Devido ao aumento da expectativa de vida das mulheres, estima-se que 40% do seu tempo de vida transcorrerá no período da pós-menopausa. Considerando-se que:

- Os SVM estão associados a pior perfil cardiometabólico e medidas de aterosclerose subclínica.
- Os distúrbios do sono são uma queixa comum durante a TM e estão associados a um maior risco de DCV subclínica e piores índices de saúde CV.
- A depressão ocorre com maior frequência durante os anos de perimenopausa e pós-menopausa e está relacionada com SVM e ocorrência de DCV.
- O aumento da adiposidade central que ocorre na TM está associado a risco aumentado de mortalidade, mesmo entre aqueles com IMC normal.
- Os aumentos de lipídios (LDL-c e apolipoproteína B), o risco de SM e o remodelamento o vascular são impulsionados mais pela TM do que pelo envelhecimento.
- A ocorrência de maior risco de fraturas por perda de massa óssea associada à TM.

A SBC, a FEBRASGO e a SOBRAC recomendam a **FAVOR** da adoção da THM para as mulheres climatéricas sintomáticas sem contraindicações. (**Força da recomendação a FAVOR. Recomendação FORTE. Nível de certeza ALTO**).^64,94,132,238,242,247-254^

Essa terapia consiste na administração de hormônios sexuais que devem ser individualizados de acordo com os riscos e benefícios de cada mulher. As várias formulações, doses e vias de administração de THM têm alta eficácia no alívio de sintomas do climatério e foram discutidas nos capítulos anteriores. (**Força da recomendação a FAVOR. Recomendação FORTE. Nível de certeza ALTO**). Não há idade ou duração máxima pré-estabelecida para uso da THM, devendo a decisão de continuar ou interromper a THM ser baseada na manutenção das indicações e na ausência de mudanças de riscos. (**Força da recomendação a FAVOR. Recomendação FORTE. Nível de certeza MODERADO**).^64,179,244,247,255-259^

A indicação primária de THM sistêmica é o tratamento dos SVM, sendo essa terapia a mais efetiva e considerada padrão-ouro para alívio desses sintomas. A terapia estrogênica vaginal isolada é efetiva somente para tratar os sintomas da síndrome geniturinária. A THM também está indicada para prevenção de perda de massa óssea e redução do risco de fraturas. Para mulheres com IOP, a THM deve ser usada no mínimo até próximo aos 50 anos, idade média de ocorrência da menopausa. (**Força da recomendação a FAVOR. Recomendação FORTE. Nível de certeza ALTO**).^34,143,260-273^

A literatura que apoia um papel crítico para o tempo de início do uso de THM antes dos 60 anos de idade ou dentro de 10 anos após a menopausa, parece ser associada à redução do risco de DCV. As evidências sugerem que os efeitos da THM na progressão de eventos de aterosclerose e DCV variam de acordo com a idade quando a THM é iniciada ou o tempo desde a menopausa até o início da THM. Os efeitos benéficos nos resultados de DCV e mortalidade por todas as causas podem ocorrer quando a THM é iniciada em mulheres < 60 anos de idade ou < 10 anos desde a menopausa, porém podem ocorrer efeitos nulos ou prejudiciais quando a THM é iniciada em idades mais avançadas ou após maior tempo desde a menopausa.

Numerosos estudos observacionais antes de 1991, que preconizavam o uso de THM próximo ao início da menopausa, reportaram redução das taxas de DAC em usuárias de THM.^273^ Análise dos estudos observacionais prospectivos relatou um RR de eventos de DAC de 0,50 (IC 95%, 0,43-0,56).^42^ Análises do *Nurses’ Health Study,* com mulheres de 30-55 anos de idade no início do estudo, mostraram menor risco de mortalidade entre as usuárias atuais em comparação àquelas que nunca usaram THM (RR, 0,63; IC 95%, 0,56-0,70), sendo a redução da mortalidade maior naquelas com maior risco.^274^ Estudos caso-controle e transversais também mostraram redução da mortalidade em mulheres em THM com DAC definida angiograficamente.^273,275-277^

A THM deve ser iniciada na "janela de oportunidade", isso é, nos primeiros 10 anos após o início da menopausa e/ou antes dos 60 anos de idade.** A decisão de iniciar a THM, a dose utilizada, o regime e a duração do seu uso devem ser feitos de forma individualizada após discutir os benefícios e riscos com cada paciente e após fornecer à paciente folheto informativo do produto selecionado. Isso deve ser considerado no contexto dos benefícios globais obtidos com o uso de THM, incluindo controle de sintomas e melhora da qualidade de vida, bem como considerando os potenciais benefícios CV e ósseos associados ao uso da THM. (**Força da recomendação a FAVOR. Recomendação FORTE. Nível de certeza ALTO**).

Não se recomenda o uso combinado de estrogênio e progestagênio para a prevenção primária de condições crônicas em pessoas assintomáticas na pós-menopausa. Também não se recomenda o uso de estrogênio isolado para a prevenção primária de condições crônicas em pessoas na pós-menopausa submetidas a histerectomia. Os efeitos da THM no risco de DCV variam dependendo do momento em que é iniciada. Em mulheres saudáveis, quando iniciada na janela de oportunidade, pode apresentar efeitos favoráveis no risco de DCV. Entretanto, não há indicação de se iniciar a THM com o objetivo de prevenção primária CV nos múltiplos cenários. Contrariamente, iniciar a THM após os 60 anos de idade ou mais de 10 anos após a menopausa pode elevar o risco absoluto de DAC, TEV e AVC. (**Força da recomendação a FAVOR. Recomendação FORTE. Nível de certeza ALTO**).^29,42,255,283-290^

Com a cessação da função ovariana, ocorre grande impacto sobre os FR cardiometabólicos, onde se incluem o aumento da PA, do colesterol, da massa corporal e da glicose plasmática. A obesidade está fortemente relacionada à elevação da PA em mulheres.^291,292^ Na menopausa, em resposta à elevação da PA, a mulher apresenta anormalidades mais intensas da microcirculação, maior ocorrência de doença renal crônica e de alterações na microcirculação coronariana, bem como de hipertrofia ventricular esquerda concêntrica.^291,292^ A HAS na mulher é um FR mais forte para infarto agudo do miocárdio (IAM), insuficiência cardíaca com fração de ejeção preservada (mais comum em mulheres) e reduzida, doença arterial periférica, AVC e declínio cognitivo. Na menopausa mais tardia, também foi observada aceleração significativa da rigidez arterial, o que pode contribuir para um risco CV ainda maior.^293^

Doses de reposição de estrogênio têm pouco efeito sobre a PA. O ensaio combinado de estrogênio-progestagênio WHI observou apenas um pequeno aumento (1,5 mmHg) na PA sistólica em comparação com placebo.^1^ Além disso, uma diferença semelhante entre os grupos em uso de hormônio e placebo de 1,1 mmHg foi observada no estudo WHI de estrogênio isolado.^241^ Da mesma forma, o estudo Postmenopausal Estrogen/Progestin Interventions (PEPI) reportou que o estrogênio, isolado ou combinado com progestagênio, não afetou a PA. Esses achados contrastam com a elevação frequente da PA observada quando doses mais altas de estrogênio sintético (EE) são administradas como contracepção oral.291 Mulheres com HAS controlada e SVM moderados a intensos podem utilizar THM por qualquer via, sendo, porém, preferível a terapia estrogênica transdérmica (através de gel ou adesivos) na presença de obesidade, DLP, DM e SM. (**Força da recomendação a FAVOR. Recomendação FORTE. Nível de certeza MODERADO**).

Todas as mulheres na peri- e pós-menopausa, incluindo aquelas que usam THM devem realizar mamografia de rastreamento periódico de acordo com as diretrizes atuais de rastreamento. A FEBRASGO sugere que antes da prescrição da THM seja realizada mamografia. Adicionalmente, a quantificação de CT e suas frações, TG e glicemia de jejum auxilia na escolha da melhor via de administração da THM. Outros exames complementares podem ser necessários a depender dos achados na anamnese e no exame físico e dos FR. Além disso, mulheres climatéricas com estratificação de risco intermediário para DCV podem necessitar uma avaliação complementar para melhor individualizar a THM.179,294

Evidências observacionais sugeriram que poderia haver um efeito protetor da THM sobre as DCV. No entanto, o WHI e outros estudos não corroboraram esses achados. A análise de 3 ensaios (n = 18.085) não mostrou diferença significativa em risco de eventos de DAC em pessoas tratadas com estrogênio associado a progestagênio em comparação com placebo (2,8% *versus* 2,6%; RR, 1,12; IC 95%, 0,94-1,33) durante um acompanhamento médio de 4 anos. Da mesma forma, uma análise agrupada de 3 ensaios (n = 11.310) não encontrou nenhuma diferença significativa em eventos coronários entre pessoas que usaram estrogênio isolado e aquelas que usaram placebo (RR, 0,95; IC 95%, 0,79-1,14) durante um seguimento médio de 4,1 anos. O WHI reportou um risco aumentado de AVC com estrogênio tanto em terapia isolada quanto em terapia combinada com progestagênio. O risco de AVC foi significativamente maior em pessoas randomizadas para receber estrogênio associado a progestagênio em comparação com aquelas randomizadas para placebo (1,9% *versus* 1,3%; HR, 1,37; IC 95%, 1,07-1,76). Igualmente, as mulheres que receberam apenas estrogênio tiveram um risco estatisticamente maior de AVC em comparação com aquelas que receberam placebo (3,2% *versus* 2,4%; HR, 1,35; IC 95%, 1,07-1,70). Não se recomenda a THM sistêmica em mulheres com DCV manifesta, histórico prévio de IAM ou AVC.^279^ (**Força da recomendação a FAVOR. Recomendação FORTE. Nível de certeza ALTO**).^283,295^ A terapia estrogênica por via vaginal para o tratamento da síndrome geniturinária da menopausa pode ser utilizada em pacientes com FRCV conhecidos ou DCV estabelecida e não necessita o acréscimo de progestagênio naquelas histerectomizadas.^29,42,179,255,284-290,294^

Para mulheres com contraindicação ou que não desejam usar THM, terapias não hormonais com eficácia comprovada*** podem melhorar os SVM. (**Força da recomendação a FAVOR. Recomendação FORTE. Nível de certeza ALTO**).

Cinco ensaios relataram risco de tromboembolismo com o uso da THM oral. No WHI (n = 16.608), mulheres randomizadas para receber estrogênio conjugado combinado com medroxiprogesterona tiveram um risco aumentado de trombose venosa (1,96% versus 0,94%; HR, 2,06; IC 95%, 1,57-2,70), TVP (1,4% versus 0,8%; HR, 1,87; IC 95%, 1,37-2,54) e EP (1,0% versus 0,5%; HR, 1,98; IC 95%, 1,36-2,87) em comparação com aquelas no grupo placebo.132,298 Outros estudos relataram poucos eventos tromboembólicos ou eram consistentes com os resultados do WHI.283,295 No WHI (n = 10.739), mulheres randomizadas para receber apenas estrogênio tiveram um risco aumentado de TVP (1,6% versus 1,0%; HR, 1,48; IC 95%, 1,06-2,07). O risco de EP foi maior no grupo estrogênio do que no grupo placebo, mas os resultados não foram estatisticamente significativos, embora o IC tenha sido amplo (0,98% versus 0,72%; HR, 1,35; IC 95%, 0,89-2,05).132 Para mulheres com histórico de TEV, a THM em geral não é recomendada, mas, a depender do fator que ocasionou o evento, se a decisão for pela indicação, a utilização da via transdérmica seria a de menor risco. (**Força da recomendação a FAVOR. Recomendação FORTE. Nível de certeza MODERADO**). Doença hepática descompensada, sangramento de causa desconhecida e lúpus eritematoso sistêmico com elevado risco trombótico também são contraindicações com nível de evidência fraca. **(Força da recomendação a FAVOR. Recomendação FRACA. Nível de certeza BAIXO**).

Os ensaios da THM relataram o risco de câncer de mama como um dos principais resultados adversos do tratamento. No WHI, foram randomizadas 16.608 mulheres para receber estrogênio combinado com progestagênio ou placebo. Houve aumento significativo do risco de câncer de mama no grupo que recebeu a THM em comparação com aquelas que receberam placebo (2,4% *versus* 1,9%; HR, 1,24; IC 95%, 1,01-1,53),^132^ tendo o risco persistido no seguimento.^303-305^ Também no WHI, durante 20,3 anos de acompanhamento, a estimativa pontual do risco de mortalidade por câncer de mama foi maior para pessoas no grupo estrogênio combinado com medroxiprogesterona do que para aquelas no grupo placebo, embora a diferença não tenha alcançado significância estatística (HR, 1,35; IC 95%, 0,94-1,95).^304^ Quatro estudos relataram os efeitos do estrogênio isolado sobre câncer de mama; no entanto, apenas o WHI acompanhou as participantes por mais de 3 anos. Com 20,7 anos de seguimento, o WHI relatou um risco menor de câncer de mama invasivo no grupo que recebeu estrogênio isolado em comparação com as mulheres que receberam placebo (HR, 0,78; IC 95%, 0,65-0,93).^1^ Ainda no estudo WHI, no acompanhamento de 20,7 anos, o grupo de intervenção com estrogênio teve um risco menor de mortalidade por câncer de mama do que no grupo placebo (HR, 0,60; 95% IC, 0,37-0,97).^304^ O risco de câncer de mama associado à THM é baixo, com menos de um caso adicional por 1.000 mulheres por ano de uso. (**Força da recomendação a FAVOR. Recomendação FORTE. Nível de certeza ALTO**).

Em 16.608 mulheres, a THM foi associada com um pequeno aumento no risco de câncer de mama com o uso de 0,625 mg/d de estrogênio equino conjugado mais 2,5 mg/d de acetato de medroxiprogesterona comparado ao placebo (0,38%/ano para estrogênio equino conjugado mais acetato de medroxiprogesterona *versus* 0,30%/ano para placebo).^1^ Em 10.738 mulheres, o tratamento com 0,625 mg/d de estrogênio equino conjugado isolado não mostrou esse desfecho (0,26%/ano para estrogênio equino conjugado isolado *versus* 0,33%/ano para placebo).^306^ São contraindicações à THM as neoplasias hormônio-dependentes, como câncer de mama, lesões precursoras para câncer de mama, câncer de endométrio, antecedente pessoal de DAC e cerebrovascular. (**Força da recomendação a FAVOR. Recomendação FORTE. Nível de certeza ALTO**).

Hormônios naturais constituídos por preparações de estradiol e progesterona micronizada são aprovados pela agência americana FDA e são disponíveis mediante prescrição. Em contrapartida, preparações manipuladas de hormônios bioidênticos não são aprovadas por essa agência reguladora e devem ser evitadas porque não foram avaliadas quanto à segurança ou eficácia e não são monitoradas quanto à qualidade.307 Em 2020, um relatório das Academias Nacionais de Ciências, Engenharia e Medicina concluiu que as preparações de hormônios bioidênticos manipulados não incluíam rotulagem adequada sobre instruções para uso, contraindicações e potenciais efeitos adversos e careciam de informações confiáveis sobre os dados para avaliar segurança, eficácia e relação da variabilidade produto a produto.306,307 A maioria das afirmações de marketing sobre a segurança e a eficácia de preparações manipuladas de hormônios bioidênticos não é embasada em estudos devidamente controlados. O uso de preparações de hormônios bioidênticos (por exemplo, desidroepiandrosterona, estradiol, cipionato de estradiol, estriol, estrona, pregnenolona, progesterona, testosterona, testosterona cipionato e propionato de testosterona) deve ser restrito a pessoas com alergia documentada a ingredientes farmacêuticos ativos ou excipientes de medicamentos aprovados pelas agências reguladoras. As pacientes devem ser informadas sobre os riscos inerentes à falta de regulamentação para preparações manipuladas de hormônios bioidênticos. Os implantes hormonais manipulados ou outros hormônios "bioidênticos" "manipulados" e a chamada "modulação hormonal" não são recomendados pela falta de evidência científica de eficácia e segurança desses compostos.306,307 A SBC, a FEBRASGO e a SOBRAC **posicionam-se CONTRA a adoção dessas terapias. Recomendação FORTE. Nível de certeza MUITO BAIXO**.

As pacientes devem ser informadas que acupuntura, terapia de relaxamento, fitoestrógenos, exercícios e *black cohosh* não são significativamente melhores que placebo para aliviar os SVM.^308-311^ Em ensaios clínicos de terapias para tratar SVM, até 50% dos participantes responderam ao placebo.^312^ Portanto, um controle com grupo placebo é necessário para avaliar a eficácia de potenciais tratamentos para SVM.^306^

Aumento absoluto no risco associado à THM são menores para mulheres que começam o tratamento próximo ao início da menopausa. Maior segurança das preparações transdérmicas de estradiol em relação a estrogênio equino conjugado combinado com acetato de medroxiprogesterona via oral (em relação ao risco de AVC ou DAC) é sugerida por grandes estudos observacionais, não existindo ensaios clínicos randomizados, controlados e multicêntricos.^306^ Importante ressaltar que são necessários novos ensaios clínicos que abordem questões ainda não respondidas, como por exemplo: a idade ou o momento de início da THM afeta diferentemente os resultados de saúde?; os benefícios e malefícios da THM podem variar entre grupos populacionais, raciais e étnicos?; as barreiras socioeconômicas que implicam em maior risco de certas condições crônicas (DM, AVC, DAC crônica) alteram os resultados da THM?; há benefícios e malefícios comparativos de diferentes formulações e durações do tratamento da THM?^255^

**Figura 7.1:**
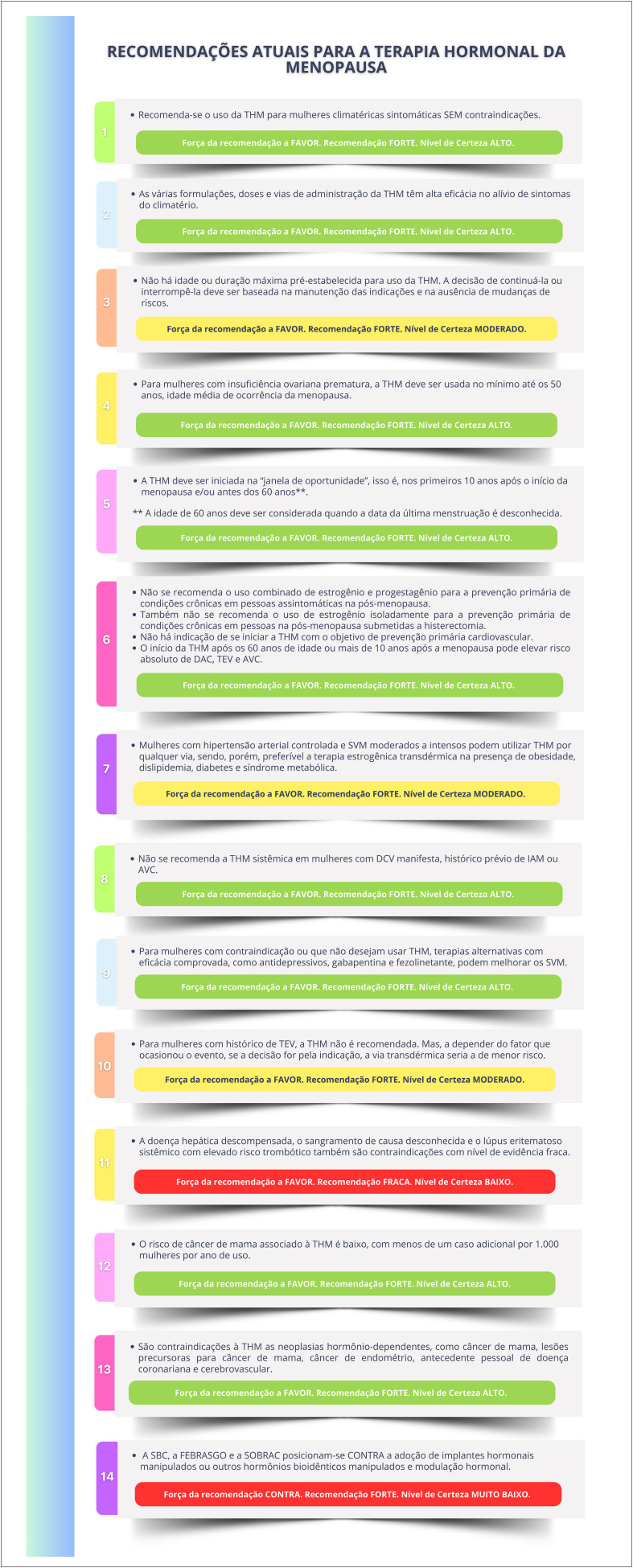
(continuação) – Recomendações atuais para a terapia hormonal da menopausa. AVC: acidente vascular cerebral; DAC: doença arterial coronariana; DCV: doença cardiovascular; FEBRASGO: Federação Brasileira das Associações de Ginecologia e Obstetrícia; IAM: infarto agudo do miocárdio; SBC: Sociedade Brasileira de Cardiologia; SOBRAC: Associação Brasileira de Climatério; SVM: sintomas vasomotores; TEV: tromboelismo venoso; THM: terapia hormonal da menopausa.

As recomendações atuais para a THM são sumarizadas na Figura 7.1.

## 8. Evidências Contemporâneas da Terapia Hormonal em Mulheres

### 8.1. O que Deve Ser Utilizado: Tipos, Doses e Vias de Administração

A THM consiste na administração farmacológica de esteroides sexuais, especialmente estrogênios e progestagênios, a mulheres na TM ou na pós-menopausa com a finalidade de aliviar sintomas ou prevenir problemas de saúde.^313^

A *International Menopause Society* (IMS) alerta para que se evite o termo "efeito de classe", haja vista o grande número de possibilidades devido a diferentes hormônios, vias de administração, doses e regimes.^313^

O esteroide mais importante na THM é o estrogênio, por ser o responsável pelo alívio dos sintomas climatéricos e pela proteção contra a osteoporose. O papel do progestagênio é impedir a proliferação endometrial propiciada pelo estrogênio, evitando assim o aumento do risco de câncer de endométrio.^179^

Os estrogênios mais empregados em THM são o estradiol, idêntico ao endógeno do ponto de vista molecular, e os estrogênios conjugados. O estradiol está disponível sob a forma de 17-beta-estradiol ou como valerato de estradiol.^179,314^ Outra formulação utilizada na THM é a tibolona, um progestagênio sintético, apesar de seus metabólitos apresentarem ações estrogênica, androgênica e progestacional.^315^

Quanto aos progestagênios, os mais frequentemente empregados são os sintéticos: noretisterona, didrogesterona, drospirenona, acetato de nomegestrol, acetato de medroxiprogesterona, progesterona micronizada (idêntica à progesterona natural), entre outros.^179^

Quando se considera a via de administração da THM, a questão central refere-se ao estrogênio, podendo a via ser oral, transdérmica ou vaginal.^179^ A via vaginal destina-se a efeitos locais no sistema genital, sem efeitos sistêmicos significativos,^316^ enquanto as vias oral e transdérmica conferem efeitos sistêmicos.^179^

Ao ser absorvido pelo tubo digestivo, o estrogênio vai ao fígado pelo sistema porta, exercendo efeitos na síntese proteica hepática (por exemplo, dos fatores de coagulação) e, apenas após essa passagem hepática, finalmente chega à circulação sistêmica. Quando o estrogênio é administrado por via transdérmica, é distribuído para a circulação sistêmica tão logo absorvido, chegando ao fígado apenas posteriormente. Por isso, a administração oral do estrogênio tem maior impacto hepático do que a via transdérmica e, a esse fenômeno, convencionou-se chamar de efeito de primeira passagem hepática.^317^

Esse fenômeno explica o fato de o estrogênio oral favorecer algum incremento dos níveis plasmáticos de TG, mas também propicia maior acréscimo do HDL-c associado a maior redução do LDL-c do que o observado com a via transdérmica.^318^

Quanto aos riscos de eventos tromboembólicos, estudos caso-controle revelaram seu aumento associado ao estrogênio oral, mas sem incremento com a via transdérmica.^319-321^ O último estudo mostrou RR de 1,40 (IC, 1,32-1,48) associado ao estrogênio por via oral, e RR de 0,96 (IC, 0,88-1,04) por via transdérmica.^321^

No que tange à dose hormonal a ser empregada, atualmente preconiza-se as menores doses efetivas, utilizando-se as maiores doses para os casos em que a resposta foi inadequada.^179,313^ Um dos estudos que fez avaliação de diferentes doses de estradiol oral observou que as doses de 2 mg, 1 mg e 0,5 mg foram superiores ao placebo no alívio dos SVM, o que não ocorreu com a dose de 0,25 mg, concluindo-se, portanto, que a menor dose eficaz de estradiol oral é 0,5 mg.^322^ O estradiol transdérmico no Brasil está disponível sob a forma de adesivos, nas doses de 25 mcg e 50 mcg, e sob a forma de gel, nas doses de 0,5 mg/dia a 3,0 mg/dia.^314^

### 8.2. Como Prescrever THM

A IMS, a *North American Menopause Society* (NAMS) e a SOBRAC concordam que o tratamento dos sintomas climatéricos, bem como a prevenção da osteoporose, são indicação primordial da THM. Para a síndrome geniturinária, há preferência por estrogênios de aplicação vaginal.^179,313,323^

**Figura 8.1:**
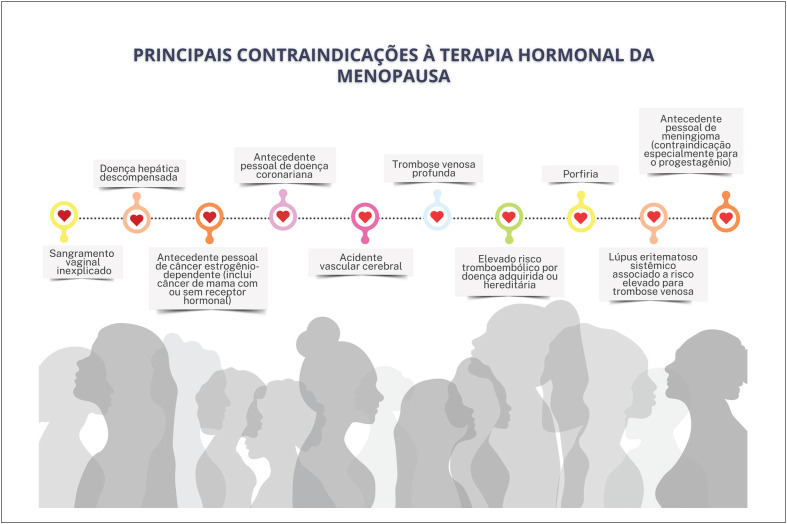
Principais contraindicações à terapia hormonal da menopausa.^179,323^

Pacientes com IOP apresentam indicação formal de THM na ausência de contraindicações.^179^ A Figura 8.1 mostra as principais contraindicações à THM.^179,323^

Antes da prescrição da THM, a SOBRAC considera essencial a realização de mamografia em um período menor que 12 meses, além da avaliação laboratorial do perfil lipídico e glicemia de jejum. Outros exames complementares podem ser necessários a depender de achados na anamnese e no exame físico. Demais exames de rastreamento ginecológico devem ser solicitados conforme as diretrizes específicas e não em decorrência da possibilidade de se iniciar a THM.^38,323^

### 8.3. Quando Prescrever e Quanto Tempo Deve Durar a THM

O WHI foi um grande estudo que randomizou mulheres na pós-menopausa para receberem THM por meio de estrogênios conjugados isolados ou combinados com acetato de medroxiprogesterona comparados aoplacebo. Esse estudo revelou que a THM se associou a maior risco de eventos CV,^1^ ao contrário do que foi reportado no estudo observacional *Nurses’ Health Study*.^324^

Entretanto, é importante fazer uma análise crítica em relação à população estudada: mais de dois terços das mulheres tinham idade acima de 60 anos no momento da randomização, sendo uma proporção relevante de mulheres acima de 70 anos. Além disso um número menor de mulheres tinha menos de 10 anos de evolução da menopausa quando começaram aTHM, sendo que a grande maioria não apresentava SVM moderados ou graves.^325^

Por outro lado, notou-se que a THM iniciada nos primeiros anos a partir da menopausa poderia diminuir a progressão da aterosclerose^281^ ou mesmo reduzir desfechos CV.^325^

Importante notar que no estudo *Nurses’ Health Study*, as mulheres que iniciaram a THM apresentavam, em média, menos tempo de pós-menopausa e eram mais sintomáticas do que as do WHI, aproximando-se mais da realidade da prática clínica diária dos médicos.^326-328^

No estudo WHI, ao se analisar os subgrupos com menos de 10 anos, entre 10 anos e 20 anos e mais de 20 anos decorridos de menopausa quando do início da THM, os autores notaram aumento de RCV com significância estatística apenas naqueles com mais de 20 anos.^329^

Assim, criou-se o conceito de janela de oportunidade para início da THM, que corresponde ao início nos primeiros anos de pós-menopausa. A NAMS recomenda o início da THM para mulheres nos primeiros 10 anos de pós-menopausa ou antes dos 60 anos de idade. Nas mulheres com indicações, respeitada a janela de oportunidade e na ausência de contraindicações, os benefícios da THM superam os eventuais riscos, sendo estas orientações corroboradas pela IMS e SOBRAC.^313,323^

Uma avaliação conjunta do tempo decorrido de pós-menopausa e RCV foi proposta por Kaunitz & Manson juntamente com a NAMS. Se o tempo decorrido desde a menopausa for menor do que 10 anos e o RCV calculado por meio de calculadoras de risco classificarem a candidata à THM como baixo risco, a THM está liberada; se o RCV for intermediário, a THM pode ser prescrita, porém, a via transdérmica para o estrogênio deve ser preferida. Caso o tempo decorrido de pós-menopausa supere 10 anos ou se o RCV estimado for alto, a THM não é recomendada. Em qualquer situação é importante avaliar as contraindicações e a anuência da paciente^35^

Em relação à duração da THM, nenhuma das mais importantes diretrizes sobre o tema estabelece uma duração máxima mandatória ou idade na qual a THM deve ser interrompida. Essa decisão deve ser realizada a cada consulta de seguimento com base na avaliação da relação risco-benefício na avaliação na avaliação individualizada do risco-benefício.^179,313,323^

### 8.4. Prescrição nos Diferentes Cenários de Risco Cardiovascular (HAS, DM, DLP)

As DCV constituem a principal causa de morte no mundo e no Brasil entre as doenças não transmissíveis, e respondem por um terço das mortes por todas as causas, acometendo homens e mulheres em todas as faixas etárias. Nas mulheres, os FR tradicionais para DCV incluem: DM, HAS, DLP, tabagismo, obesidade e sedentarismo.^13^ As indicações da THM aprovadas pelas sociedades de menopausa em todo o mundo, incluindo a brasileira (SOBRAC), abrangem o tratamento dos SVM moderados a graves, os sintomas e sinais decorrentes da síndrome geniturinária da menopausa, prevenção da perda óssea e de fraturas osteoporóticas, além do hipoestrogenismo decorrente do hipogonadismo, ooforectomia bilateral e IOP.^179,313,330,331^

A prescrição da THM pode tornar-se um desafio nas pacientes que apresentam uma ou mais morbidades ou algum dos FRCV conhecidos, e que serão abordados abaixo nos próximos parágrafos.

#### 8.4.1. Obesidade

A obesidade é considerada FR para TEV. O uso de estrogênio por via oral em pacientes com sobrepeso têm efeito aditivo no aumento do risco de TEV, como demonstrado em alguns estudos observacionais e em revisões sistemáticas publicadas.^319,332,333^ No estudo WHI, mulheres com sobrepeso e obesas randomizadas para uso de THM sistêmica apresentaram aumento do risco de TEV em 3 vezes (HR, 3,80; IC 95%, 2,08–6,94) e 6 vezes (HR, 5,61; IC 95%, 3,12–10,11), respectivamente, quando comparadas àquelas em uso de placebo.^298^ Na ausência de ensaios clínicos randomizados e pelos dados consistentes de estudos observacionais mostrando baixo risco de TEV com THM por via transdérmica, essa via deve ser a preferida para pacientes com sobrepeso ou obesidade, indicação endossada pelas principais sociedades de menopausa.^334,335^

#### 8.4.2. Dislipidemia

A TM está associada a risco aumentado de DCV, atribuído principalmente à DLP aterogênica, obesidade central e resistência à insulina, além de aumento do risco de HAS.^13^ A AHA afirma que adultos entre 40 anos e 75 anos devem ser submetidos a estimativa de risco de doença aterosclerótica CV em 10 anos.^334,335^ A SBC preconiza que, para mulheres com baixo RCV, o LDL-c deve ser inferior a 130 mg/dL e, caso a meta seja atingida, a terapia estrogênica deve ser preferencialmente utilizada por via transdérmica para evitar o fenômeno da primeira passagem hepática (pela via oral) e, nas pacientes não histerectomizadas, associa-se a progesterona micronizada com o objetivo de neutralizar os efeitos proliferativos do estrogênio sobre o endométrio.^13^

#### 8.4.3. Hipertensão

A HAS é um FR bem estabelecido para DCV e um agravante do RCV do DM nas mulheres.^13^ No estudo WHI, a utilização de estrogênios equinos conjugados associados ou não ao acetato de medroxiprogesterona demonstrou elevação nos níveis pressóricos de 1-1,5 mmHg.^335^ Na parte observacional do estudo WHI, a terapia estrogênica transdérmica apresentou baixo risco de desenvolvimento de HAS quando comparada ao uso oral.^335^ Pacientes com HAS não controlada (PA ≥ 180/110 mmHg) têm contraindicação para iniciar a THM em função do risco de AVC.

Dados observacionais e meta-análises mostram risco reduzido de DAC em mulheres que iniciam THM em idade inferior a 60 anos ou dentro de 10 anos após o início da menopausa. Segundo a NAMS, se a HAS estiver controlada e a estratificação de RCV for <5%, a THM oral ou transdérmica pode ser utilizada. Entretanto, em situações de difícil controle da HAS em mulheres com SVM moderados a graves, sugere-se a utilização da terapia estrogênica transdérmica associada à progesterona micronizada, em mulheres não histerectomizadas, por ter menor potencial trombogênico que outros progestagênios.^179,334^

#### 8.4.4. Diabetes

A prevalência de DM aumenta com a elevação da prevalência de obesidade.^13^ A THM não é contraindicada em mulheres saudáveis com DM2 preexistente e pode ser benéfica no controle glicêmico quando usada para o alívio dos sintomas da menopausa. A THM reduz significativamente o diagnóstico de DM2 de início recente,^325^ mas não é aprovada para este fim. Para mulheres com DM2 bem controlado (HbA1c < 8%) e SVM moderados a graves, a terapia estrogênica por via transdérmica, evitando o fenômeno da primeira passagem hepática, associada à progesterona micronizada seria a melhor opção terapêutica.^179,334,335^

#### 8.4.5. Síndrome Metabólica

Recente meta-análise demonstrou que a THM reduziu múltiplos componentes da SM, como obesidade abdominal, resistência à insulina, DLP e HAS.^336^ No estudo WHI, mulheres com SM randomizadas para utilização de estrogênio equino conjugado isolado ou associado a acetato de medroxiprogesterona tiveram risco 2 vezes maior de DCV que o grupo placebo.^335^ Apesar da ausência de estudos randomizados comparando as diferentes vias de administração da THM e evento CV em mulheres com SM, recomenda-se a utilização da THM transdérmica para o alívio dos sintomas em função da não ocorrência do fenômeno da primeira passagem hepática com essa via de administração.^334,335^

### 8.5. Prescrição para Pacientes com Doença Cardiovascular Manifesta

Dados obtidos de meta-análise não mostraram diferenças significativas entre usuárias de THM e grupo controle em relação aos desfechos primários de IM não fatal, morte CV e AVC em mulheres com DCV. A frequência de angina, insuficiência cardíaca e ataque isquêmico transitório também não diferiu entre os grupos THM e controle.^337^

#### 8.5.1. Infarto Agudo do Miocárdio

No geral, a THM está contraindicada em mulheres com DAC conhecida, incluindo IAM e doença arterial periférica.^335^ A THM em mulheres na pós-menopausa inicial (<10 anos de pós-menopausa) reduziu ou não teve efeito na progressão da aterosclerose subclínica e na calcificação da artéria coronária em ensaios randomizados e controlados.^179^ Estudo publicado em 2001 demonstrou que nenhuma diferença no risco de IAM recorrente ou morte por DCV em mulheres após o primeiro IAM foi observada entre usuárias e não usuárias de THM.^338^ O estudo observacional com 14 anos de seguimento, ESPRIT RCT (*Estrogen for the Prevention of Reinfarction Trial*), com mulheres pós-IAM e randomizadas para 2 anos de utilização de valerato de estradiol isolado ou placebo não demonstrou diferença entre os grupos em relação à morte por DCV, AVC ou DIC.^338^ Os atuais consensos não fazem estratificação do risco da THM com o subtipo de DCV apresentado pela mulher. O *American College of Cardiology* argumenta que, para mulheres com idade de 50-59 anos e história de IAM sem DAC obstrutiva, dissecção espontânea da artéria coronária, disfunção microvascular coronariana ou vasoespasmo coronariano, é necessária uma abordagem individualizada para THM. Pela suposta associação fisiopatológica entre os hormônios sexuais femininos e a dissecção espontânea da artéria coronária, recomenda-se que a THM via oral seja evitada nesse grupo e que, após controle adequado dos níveis pressóricos e das frações lipídicas, a THM sistêmica possa ser considerada.^335^ Acrescenta ainda que, para mulheres com estratificação de risco DAC > 10%, a THM deve ser evitada independentemente da via de administração dos hormônios.^335^

#### 8.5.2. Angina Instável

Dados provenientes de recente meta-análise, que analisou sintomas de angina e hospitalização por angina estável em 6 estudos, demonstraram que a hospitalização ocorreu predominantemente nos primeiros 2 anos após a randomização, com tendência não significativa de ser maior no grupo THM em estudo observacional. E, nos 5 ensaios clínicos randomizados, não houve diferença na angina entre os grupos THM e controle.^337^

A despeito disso, em caso de angina instável sugere-se que a THM por qualquer via de administração seja evitada, já que é considerada de alto risco em mulheres com DCV preexistente ou risco de DCV em 10 anos >10%.

#### 8.5.3. Acidente Vascular Cerebral e Acidente Isquêmico Transitório

O histórico prévio de AVC isquêmico é uma contraindicação à THM apesar de o risco absoluto, tanto da terapia estrogênica isolada quanto associada a progestagênio, ser considerado baixo (<10/10.000/ano).^334,335^

#### 8.5.4. Insuficiência Cardíaca

Existem poucos dados na literatura quanto ao uso da THM em mulheres com diagnóstico de insuficiência cardíaca. Se a paciente tem melhora clínica, fração de ejeção que retorna ao patamar anterior e bom controle de fatores modificáveis, a THM transdérmica em baixas doses pode ser indicada, caso não haja resposta com o tratamento não hormonal.^334^

#### 8.5.5. Tromboembolismo Venoso e Embolia Pulmonar

História prévia de TEV, incluindo TVP e EP, deve ser considerada contraindicação à THM sistêmica por via oral.^179,335^ Doses mais baixas de THM oral podem conferir menor risco de TEV do que doses mais altas, mas faltam dados comparativos de ensaios clínicos randomizados. A progesterona micronizada parece ser menos trombogênica que outros progestagênios utilizados em THM. A THM transdérmica não foi associada ao risco de TEV em estudos observacionais, e uma revisão sistemática confirma esses achados, sugerindo menor risco com a THM transdérmica do que com a oral. No entanto, faltam dados comparativos de ensaios clínicos randomizados.^179^ Se houver TEV diagnosticado em contexto de aumento de níveis estrogênicos como gravidez ou uso de anticoncepcionais orais, a THM deve ser evitada. A ocorrência do evento após imobilização prolongada ou trauma, como acidente automobilístico, queda ou pós-operatório imediato, indica que o estado pró-inflamatório que ocasionou a trombose pode não estar relacionado aos efeitos do estrogênio. Assim, nessas situações, pode-se utilizar a THM pela via transdérmica.^179,334^

#### 8.5.6. Doença Cardíaca Congênita e Pós-transplante Cardíaco

Em função da ausência de dados na literatura, recomenda-se cautela ao indicar a THM e, preferencialmente, os tratamentos não hormonais são considerados a melhor opção.

### 8.6. Hormônios Bioidênticos e Implantes: o que Precisa Ser Informado

Nos últimos anos, tem-se divulgado muito na mídia a terapia de reposição hormonal através do uso de hormônios bioidênticos e implantes hormonais, o que tem gerado muitas controvérsias e debates.

Hormônios bioidênticos são moléculas que apresentam a mesma estrutura química e molecular dos hormônios sintetizados pelo corpo humano. São cópias exatas de hormônios humanos endógenos, como estradiol, estriol e progesterona.^179^

O termo "bioidêntico" tem sido erroneamente divulgado como se referindo a novas opções terapêuticas de "hormônios naturais" feitas de modo personalizado em farmácias de manipulação para tratar os sintomas da menopausa. Entretanto, o primeiro ponto a ser esclarecido é que os hormônios bioidênticos mais usados na THM - estradiol, estriol e progesterona - são produzidos há anos pela indústria farmacêutica em diversas doses e formulações e são vendidos em farmácias comerciais.

Na verdade, existe a preocupação de que as terapias hormonais manipuladas possam apresentar inconsistência de dosagens, controle de qualidade e absorção, uma vez que formulações hormonais manipuladas podem apresentar diferenças farmacocinéticas e não ter o mesmo controle de qualidade nas diversas farmácias de manipulação do país.

A prescrição de THM através de implantes hormonais também se popularizou na mídia e redes sociais. Os implantes hormonais são cápsulas ou "pellets" que são inseridos no tecido subcutâneo através de um trocater, podendo ser absorvíveis ou não absorvíveis.

Os implantes manipulados podem diferir quanto à sua composição de hormônios (estradiol, testosterona, dihidrotestosterona, androstenediona, ocitocina, oxandrolona, nestorone, gestrinona) liberados na corrente sanguínea. Esses implantes também são produzidos por farmácias de manipulação e regulamentados por regras menos rígidas, não sendo obrigados a atender ao rigor e às exigências para medicamentos industrializados aprovados pelas agências reguladoras de medicamentos. Uma vez que não há exigência de bula para produtos manipulados, a dose e o tipo de hormônio contido em cada implante não estão preestabelecidos e não há alerta ou descrição de informações básicas, como indicações, estudos de segurança e possíveis efeitos adversos, como é exigido para outros medicamentos industrializados aprovados pela Agência Nacional de Vigilância Sanitária (ANVISA). Isso gera preocupação em relação às doses e ao conteúdo desses implantes, uma vez que podem expor as pacientes a danos ou riscos decorrentes da superdosagem ou a hormônios não indicados para THM.^339^

Uma busca na maior base de dados científicos mantidos pela *National Library of Medicine*, PubMed, mostra que existem poucos estudos clínicos sobre implantes hormonais para uso em THM. Os poucos estudos existentes são, na maioria, sobre implantes de testosterona e apresentam casuística pequena e metodologia com baixo grau de evidência (estudos retrospectivos ou observacionais), o que não permite conhecer os efeitos desses implantes sobre inúmeras questões relevantes em THM, como risco de câncer de mama e de endométrio, efeitos metabólicos e CV a longo prazo, para que possam ser usados com segurança em mulheres. Além disso, grande parte da farmacocinética dos implantes manipulados, como taxas de liberação dos hormônios, a grande variabilidade interindividual^340^ e a dificuldade na reversibilidade dos implantes caso ocorram efeitos adversos, são desconhecidos. Apesar de existirem poucos estudos com implantes com a participação de número expressivo de mulheres, as metodologias usadas apresentam limitações e vieses..^341,342^

Por outro lado, dados do PubMed mostram que todo o conhecimento científico acumulado ao longo de anos através de inúmeras publicações sobre os benefícios e riscos da THM relacionados a dose, vias de administração oral ou transdérmica, tipo de estrogênio e progestagênio vem de hormônios bioidênticos industrializados ou hormônios sintéticos aprovados por agências reguladoras. Esses estudos mostram diferenças entre si e seus resultados certamente não podem ser extrapolados para a via de implante até que estudos desenhados com esse propósito sejam publicados.

Novas alternativas ou vias de utilização da THM são muito bem-vindas para contemplar as necessidades de maior número de mulheres, mas só poderão ser recomendadas mediante comprovada eficácia e, principalmente, segurança.

Assim, frente à falta de estudos científicos que comprovem a segurança dos hormônios bioidênticos manipulados e dos implantes hormonais e as inúmeras dúvidas em relação a seus efeitos clínicos e riscos potenciais, essas formas de terapia hormonal não são recomendadas por sociedades médicas nacionais, como FEBRASGO,^343^ SOBRAC e Sociedade Brasileira de Endocrinologia, nem por sociedades médicas internacionais.

### 8.7. Terapias Adicionais

Existem algumas situações clínicas em que é necessário o controle dos SVM com terapias não hormonais. Isso se aplica às mulheres sintomáticas com contraindicações ou aquelas que, por preferências pessoais, não querem fazer uso de THM.

Essas terapias podem ser divididas em farmacológicas, farmacológicas alternativas (fitoterápicos) e não farmacológicas ou comportamentais.

#### 8.7.1. Terapias Farmacológicas

Entre as terapias não hormonais para alívio das ondas de calor, os inibidores seletivos da recaptação da serotonina e inibidores seletivos da recaptação da serotonina-norepinefrina atuam através da regulação dos níveis de serotonina e norepinefrina no centro termorregulador do hipotálamo, reduzindo as ondas de calor.

Paroxetina 7,5-25 mg, citalopram 10-20 mg, escitalopram 20 mg, venlafaxina 37,5-75 mg e desvenlafaxina 50-100 mg são os antidepressivos mais estudados, e ensaios clínicos randomizados controlados por placebo mostram que são efetivos em reduzir a frequência e a gravidade dos SVM leves a moderados.^344^ Resultados menos consistentes foram obtidos com sertralina e fluoxetina e, por isso, não são recomendados.^345^

A gabapentina é um anticonvulsivante análogo ao ácido gama-aminobutírico capaz de atravessar a barreira hematoencefálica e atuar diretamente no centro termorregulador do hipotálamo. Ensaios clínicos randomizados e controlados mostram eficácia em reduzir os SVM em doses que variam de 900 mg a 2.400 mg divididas em três tomadas. Os eventos adversos incluem sonolência, tontura e alterações do equilíbrio, podendo ser uma boa escolha para mulheres com alterações do sono associadas a SVM.^346^ Já a pregabalina, um derivado do ácido aminobutírico relacionado à gabapentina, tem sido avaliada na dose de 75-150 mg; entretanto, devido a poucos estudos e aos potenciais efeitos adversos, não tem sido recomendada para controle das ondas de calor.^347^ A oxibutinina, um anticolinérgico usado para tratamento da incontinência urinária de urgência na dose de 5-15 mg/dia, também foi avaliada em alguns poucos ensaios clínicos, mostrando melhora dos SVM; entretanto, deve ser usada com cautela em pessoas idosas.^347^

O novo medicamento fezolinetante, um antagonista da neuroquinina B que atua diretamente no centro termorregulador do hipotálamo, foi recentemente aprovado pela agência FDA para alívio dos SVM. Ensaios clínicos mostram que a dose de 45 mg por dia é eficaz em reduzir os SVM.^301^ Essa medicação em breve estará disponível no Brasil.

#### 8.7.2. Terapias Farmacológicas Alternativas

Terapias farmacológicas alternativas, como o emprego de fitoterápicos, também têm sido avaliadas para tratamento dos SVM. Isoflavonas são compostos não esteroides, encontrados em plantas e vegetais, com um anel fenólico e estrutura semelhante ao estradiol, apresentando alta afinidade e adesão aos receptores hormonais, agindo como agonista ou antagonista estrogênico. Estudos com isoflavonas de soja, como glicina max e *Trifolium pratense* (*red clover*) têm mostrado resultados conflitantes, alguns com benefícios e outros sem benefícios quanto à redução dos SVM em relação a placebo.^348^ A cimicifuga ou *Actae racemosa* L. (*black cohosh*) é um fitoterápico cujo mecanismo de ação ainda não está totalmente esclarecido e cujos resultados também são conflitantes.^349^ Outros compostos, como a erva-de-são-joão, ginkgo biloba e ginseng, não têm eficácia comprovada, não sendo recomendados.^347^

#### 8.7.3 Terapias Comportamentais

Terapias comportamentais, incluindo mudanças no estilo de vida, exercícios físicos, ioga e *mindfulness*, não têm se mostrado eficazes em reduzir os SVM. Terapias cognitivo-comportamentais, incluindo psicoeducação, entendimento de como as emoções afetam a percepção das sensações físicas e modificação dos gatilhos dos SVM parecem reduzir o desconforto associado aos mesmos.^347^

**Figura 8.2:**
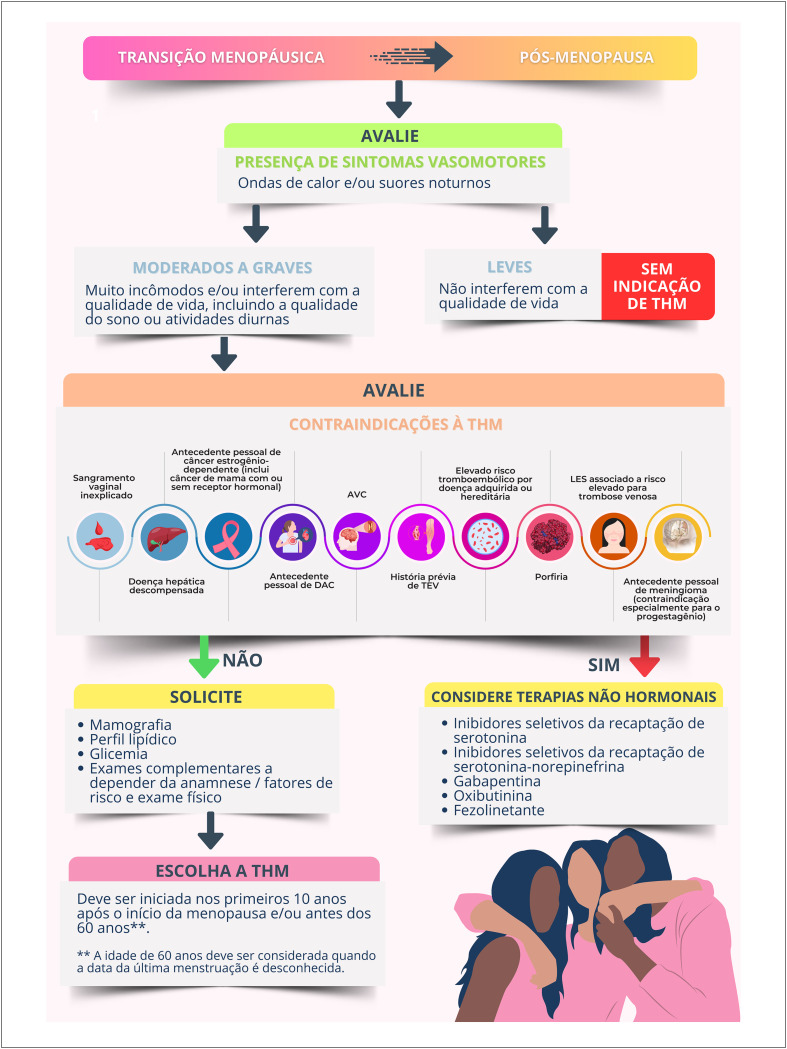
Fluxograma para a implementação das recomendações atuais de terapia hormonal da menopausa. THM: terapia hormonal da menopausa; DAC: doença arterial coronariana; AVC: acidente vascular cerebral; TEV: tromboembolismo venoso; LES: lúpus eritematoso sistêmico.

A Figura 8.2 descreve um fluxograma para a implementação das recomendações atuais para a THM. O Quadro 8.1 descreve os tipos, doses e vias de administração dos estrogênios e progestagênios utilizados na THM.

**Quadro 8.1:** Tipos, doses e vias de administração dos estrogênios e progestagênios utilizados na terapia hormonal da menopausa (THM).

**TIPOS, DOSES E VIAS DE ADMINISTRAÇÃO DOS ESTROGÊNIOS UTILIZADOS EM THM**	
**VIA ORAL**	
17 β estradiol	0,5 - 1,0 - 2,0 mg/dia
Valerato de estradiol	1,0 - 2,0 mg/dia
**VIA TRANSDÉRMICA**	
17 β estradiol gel	0,5 - 0,75 - 1,0 - 1,5 a 3mg/dia
17 β estradiol adesivo	25 - 50 mcg/dia
**VIA VAGINAL**	
Estriol creme	1,0 mg/g
Estradiol	10 mcg por comprimido
Promestrieno	10 mg/g
**TIPOS, DOSES E VISAS DE ADMINISTRAÇÃO DOS PROSTAGÊNIOS UTILIZADOS EM THM**	
**VIA ORAL**	
Acetato de medroxiprogesterona	2,5 a 10 mg/dia
Acetato de ciproterona	1 - 2 mg/dia
Acetato de noretisterona	0,1 a 1 mg/dia
Acetato de nomegestrol	2,5 - 5 mg/dia
Didrogesterona	5 - 10 mg/dia
Drospirenona	2 mg/dia
Progesterona micronizada	100 - 200 mg/dia
**VIA TRANSDÉRMICA**	
Acetato de noretisterona	140 - 170 mcg/dia
**VIA VAGINAL**	
Progesterona micronizada cápsulas moles	100 - 200 mg/dia
**VIA INTRAUTERINA**	
DIU-levonorgestrel	20 mcg/dia

## 9. Menopausa e a Mulher no Mercado de Trabalho – Dificuldades e Oportunidades de Melhorias

### 9.1. Introdução

Virtualmente, todas as mulheres passarão pela menopausa e, na TM, a grande maioria apresentará inúmeros sinais e sintomas, muitos dos quais comprometem a qualidade de vida.

Em 2020, estatísticas globais detectavam 657 milhões de mulheres com idade entre 45 anos e 59 anos, sendo que 47% se encontravam no mercado de trabalho. Como a média da idade da menopausa se situa em torno de 51 anos, pode-se prever um grande contingente de mulheres trabalhadoras nessa etapa da vida.^350^

Na economia atual, os rendimentos do trabalho das mulheres tornaram-se uma necessidade para elas e seus familiares, de forma que permanecer trabalhando durante a menopausa faz parte de sua realidade. Com o aumento da expectativa de vida e da idade ativa das mulheres, muitas passam um terço de suas existências na menopausa e na pós-menopausa, com uma proporção significativa de anos de vida profissional nessa etapa.

O ato de trabalhar, além da óbvia necessidade financeira, está relacionado com maior autoestima, melhor saúde e menos estresse psicológico.

O impacto dos sintomas da menopausa e das doenças a ela relacionadas no ambiente de trabalho merece maior atenção profissional do que ocorre na atualidade. É necessário observar o efeito negativo dos sintomas da menopausa na capacidade para o trabalho das mulheres nessa etapa.^351,352^

Existe uma diversidade de experiências de mulheres menopausadas no local de trabalho, moldada não apenas pelos sintomas e contexto da menopausa, mas também pelas características físicas e psicossociais do ambiente de trabalho. Esses fatores podem afetar não apenas a qualidade de vida, mas também a motivação e o envolvimento com as atividades profissionais, o desempenho cotidiano e as relações com os empregadores.

Em sociedades ou ambientes nos quais a menopausa é considerada um assunto tabu, a falta de discussão e o estigma sobre ela aumentam o fardo dos sintomas para as mulheres.^353^

Estudos mostram que os sintomas que mais perturbam as mulheres no trabalho são os urinários, fadiga, dificuldade para dormir, falta de concentração e de memória, sensação de desânimo/depressão e redução da autoconfiança.

As mulheres com sintomas graves da menopausa podem abandonar o emprego ou reduzir seu horário de trabalho, o que afeta negativamente o rendimento e a segurança futura na vida. Para os empregadores, isso significa a perda de pessoal experiente com competências e talentos valiosos.

Ao longo da última década, tem havido um aumento na conscientização entre os empregadores sobre a menopausa como um potencial problema de saúde ocupacional e sobre a necessidade de apoio adequado no local de trabalho. A sensibilização dos gestores em relação a horários flexíveis foi considerada um apoio benéfico às trabalhadoras.

Faz-se necessário reconhecer que os sintomas da menopausa podem afetar negativamente a capacidade laboral e que as condições de trabalho podem afetar os sintomas da menopausa. Por isso, é fundamental a criação de um ambiente de trabalho aberto às necessidades específicas de saúde das mulheres, com cultura inclusiva e de apoio, sem discriminação àquelas que passam por e/ou padecem nesse período difícil e fisiológico da vida.^350,353^

### 9.2. Impacto dos Sintomas da Menopausa na Vida Profissional e como as Mulheres Lidam com as Mudanças Biológicas nessa Etapa

#### 9.2.1. Sinais e Sintomas e Vida Profissional na Menopausa

Um dos grupos de trabalho que mais cresce na atualidade é o de mulheres com idade acima de 50 anos, com as taxas de emprego entre as pessoas com idades de 50-64 anos variando entre 55% e 67% na Europa, Austrália e EUA.^350^

**Figura 9.1:**
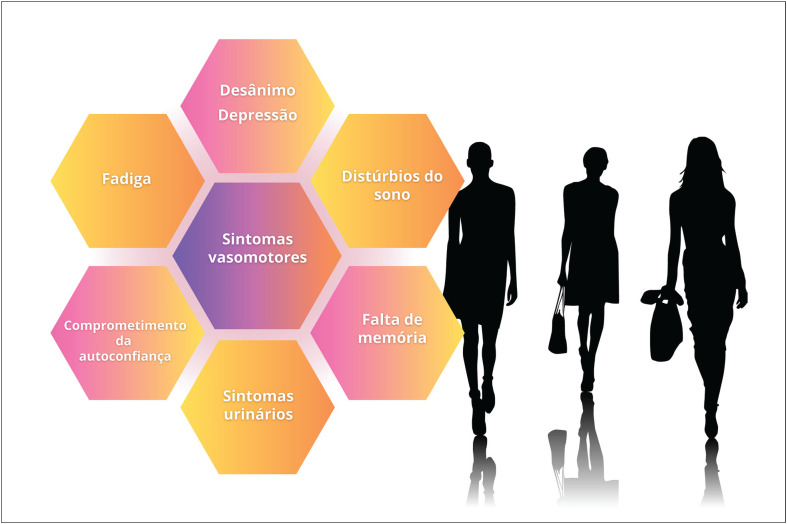
Sintomas da menopausa com impacto negativo no desempenho profissional.

Os sintomas da menopausa afetam negativamente as mulheres que trabalham, comprometendo seu desempenho e reduzindo a satisfação no trabalho, além de prejudicar o bem-estar profissional individual, o que pode resultar em custos diretos e indiretos para o empregador e para o Estado (Figura 9.1).

Como apontado anteriormente, a perda do efeito protetor do estrogênio e as consequentes mudanças relacionadas levam a um aumento do RCV nos anos pós-menopausa, tendo sido demonstrado que mais da metade das mulheres desenvolve HAS. Aumento dos níveis de colesterol, do peso corporal e da incidência de DM2 também é frequente e contribui para elevar o RCV.^351^

A menopausa é considerada de forma diversa nas diferentes culturas, sendo vista mais positivamente nas que valorizam a experiência e a sabedoria do envelhecimento. Isso tem efeitos práticos, pois mulheres que encaram a menopausa como algo negativo irão apresentar mais sintomas.

Além disso, a menopausa, por vezes, ocorre em uma fase da vida difícil, em que os filhos "abandonaram o ninho", em que muitas perdem seus pais ou têm que se dedicar a cuidar de pais ou outros parentes idosos, além da ocorrência de problemas de saúde associados à própria idade. Essas situações aumentam a possibilidade do desenvolvimento de depressão, ansiedade, estresse crônico e síndrome de "*burnout*".

É importante observar que algumas queixas de saúde associadas ao "*burnout*", como fadiga, distúrbios do sono, problemas cognitivos, como dificuldade de concentração e de memória, irritabilidade e distúrbios emocionais, podem se assemelhar aos sintomas da menopausa e diferenciá-los nem sempre é fácil para as mulheres ou mesmo para o profissional de saúde.^351^

Os sintomas mais frequentes em resposta às mudanças hormonais da menopausa são ondas de calor, sudorese noturna, distúrbio do sono, fadiga, dores musculares, ressecamento vaginal, distúrbios urinários, mudança do humor, cefaleia, problemas de concentração e memória. Insônia e depressão na peri- e pós-menopausa associam-se a perda da produtividade e a custos elevados para o empregador.

As típicas ondas de calor geralmente aparecem de forma súbita e se espalham do tórax para pescoço, cabeça e braços, sendo secundárias ao aumento do fluxo sanguíneo para a pele, acompanhado de aumento da FC, e seguidas por suor na parte superior do corpo. O aumento da FC secundária ao maior fluxo periférico visa manter o débito cardíaco adequado para manter a PA. O efeito dos calores sobre a FC é ainda maior durante a realização de trabalhos pesados ou em temperaturas elevadas.^354^ Podem ocorrer sintomas objetivos ocasionais, como fluxo menstrual aumentado e incontinência urinária, além de queixas menos específicas, como sintomas cognitivos e psicológicos.

Em um estudo no qual 407 mulheres que trabalham nas áreas da saúde e de serviços sociais responderam a um amplo questionário acerca da menopausa, observou-se que o início dos sintomas ocorreu entre 46 anos e 52 anos em 43% das mulheres e entre 41 anos e 45 anos em 35% delas.^353^ Há de se considerar também os efeitos físicos e psicológicos da menopausa cirúrgica, observados geralmente em mulheres mais jovens.^353^

#### 9.2.2. Menopausa e o Ambiente de Trabalho

Em geral, o trabalho melhora a saúde mental das mulheres, já que afeta positivamente sua autoestima e sua saúde e diminui o estresse psicológico.^351^ Mulheres que relatam boa saúde apresentam menor influência dos sintomas da menopausa no trabalho.

Os sintomas podem começar 4 anos antes e durar por mais 4-8 anos após o início da menopausa. Assim, o contingente de trabalhadoras sintomáticas, especialmente em áreas de trabalho onde há prevalência de mulheres, é muito expressivo, o que tem levado, nos últimos anos, a uma maior conscientização dos empregadores sobre a menopausa como uma preocupação de saúde ocupacional e consequente necessidade de apoio a essas mulheres.^353^

A menopausa raras vezes é discutida no local de trabalho, possivelmente por tabus, o que contribui para a falta de conhecimento sobre essa fase de vida entre as próprias mulheres, os profissionais de saúde e os empregadores. Em ambientes de trabalho onde as mulheres são minoria (polícia, militar, indústria etc.), essas dificuldades de abordagem da menopausa podem ser ainda mais limitantes. Assim, apesar da realidade apresentada, os estudos que avaliam o impacto da menopausa no trabalho e na carreira das mulheres são ainda muito escassos.

Um estudo realizado na Holanda demonstrou que mulheres com sintomas graves da menopausa apresentaram redução da capacidade de trabalho 8,4 vezes maior que aquelas pareadas para a idade, porém sem esses sintomas. Além do mais, houve maior risco de absenteísmo, com maiores prejuízos financeiros.^355^

O efeito agudo dos calores da menopausa pode gerar um impacto negativo no desempenho profissional, afetando a capacidade de se concentrar e de completar tarefas cognitivas complexas durante a onda de calor e de suores desconfortáveis.

Mulheres que apresentam suores noturnos têm o ciclo natural de sono afetado, às vezes com graves consequências crônicas. A privação do sono adequado leva a maiores distúrbios do sono e consequente insônia e fadiga durante o dia. Assim, dentro do ambiente de trabalho, o impacto latente dos suores noturnos pode se manifestar como uma capacidade reduzida de trabalhar e produzir, com maiores chances de erros e consequentes acidentes de trabalho.^354^

O estudo de O’Neill, que avaliou o impacto dos sintomas da menopausa na vida laborativa de 407 mulheres, mostrou que 65% das participantes relataram que os sintomas afetam sua capacidade de trabalhar, 35% afirmaram que eles influenciaram na decisão da progressão de suas carreiras, 18% descreveram faltas ocasionais do trabalho devido a seus sintomas, 8% diminuíram sua carga horária, 7% mudaram de função, 6% pararam de trabalhar no turno da noite e 2% pararam completamente de trabalhar devido aos sintomas.^353^

Essa realidade do importante impacto negativo dos sintomas da menopausa na vida profissional de um grande contingente de mulheres aponta para a necessidade premente de mudanças nos seus locais de trabalho. Essas mudanças visam mitigar os problemas de diminuição da capacidade laborativa e do absenteísmo relacionados a esses sintomas.

Quando perguntadas sobre o que precisa mudar no local de trabalho para amenizar o impacto dos sintomas da menopausa sobre sua capacidade laborativa, um terço das mulheres no estudo de O’Neill mencionou que a conscientização de seus empregadores seria o fator mais importante, seguida de horários de trabalho flexíveis. Foi também mencionado que controle de temperatura e ventilação, acesso fácil a banheiros e fontes de água potável, além do uso de uniformes leves, também seriam de grande ajuda. Em termos de oferta de tratamentos, os recursos mais mencionados por essas mulheres em seu ambiente de trabalho foram a consulta com clínicos gerais, uso de terapia hormonal e antidepressivos. Um achado interessante desse estudo foi que os sintomas neurocognitivos e psicológicos tiveram maior impacto, enquanto os SVM tiveram menos influência no trabalho dessas mulheres.^353^

Assim, é importante que profissionais da saúde e empregadores sejam capazes de reconhecer que os sintomas da menopausa podem afetar drasticamente o bem-estar das mulheres que trabalham e, consequentemente, a qualidade de suas vidas e capacidade laboral, o que irá levar à redução das horas trabalhadas, com subemprego ou mesmo desemprego e suas consequências financeiras nefastas na vida das mulheres.^350^

### 9.3. Empregadores e Menopausa – Oportunidades de Melhoria

Na Grã-Bretanha, em 1971, havia 3 milhões de mulheres empregadas com idades de 45-59 anos, um número que subiu para 3,9 milhões em 2001, representando agora 45% da força de trabalho com mais de 50 anos.^356^

A menopausa não é uma experiência uniforme entre as mulheres trabalhadoras e há grande variabilidade na prevalência de sintomas que geram dificuldades no trabalho (de 25% a 65%), de acordo com diferentes estudos.

Mulheres sintomáticas apresentam maiores níveis de absenteísmo (licença médica) e maior frequência de consultas ambulatoriais. Os custos por incapacidade para o trabalho aumentam significativamente naquelas com sintomas graves em comparação àquelas com sintomas leves, observando-se que SVM, insônia ou distúrbios do sono e sintomas psicossociais têm o maior impacto.^357^

Os resultados do recém-publicado *Health and Employment after Fifty Study (HEAF)* mostraram que, enquanto os sintomas mais frequentemente mencionados por mulheres que trabalham na peri- e pós-menopausa foram os vasomotores (91,7%), dificuldade para dormir (68,2%), psicológicos (63,6%) e urinários (49,1%), os sintomas que mais comprometeram a capacidade laboral foram os psicológicos (depressão, irritabilidade, ansiedade, choro), cefaleia grave e dores articulares.^358^

Nesse estudo, os FR identificados para a ocorrência de maiores dificuldades de enfrentamento dos sintomas da menopausa no trabalho foram a privação financeira, pior autopercepção de saúde, depressão e fatores psicossociais ocupacionais adversos.^358^

Os autores concluíram que a desigualdade também afeta as trabalhadoras na menopausa e que é necessária uma maior conscientização entre os empregadores de todos os setores, mas atenção especial deve ser dada às mulheres vulneráveis (com dificuldades econômicas, com empregos nos quais se sentem inseguras, desvalorizadas ou insatisfeitas), pois encontram-se sob maior risco.^358^

Problemas com supervisores ou colegas, como a percepção de falta de apoio ou a probabilidade percebida de que a menção à menopausa provocará respostas constrangedoras, irrelevantes ou discriminatórias, também foram relatados como causadores de dificuldades e levam a uma falta de vontade por parte da mulher em revelar a condição menopausa.^359^

De acordo com o estudo de Reynolds, as mulheres sofrem por SVM no trabalho, com acentuação do desconforto durante reuniões formais, espaços fechados e quentes e na presença de homens. A abordagem desse problema tem sido vista em grande parte como uma responsabilidade individual e não da organização. Reynolds sugere que os conselheiros e agentes de saúde ocupacional nas organizações devam aumentar a conscientização sobre as necessidades das mulheres no nível da gestão, com sensibilização para o impacto potencial das ondas de calor nas experiências de trabalho e preparando-se para oferecer apoio e aconselhamento. O simples fornecimento de ventiladores e sistemas de aquecimento controláveis é uma ajuda prática para os problemas mais comuns dessa etapa.^360^

Se o problema é visto como pessoal, a revelação é permitida quando as próprias mulheres têm atitudes positivas em relação ao envelhecimento ou têm colegas que consideram empáticos. Por outro lado, ao enquadrar o problema como organizacional, discutir e negociar as necessidades subjacentes pode levar à geração de alternativas para resolver os problemas, como horários flexíveis, mudança para meio período por um determinado tempo, trabalho remoto ocasional, ajuste de termostatos, discussão de férias anuais, mudança de localização dos escritórios (áreas mais ventiladas e iluminadas, mais próximas a banheiros) e conversas com supervisores em busca de soluções criativas.^359^

Os membros das organizações muitas vezes se conscientizam da idade de uma mulher através da constelação de sintomas da menopausa. Os empregadores têm demorado a reconhecer que as mulheres em idade de menopausa podem precisar de considerações especiais. As organizações devem buscar reduzir o estigma relacionado à menopausa e apresentá-lo como um ativo e não um passivo.

A revisão de Jack *et al.*^357^ sugere quatro recomendações:

1. Os empregadores devem rever o âmbito e o quadro das políticas, práticas e atividades relevantes em matéria de saúde e segurança no trabalho, bem como dos recursos humanos, tendo em conta os requisitos legais nacionais para proporcionar um ambiente de trabalho seguro, saudável e livre de discriminação para as trabalhadoras na perimenopausa, adotando uma abordagem holística que dependerá do setor empresarial;
2. Como parte de suas responsabilidades em segurança e saúde no trabalho, os empregadores devem realizar avaliações de risco com mulheres sintomáticas para identificar as adaptações razoáveis necessárias que podem ser feitas em seu ambiente de trabalho físico (e psicossocial). Isso vai depender do tipo/função do trabalho e da indústria;
3. Os empregadores devem desenvolver programas de promoção da saúde que incluam informações sobre menopausa, envelhecimento e saúde, permitindo às mulheres autogerenciarem os sintomas (por exemplo, através de mudanças alimentares, gestão do estresse, desenvolvimento de atitudes positivas em relação ao envelhecimento e à menopausa), bem como oferecer apoio social formal e informal (por exemplo, através de uma rede de mulheres ou através de reuniões à hora do almoço);
4. Uma variedade de políticas e atividades de treinamento e desenvolvimento organizacional e de recursos humanos deve ser implementada para gerar ambientes de trabalho mais hospitaleiros e relações supervisoras/subordinadas positivas e de apoio para mulheres na menopausa.

### 9.4. Como Melhorar as Condições de Trabalho

Na última década, houve um aumento na conscientização entre os empregadores sobre a menopausa como um potencial problema de saúde ocupacional, assim como sobre a necessidade da oferta de apoio adequado no local de trabalho. No entanto, ainda são escassas as ações nesse sentido, com quase nenhum diálogo entre empregador/gerente e funcionárias, na maioria das organizações.

É importante que os empregadores promovam uma cultura institucional onde seja aceitável discutir os sintomas da menopausa e que os gestores e supervisores recebam informações sobre a menopausa e sejam educados sobre como ter conversas de apoio com as funcionárias.

Estudo realizado na Clínica Mayo mostrou que os dias perdidos de trabalho atribuídos a sintomas da menopausa representam um custo anual aproximado de 1,8 bilhão de dólares.^361^

Controle de temperatura (72%), horários de trabalho flexíveis, incluindo compartilhamento de trabalho, folga para consultas médicas, intervalos regulares (58%), seminários sobre envelhecimento saudável (50%), espaços de trabalho flexíveis, como trabalho em casa, mudar para um escritório diferente (50%), programas de exercícios físicos (46%) e ventiladores de mesa (45%) seriam as medidas mais popularmente indicadas.^353^

A menopausa pode ser considerada um assunto tabu, que não é discutido no ambiente de trabalho, embora possa haver muitas colaboradoras afetadas nas instituições. Ser capaz de ter conversas apropriadas e ajustar o ambiente de trabalho às necessidades das mulheres perimenopáusicas afetará positivamente sua qualidade de vida, seu envolvimento, seu desempenho e sua motivação profissional. Algumas ações práticas no local de trabalho a serem utilizadas por gerentes e supervisores estão dispostas no Quadro 9.1.^350^

**Quadro 9.1:**
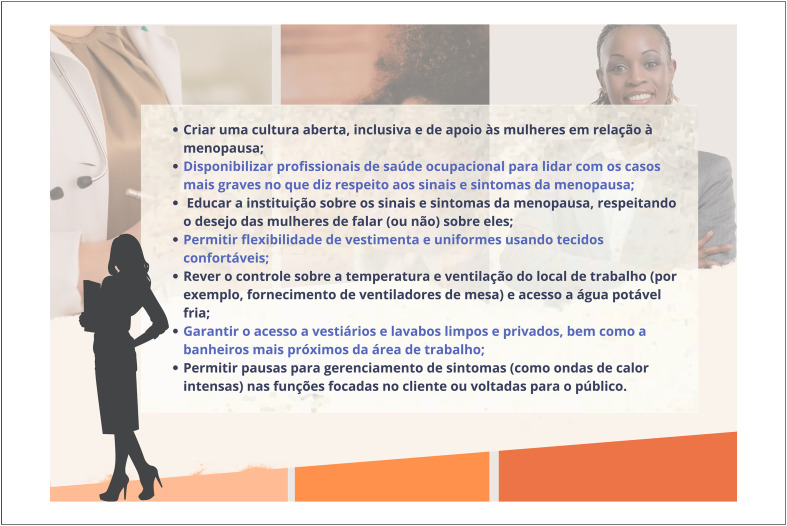
Ações práticas sobre a menopausa no ambiente de trabalho sugeridas aos empregadores.

### 9.5. Conclusões

Os estudos já realizados avaliam a frequência dos sintomas da menopausa, ao invés do impacto de sintomas específicos no trabalho, ou centram-se em resultados agrupados como a qualidade de vida ou estratégias de sobrevivência, esquecendo-se dos efeitos na carreira e no bem-estar profissional.

As mulheres constituem uma grande parte da força de trabalho global. É preciso avançar nesse cenário, tornando o ambiente de trabalho mais favorável às mulheres na menopausa, para melhorar seu bem-estar, bem como sua capacidade de permanecer no trabalho, garantindo assim que mais pessoas atinjam a aposentadoria com possibilidade de manter as contribuições para pensões e poupanças, suficientes para um rendimento e segurança adequados na vida.

## 10. Menopausa e Climatério na América Latina – Situação Atual, Desafios e Oportunidades de Intervenção

### 10.1. Introdução

A América Latina (AL) estende-se por mais de 20 milhões de km^2^ e engloba 20 países desde o México até o sul da América do Sul: Argentina, Bolívia, Brasil, Chile, Colômbia, Costa Rica, Cuba, Equador, El Salvador, Guatemala, Haiti, Honduras, México, Nicarágua, Panamá, Paraguai, Peru, República Dominicana, Uruguai e Venezuela.^362-364^

É uma região que possui muitas e diversas culturas em função da multiplicidade de idiomas (espanhol que é predominante, português, francês, inglês, holandês e inúmeras línguas nativas), etnias (branca, negra, indígena), relevo, clima, religiões e costumes. Cada país apresenta grande variabilidade dessas características, o que os torna únicos, a despeito de compartilharem uma mesma região geográfica e possuírem elementos históricos em comum.^362-364^

A região passou por um processo de industrialização tardia e urbanização rápida e acelerada, e a economia de seus países é baseada na exploração e exportação de recursos naturais, com papel importante também do setor terciário.^362-364^

A AL possui uma população estimada em 659.744.000 habitantes (ONU, 2022), dos quais aproximadamente 80% vivem em cidades, sendo as maiores delas São Paulo (Brasil), Cidade do México (México) e Buenos Aires (Argentina).^362-364^ As mulheres representam 51% da população total da região.^364^

A análise do perfil de expectativa de vida e das causas de morte de 363 cidades, de nove países da AL, demonstrou uma grande variabilidade desses indicadores entre os países e dentro de cada um.^365^

Observou-se que a expectativa de vida ao nascer na AL varia de 74 anos a 83 anos e de 63 anos a 77 anos, em mulheres e homens, respectivamente. Existem países com níveis mais elevados de expectativa de vida, como Panamá, Costa Rica e Chile, mas há grande variabilidade entre as cidades dentro de cada país, podendo atingir 7-10 anos, como é o caso do México, Brasil, Colômbia e Peru.^365^

As doenças crônicas (CV e outras) são a principal causa de morte, representando, em média, 57,7% da mortalidade total. Na AL, o câncer determina, em média, 16,2% da mortalidade geral; as causas transmissíveis, maternas, neonatais e nutricionais respondem, em média, por 14,4%, e as causas externas, em média, por 11,6%.^365^

Os resultados desse estudo também demonstraram que um maior nível de escolaridade, acesso a água e saneamento e viver em cidades menos populosas são preditores de maior expectativa de vida, de uma proporção relativamente menor de mortes por doenças transmissíveis, maternas, neonatais e nutricionais e uma maior proporção de mortes por DCV, câncer e outras doenças crônicas, em homens e mulheres.^365^

Os estudos demonstram que algumas mulheres percebem a menopausa como mais uma etapa natural da vida, sem implicações negativas associadas; entretanto, em muitas outras, as alterações hormonais do período podem gerar sintomas que afetam o bem-estar físico, emocional, mental e social.^15,366-368^

As modificações hormonais responsáveis pela menopausa determinam, assim, inúmeras alterações na saúde feminina, como aumento da perda óssea e perda da cognição, além de elevação do RCV no período pós-menopausa, graças à elevação da prevalência de DIC, AVC, doença vascular periférica, fenômenos tromboembólicos, FA e insuficiência cardíaca, bem como da mortalidade CV.^15,366-368^ Tais riscos não são reduzidos com a THM com estrogênios/progestagênios sintéticos.^179,255^

É importante também mencionar que há uma tendência secular de aumento na média de idade da menopausa em mulheres que vivem em países de alta renda. No entanto, observa-se, em países de baixa/média renda, um claro aumento na prevalência de IOP (antes dos 40 anos) e menopausa precoce (entre 40 anos e 44 anos),^369^ ambas consideradas na atualidade como FR para DCV e mortalidade CV.^15,368^

### 10.2. Mulher e Menopausa na América Latina

#### 10.2.1. Menopausa Natural, Insuficiência Ovariana Prematura e Menopausa Precoce

Castelo-Branco *et al*.,^370^ em 2006, analisando 17.150 mulheres saudáveis com idades entre 40 anos e 59 anos, em 47 cidades de 15 países latino-americanos, identificaram que a mediana de idade da menopausa para toda a amostra foi de 48,6 anos (43,8 anos a 53 anos).^12^ Observaram que mulheres com 49 anos, de menor escolaridade, que vivem em países com menor renda e em cidades a 2000 metros ou mais acima do nível do mar eram mais propensas a início mais precoce da menopausa.^370^

Uma meta-análise publicada por Schoenaker *et al.,*^371^ em 2014, que incluiu a investigação de Castelo-Branco *et al.*,^370^ demonstrou que a média de idade da menopausa natural na AL era 47,24 anos (45,9 anos a 48,6 anos).^371^ No estudo, realizado a partir de publicações que envolviam 24 países de seis continentes, os autores concluíram que a média de idade da menopausa natural era mais baixa nos países africanos (48,38 anos), latino-americanos (47,24 anos), asiáticos (48,75 anos) e do Oriente Médio (47,37 anos) e mais elevada na Europa (50,54 anos), na Austrália (51,25 anos) e nos Estados Unidos da América (49,11 anos), observando-se uma média de 48,8 anos (46 anos a 52 anos) para o conjunto dos seis continentes.^371^ Essa meta-análise também demonstrou que tabagismo e níveis mais baixos de escolaridade e ocupação encontram-se associados a idade mais precoce da menopausa natural.^371^

Leone, Brown e Gemmill^369^ conduziram estudo utilizando 302 pesquisas domiciliares padronizadas, realizadas de 1986 a 2019, com mulheres de 15-49 anos, em 76 países de baixa e média renda, em cinco regiões geográficas (Ásia Central; AL e Caribe; Norte da África/Oeste da Ásia/Europa; Sul e Sudeste Asiático; e África Subsaariana). Aqueles autores observaram prevalência crescente de IOP e menopausa precoce em países de baixa e média renda, em particular na África Subsaariana e Sul/Sudeste Asiático e que essas regiões também apresentavam uma redução da média de idade da menopausa, havendo grande variação entre os continentes. A Tabela 10.1 mostra os resultados dessas variáveis para as regiões estudadas.

**Tabela 10.1:** Prevalência de insuficiência ovariana prematura e menopausa precoce e média da idade da menopausa em diferentes regiões geográficas, em mulheres de 15-49 anos (modificada a partir de Leone, Brown e Gemmill^369^)

REGIÕES	INSUFICIÊNCIA OVARIANA PREMATURA (%)	MENOPAUSA PRECOCE (%)	MÉDIA DA IDADE DA MENOPAUSA (ANOS)
Ásia Central	1,2	1,6	45,3
América Latina e Caribe	1,5	1,9	44,4
Norte da África/Oeste da Ásia/Europa	0,1	1,4	44,7
Sul e Sudeste Asiático	2,7	4,5	43,7
África Subsaariana	0,9	2,4	44,1

Em comparação com estudos prévios, apenas a região conjunta do Norte da África/Oeste da Ásia/Europa apresentou redução na prevalência da IOP/menopausa precoce e elevação da média de idade da menopausa.^369^

O conjunto dessas informações chama a atenção para o fato de que a AL se apresenta entre as regiões geográficas com média de idade mais baixa para início da menopausa e com maior prevalência de IOP e menopausa precoce, quando comparada a regiões do mundo com maior desenvolvimento e maior renda.

Considerando que a menopausa (natural, IOP ou menopausa precoce) se constitui na atualidade em um FRCV, esse perfil mencionado pode contribuir, juntamente com os demais FR, para elevar o RCV das mulheres latino-americanas.

#### 10.2.2. Sintomas e Qualidade de Vida na Transição Menopáusica em Mulheres da América Latina

A TM determina o aparecimento de inúmeros sintomas que comprometem a qualidade de vida da mulher, dentre os quais podem ser citados os vasomotores (ondas de calor e suores, geralmente noturnos), insônia, alterações do humor (com maior propensão à depressão), irritabilidade, confusão mental, alterações genitais (secura vaginal, dor durante a atividade sexual, diminuição da libido), sintomas do trato urinário, dores musculares/articulares e palpitações.^372,373^ Os SVM podem durar 7-9 anos ou mais.^372^

##### A – Sintomas vasomotores

Em uma revisão sistemática de estudos realizados na Europa, América do Norte, AL e Ásia, publicados entre 1966 e 2009, Palacios *et al*. relataram que, além das diferenças geográficas observadas na média da idade de início da menopausa, existem diferenças quanto à frequência da sintomatologia mencionada pelas mulheres, mas os SVM estão entre os de maior prevalência em todas as regiões estudadas (Europa 74%; América do Norte 36-50%; AL 45-69%; Ásia 22-63%).^373^

Utilizando a Escala de Avaliação da Menopausa e um questionário para informações sociodemográficas, Blümel *et al.* avaliaram 8.373 mulheres, com idades entre 40 anos e 59 anos, de 22 centros de saúde, em 18 cidades de 12 países da AL e identificaram SVM em 55% da amostra, sendo que, em 9,6%, os sintomas se apresentaram com maior gravidade.^374^ A regressão logística demonstrou que a presença de sintomas psicológicos/urogenitais graves, menor escolaridade, peri- e pós-menopausa natural, nuliparidade, menopausa cirúrgica e morar em grandes altitudes foram FR significantes para SVM mais graves.^374^

Sánchez-Zarza *et al*. publicaram estudo transversal recente no qual 216 mulheres residentes em áreas urbanas de Assunção-Paraguai (40-60 anos) foram avaliadas com a Escala Cervantes de 10 itens e um questionário geral (dados pessoais e do parceiro). De acordo com a Escala Cervantes de 10 itens, os três sintomas mais prevalentes foram: dores musculares e/ou articulares (70,8%), ansiedade e nervosismo (70,8%) e SVM (54,2%).^375^

Em estudo de base populacional, com 1.500 mulheres brasileiras, com idades de 45-65 anos, Pompei *et al.* identificaram que a mediana da idade de início da menopausa foi 48 anos (de 45 anos a 51 anos), sem diferença entre as classes econômicas. Sintomas relacionados com a menopausa estavam presentes em 87,9% daquelas que se encontravam em TM, sendo os SVM os que apareceram mais precocemente.^376^

Com o objetivo de avaliar a prevalência e o impacto de SVM moderados a graves na pós-menopausa, foi realizado estudo transversal em 12.268 mulheres de 40-65 anos no Brasil, Canadá, México e quatro países nórdicos (Dinamarca, Finlândia, Noruega e Suécia). Observou-se que a prevalência de SVM moderados a graves foi de 15,6%, sendo mais elevada no Brasil (36,2%) e mais baixa na Europa nórdica (11,6%). Os SVM afetaram a qualidade de vida em todos os domínios avaliados.^377^

##### B – Distúrbios do sono

Avaliando 6.079 mulheres com idades entre 40 anos e 59 anos, de 11 países latino-americanos, utilizando inúmeros instrumentos de investigação, Blümel *et al.* observaram que 56,6% da amostra sofria de insônia, má qualidade do sono ou ambos, além de que a prevalência da insônia aumentou com a idade e da pré- para a pós-menopausa tardia.^378^ A regressão logística identificou que idade, doenças crônicas, consumo abusivo de álcool, ansiedade, depressão, SVM, uso de medicamentos (hipnóticos e THM) contribuíram positivamente para a ocorrência de distúrbios do sono, enquanto maior nível de escolaridade associou-se a menos insônia e melhor qualidade de sono.^378^

##### C – Dores musculares/articulares

Blümel *et al*.,^374^ utilizando a mesma amostra de mulheres latino-americanas anteriormente mencionada, avaliaram a relação de dores musculares/articulares com outras queixas da menopausa.^379^ Observou-se que 63% da amostra apresentava tais sintomas, sendo classificados como graves/muito graves em 15,6%. A regressão logística identificou que idade, tabagismo, menor escolaridade, SVM, IOP, pós-menopausa, acompanhamento psiquiátrico e uso de psicotrópicos foram significantemente relacionados a maior gravidade de sintomas, enquanto autopercepção de saúde, acesso privado à assistência à saúde e uso de THM foram significantemente relacionados a sintomas menos graves.^379^

##### D – Disfunção sexual

Através do questionário Índice de Função Sexual Feminina para investigação da disfunção sexual em 7.243 mulheres saudáveis com idade entre 40 anos e 59 anos, usuárias de 19 sistemas de saúde de 11 países latino-americanos, Blümel *et al.* demonstraram a ocorrência desse distúrbio em 56,8% da amostra.^380^ A regressão logística demonstrou que os FR para a disfunção sexual são diminuição da lubrificação vaginal (mais importante), uso de terapias alternativas para a menopausa e disfunção sexual do parceiro. Maior escolaridade (das mulheres), fidelidade do parceiro e acesso à saúde privada são identificados como protetores.^380^

Utilizando o mesmo instrumento (Índice de Função Sexual Feminina), Cruz, Nina & Figuerêdo investigaram a associação entre disfunção sexual e a intensidade dos sintomas do climatério (avaliada pelo Índice de Kupperman) em mulheres brasileiras de 40-65 anos, acompanhadas em hospital público.^381^ A prevalência da disfunção sexual foi de 58,73%, identificada em 100% das mulheres que apresentavam sintomas climatéricos graves, em 70,59% daquelas com sintomas moderados e apenas em 9,09% daquelas com sintomas leves.^381^

##### E – Outros sintomas

Blümel *et al*.^374^ demonstraram que, em mulheres com SVM, há cinco vezes mais chance de ocorrer desconforto torácico, quatro vezes mais chance de humor depressivo, alterações do sono, disfunção sexual, ansiedade, exaustão física e mental e secura vaginal e três vezes mais chance de irritabilidade, dores musculares/articulares e disfunção urinária.^374^

##### F – Qualidade de vida

Utilizando a mesma amostra,^374,378^ Blümel *et al*.^380^ avaliaram a qualidade de vida e os fatores que interferem negativamente nessa variável. A prevalência de mulheres com sintomas moderados a graves, comprometendo a qualidade de vida, esteve acima de 50% em todos os países, porém Chile e Uruguai apresentaram as maiores pontuações (80,88% e 67,4%, respectivamente). A regressão logística identificou que a qualidade de vida prejudicada em latino-americanas na menopausa foi associada ao uso de terapias alternativas para a menopausa, uso de drogas psiquiátricas, estar na pós-menopausa, ter 49 anos ou mais, viver em grandes altitudes e ter parceiro com disfunção erétil ou ejaculação precoce. Melhor qualidade de vida associou-se a viver em um país com renda mais baixa, usar THM e praticar hábitos saudáveis.^382^

A Tabela 10.2 apresenta a prevalência e os FR ou de proteção para os principais sintomas da menopausa em mulheres da AL.

**Tabela 10.2:** Fatores contribuintes ou de piora e fatores protetores para o aparecimento e maior gravidade de sintomas da menopausa em mulheres da América Latina^373,374,378-380,382.^

SINTOMAS/QUALIDADE DE VIDA	PREVALÊNCIA	FATORES CONTRIBUINTES OU DE PIORA	FATORES PROTETORES
Sintomas vasomotores	45% a 69%; 55%	Graves sintomas psicológicos/urogenitais, menor escolaridade, peri e pós-menopausa natural, nuliparidade, menopausa cirúrgica; morar em grandes altitudes	Uso de THM
Distúrbios do sono	56,6%	Maior idade, doenças crônicas, consumo abusivo de álcool, ansiedade, depressão, SVM, uso de medicamentos (hipnóticos e terapia hormonal)	Maior nível de escolaridade associou-se a menos insônia e melhor qualidade de sono
Dores musculares/articulares	63%	Maior idade, tabagismo, menor escolaridade, SVM, menopausa prematura, pós-menopausa, acompanhamento psiquiátrico e uso de psicotrópicos	Autopercepção de saúde, acesso privado à assistência à saúde e uso de THM associam-se a sintomas menos graves
Disfunção sexual	56,8%	Diminuição da lubrificação vaginal (mais importante), uso de terapias alternativas para a menopausa e disfunção sexual do parceiro	Maior nível de escolaridade, fidelidade do parceiro, acesso à saúde privada são identificados como protetores
Qualidade de vida	50% com comprometimento grave	Uso de terapias alternativas para a menopausa, uso de drogas psiquiátricas, estar na pós-menopausa, ter 49 anos ou mais, viver em grande altitudes, parceiro com disfunção erétil ou ejaculação precoce	Viver em um país com renda mais baixa, usar THM e praticar hábitos saudáveis associam-se a melhor qualidade de vida

SMV: sintomas vasomotores; THM: terapia hormonal da menopausa.

### 10.3. Fatores de Risco Cardiovascular

Os estudos realizados pelo grupo REDLINC desde 2004 até o momento, resultando em 7 projetos de investigação e 18 publicações sobre vários aspectos da menopausa em mulheres latino-americanas, evidenciaram que os FRCV tradicionais e emergentes apresentam a prevalência observada na Tabela 10.3.^383-386^

**Tabela 10.3:** Prevalência dos fatores de risco cardiovascular em mulheres latino-americanas na menopausa.^383-386^

FATOR DE RISCO CARDIOVASCULAR	PREVALÊNCIA (%)
Sedentarismo	63,9
Ansiedade	59,7
Depressão	46,5
Síndrome metabólica	35
Hipertensão Arterial Sistêmica	22,9
Obesidade	18,5
Tabagismo	11,3
Diabetes mellitus	8,6
Menopausa precoce	1,9
Insuficiência ovariana prematura	1,5

A elevada prevalência dos FRCV demonstrada expõe as mulheres peri- e pós-menopáusicas latino-americanas a um maior RCV, o que por um lado deve contribuir para a elevada mortalidade CV atualmente observada nessa região geográfica,^14^ mas, por outro lado, apresenta inúmeras oportunidades de intervenção para o controle dos FR e para a redução do RCV nessa etapa da vida.^13,141,173,387-389^

### 10.4. Tratamento Medicamentoso e Não Medicamentoso

Há evidências de que a prescrição da THM na AL ocorre em 12,5% das mulheres que se encontram nessa etapa da vida, sendo principalmente por via oral (43,7%), seguida da transdérmica (17,7%).^179,347,390^ Nessa investigação, o uso de THM associou-se ao fato de a paciente ter uma percepção positiva sobre essa terapia, estar na pós-menopausa e possuir nível socioeconômico mais elevado.^390^

No grupo que nunca usou THM, 28% relataram a falta de prescrição médica como principal motivo, seguida pela ausência de sintomas (27,8%). Entre aquelas que relataram a falta de prescrição como principal motivo para não usar THM, 30,6% apresentavam sintomas graves de menopausa (escore total da Escala de Avaliação da Menopausa > 16).^390^

O uso de terapias alternativas foi mencionado por 19,5% das mulheres investigadas, 35,1% das quais mencionaram apresentar sintomas graves da menopausa em comparação a 22,5% das usuárias de THM.^390^

Um inquérito (através de questionário autoaplicável e anônimo) realizado com um total de 2.154 ginecologistas (55,5% do sexo masculino, 20,3% docentes e 85% tinham companheiro) em 11 países da AL,^391,392^ 85,3% dos quais responderam à pesquisa (n = 1.837), demonstrou que:

- 85,4% dos ginecologistas responderam que usariam a THM se tivessem sintomas de menopausa (81,8% no caso de ginecologistas do sexo feminino) ou prescreveriam para a sua parceira (88,2% no caso de ginecologistas do sexo masculino).
- A percepção do risco relacionado ao uso da THM (em uma escala de 0 a 10) foi maior entre ginecologistas do sexo feminino do que entre ginecologistas do sexo masculino (4,06 ± 2,09 *versus* 3,83 ± 2,11, respectivamente); os dois principais riscos relatados foram tromboembolismo (mulheres 33,6% *versus* homens 41,4%, respectivamente) e câncer de mama (mulheres 38,5% *versus* homens 33,9%, respectivamente).
- No geral, os ginecologistas relataram prescrever THM para 48,9% de suas pacientes sintomáticas (mulheres 47,3% *versus* homens 50,2%, respectivamente); 86,8% mencionaram terapia não hormonal e 83,8% indicam terapias alternativas para o manejo da menopausa.
- Ginecologistas mais velhos e profissionais acadêmicos prescreveram THM com maior frequência.

Os autores concluíram que, apesar dos ginecologistas da AL terem se mostrado defensores da utilização da THM (para si ou para suas parceiras), isso não se reflete necessariamente em sua prática clínica.^391,392^ Não há ensaios clínicos sobre a utilização de outros medicamentos para o controle de sintomas climatéricos na AL como um todo.

Inúmeras intervenções relacionadas à medicina complementar e alternativa, como as práticas corpo-mente (hipnose, terapias cognitivo-comportamentais, relaxamento, *biofeedback*, meditação, aromaterapia), a utilização de produtos naturais (ervas, vitaminas, minerais, suplementos dietéticos) e outras abordagens (medicina tradicional chinesa, reflexologia, acupuntura, homeopatia) têm sido utilizadas para o controle dos sintomas da menopausa e muitos foram avaliados em ensaios clínicos em outros locais, mas não há estudos específicos para a AL.^393,394^

### 10.5. Desafios e Oportunidades de Intervenção

A combinação do aumento da expectativa de vida ao nascer, a redução na média da idade da menopausa natural e o aumento na prevalência da IOP/menopausa precoce contribui progressivamente para que mais mulheres latino-americanas (51% da população) vivam a experiência da TM e do impacto negativo da mesma em sua qualidade de vida (em todos os domínios), com perdas expressivas no âmbito pessoal, social e econômico, bem como da elevação da morbimortalidade CV (principal causa de morte no mundo atual).^14,365,369-371^

Segundo Faubion e Schufelt,^10^ após a lacuna no tratamento da menopausa deixada pelos resultados de estudos como Heart and Estrogen/Progestin Replacement Study (HERS) e WHI,^1,395^ há uma atenção atual crescente sobre o assunto por parte de serviços de saúde, da mídia (jornais, *blogs* e outros) e até de celebridades, por múltiplas razões, que incluem a projeção de um mercado de $ 600 bilhões envolvendo produtos diversos e a chegada de novas gerações de mulheres (Y, *millennials*) que escolhem não sofrer em silêncio as consequências nefastas da menopausa.^10^

**Figura 10.1:**
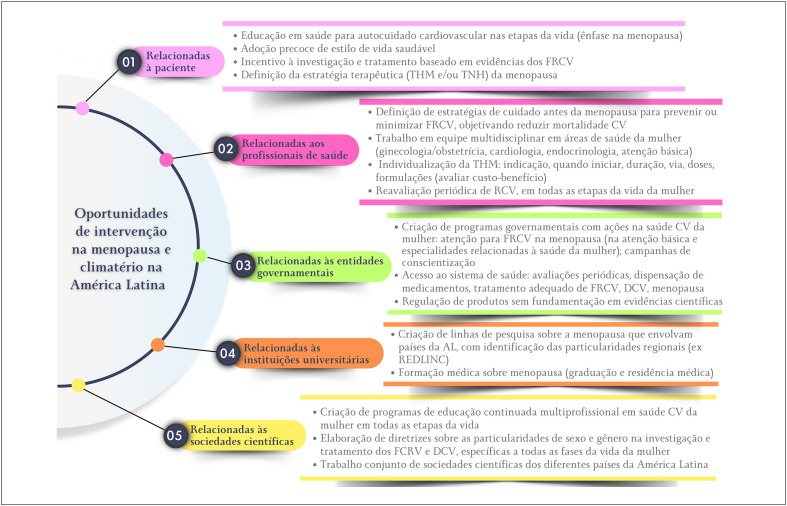
Intervenções possíveis para a melhoria da situação relacionada à menopausa. AL: América Latina; CV: cardiovascular; FRCV: fatores de risco cardiovascular; RCV: risco cardiovascular; THM: terapia hormonal da menopausa; TNH: terapia não hormonal.

Considerando o cenário mundial e da AL acerca da menopausa, a Figura 10.1 lista as inúmeras oportunidades de intervenção para a melhoria da situação atualmente observada.

**Figura 10.2:**
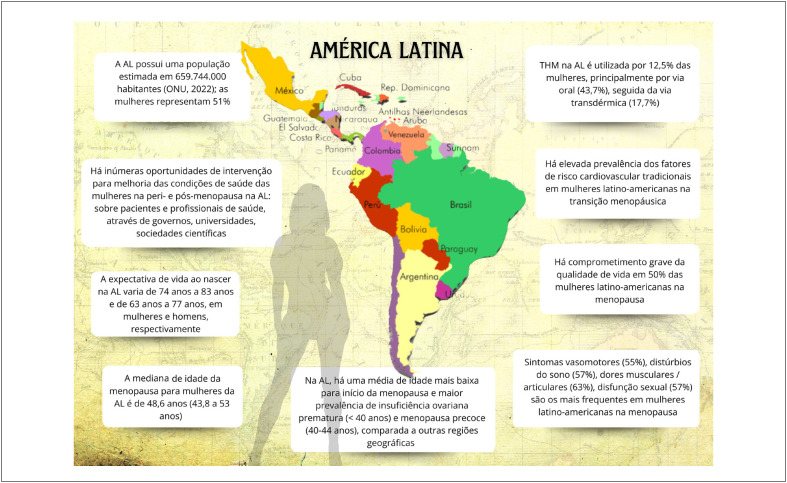
Dados demográficos e sobre a menopausa na América Latina. AL: América Latina; THM: terapia hormonal da menopausa.

A Figura 10.2 resume alguns dados demográficos e sobre a menopausa na América Latina.

## Footnotes

* Nota: A idade de 60 anos deve ser considerada quando a data da última menstruação é desconhecida.

** Nota: A idade de 60 anos deve ser considerada quando a data da última menstruação é desconhecida.245,278-282

*** Nota: antidepressivos (paroxetina, venlafaxina, desvenlafaxina, escitalopram, citalopram), gabapentina e fezolinetante.296-302

## Siglas

Aβ: proteína amiloide β
AHA: American Heart Association
AHCO: anticoncepcional hormonal combinado oral
AL: América Latina
AMH: hormônio antimülleriano
angio-TC: angiotomografia coronariana
anti-TPO: anticorpos anti-peroxidase tireoidiana
ANVISA: Agência Nacional de Vigilância Sanitária
ARIC: *Atherosclerosis Risk in Communities cohort*
ASPREE: *Aspirin in Reducing Events in the Elderly*
ATAC: *Arimidex, Tamoxifen, Alone or in Combination*
AVC: acidente vascular cerebral
CAC: escore de cálcio coronariano
CARDIA: *Coronary Artery Risk Development in Young Adults*
CT: colesterol total
CV: cardiovascular
DAC: doença arterial coronariana
DALYs: anos de vida ajustados por incapacidade (do inglês, *Disability-Adjusted Life Years*)
DCV: doenças cardiovasculares
DHEA: dehidroepiandrosterona
DHEAS: sulfato de dehidroepiandrosterona
DIC: doença isquêmica do coração
DIUH: dispositivo intrauterino hormonal
DLP: dislipidemia
DM: diabetes mellitus
DM2: diabetes tipo 2
EE: etinilestradiol
EMI: espessamento médio-intimal
EP: embolia pulmonar
ERG: escore de risco global
ESPRIT RCT: *Estrogen for the Prevention of Reinfarction Trial*
FA: fibrilação atrial
FC: frequência cardíaca
FEBRASGO: Federação Brasileira das Associações de Ginecologia e Obstetrícia
FPR: fatores potencializadores de risco
FR: fatores de risco
FRCV: fatores de risco cardiovascular
FSH: hormônio folículo-estimulante
GBD: *Global Burden of Disease*
HAS: hipertensão arterial sistêmica
HEAF: *Health and Employment after Fifty Study*
HERS: *Heart and Estrogen/Progestin Replacement Study*
HDL-c: HDL colesterol
HiperSC: hipertireoidismo subclínico
HipoSC: hipotireoidismo subclínico
IAM: infarto agudo do miocárdio
IC: intervalo de confiança
IL: interleucina
IM: infarto do miocárdio
IMC: índice de massa corporal
IMS: *International Menopause Society*
IOP: insuficiência ovariana prematura
ITB: índice tornozelo-braquial
LDL-c: LDL colesterol
Lp(a): lipoproteína (a)
NAMS: *North American Menopause Society*
MESA: *Multi-Ethnic Study of Atherosclerosis*
NO: óxido nítrico
OMS: Organização Mundial da Saúde
OR: *odds ratio*
PA: pressão arterial
PEPI: *Postmenopausal Estrogen/Progestin Interventions*
POP: pílulas orais de progesterona
Qt: quimioterapia
RCV: risco cardiovascular
RR: risco relativo
SBC: Sociedade Brasileira de Cardiologia
SCA: síndrome coronariana aguda
SIAC: *Sociedad Interamericana de Cardiología*
SM: síndrome metabólica
SOBRAC: Associação Brasileira de Climatério
SOP: síndrome dos ovários policísticos
SRAA: sistema renina-angiotensina-aldosterona
STRAW: *Stages of Reproductive Aging Workshop*
SVM: sintomas vasomotores
SWAN: *Women's Health Across the Nation*
TEV: tromboembolismo venoso
TFGe: taxa de filtração glomerular estimada
TG: triglicerídeos
THAG: terapia hormonal para afirmação de gênero
THM: terapia hormonal da menopausa
TM: transição menopáusica
TVP: trombose venosa profunda
VE: ventrículo esquerdo
VLDL-c: VLDL colesterol
WISE: *Women's Ischemia Syndrome Evaluation*
WHI: *Women's Health Initiative*

## Apêndice

### Objetivo 1

A presente revisão tem como questão norteadora: Quais os benefícios, os danos, as indicações e as contraindicações da THM, quanto aos sinais e sintomas do climatério/menopausa em mulheres com fatores de risco cardiovascular ou doenças cardiovasculares? Usando o acrônimo População, Intervenção, Comparação e Desfecho (*Outcomes*) (PICO), tem-se P – mulheres com fatores de risco cardiovascular ou doenças cardiovasculares, Intervenção – Terapia de Reposição Hormonal, Comparação – não se aplica, *Outcomes* – benefícios, danos, indicações e contraindicações.

A estratégia de busca orientou-se pelo acrônimo e os termos padronizados e as palavras-chave da pesquisa foram identificados nos vocabulários controlados: Descritores em Ciências da Saúde (DECS) via Portal Regional da Biblioteca Virtual em Saúde, Medical Subject Heading (MESH) por meio do PubMed e Emtree (Embase subject headings) da base de dados EMBASE (Elsevier). Realizou-se uma busca preliminar para identificar termos adicionais nos títulos, resumos e palavras-chaves dos artigos e, em seguida, uma estratégia de busca definitiva foi aplicada em todas as bases.

A estratégia de busca foi aplicada e adaptada no dia 23 de dezembro de 2023 nas bases de dados: LILACS, BDENF, WPRIM, CUMED OR LIPECS OR BINACIS do Portal Regional da Biblioteca Virtual em Saúde, MEDLINE/PubMed da National Library of Medicine (NLM). No Portal de Periódicos da Capes: EMBASE e SCOPUS (Elsevier), CINAHL, Academic Search Premier (Ebsco), Web of Science (Clarivate). Acrescentou-se a Scientific Electronic Library Online (Scielo). Apresentam-se as estratégias de busca da Cochrane Library e Pubmed (Tabela 1 e 2)

**Tabela 1:** Cochrane Library

Cochrane Library	Nº
#1 MeSH descriptor: [Women] explode all trees 1236	35
#2 female* OR woman OR women OR female 993875	
#3 #1 OR #2 993875	
#4 MeSH descriptor: [Heart Failure] explode all trees 14720	
#5 MeSH descriptor: [Cardiovascular Diseases] explode all trees 152906	
#6 MeSH descriptor: [Heart Diseases] explode all trees 72792	
#7 MeSH descriptor: [Coronary Disease] explode all trees 18548	
#8 MeSH descriptor: [Coronary Artery Disease] explode all trees 9532	
#9 MeSH descriptor: [Arrhythmias, Cardiac] explode all trees 14339	
#10 MeSH descriptor: [Arrhythmias, Cardiac] explode all trees 14339	
#11 "Heart Failure" OR "Cardiac Failure" OR "Heart Decompensation" OR "Myocardial Failure" OR "cardiac backward failure" OR "cardiac decompensation" OR "cardiac incompetence" OR "cardiac insufficiency" OR "cardiac stand still" OR "cardial decompensation" OR "cardial insufficiency" OR "heart insufficiency" OR "decompensation cordis" OR "heart backward failure" OR "heart decompensation" OR "heart incompetence" OR "insufficientia cardis" OR "myocardial insufficiency" OR "Cardiovascular Diseases" OR "Cardiac Event" OR "Cardiac Events" OR "Cardiovascular Disease" OR "Heart Diseases" OR "Cardiac Disease" OR "Cardiac Diseases" OR "Cardiac Disorder" OR "Cardiac Disorders" OR "Heart Disease" OR "Heart Disorder" OR "Heart Disorders" OR "Coronary Disease" OR "Coronary Diseases" OR "Coronary Heart Disease" OR "Coronary Heart Diseases" OR "Coronary Artery Disease" OR "Arterioscleroses, Coronary" OR "Coronary Arterioscleroses" OR "Coronary Arteriosclerosis" OR "Coronary Artery Diseases" OR "Coronary Atheroscleroses" OR "Coronary Atherosclerosis" OR "Arrhythmias, Cardiac" OR Arrhythmia OR Arrythmia OR "Cardiac Arrhythmia" OR "Cardiac Arrhythmias" OR "Cardiac Dysrhythmia" OR "Myocardial Infarction" OR "Cardiovascular Stroke" OR "Cardiovascular Strokes" OR "Heart Attack" OR "Heart Attacks" OR "Myocardial Infarct" OR "Myocardial Infarctions" OR "Myocardial Infarcts" 142558	
#12 #4 OR #5 OR #6 OR #7 OR #8 OR #9 OR #10 #11 152906	
#13 MeSH descriptor: [Hormone Replacement Therapy] explode all trees 3536	
#14 MeSH descriptor: [Estrogen Replacement Therapy] explode all trees 2485	
#15 "Hormone Replacement Therapy" OR "Hormone Replacement Therapies" OR "Estrogen Replacement Therapy" OR "Estrogen Progestin Combination Therapy" OR "Estrogen Progestin Replacement Therapy" OR "Estrogen Replacement" OR "Estrogen Replacement Therapies" OR "Estrogen Replacements" OR "Postmenopausal Hormone Replacement Therapy" OR Estrogen 17111	
#16 #13 OR #14 OR #15 17111	
#17 MeSH descriptor: [Primary Prevention] explode all trees 6617	
#18 "Primary Prevention" OR "Primary Disease Prevention" OR "Primordial Prevention" OR "Primary Prevention" 5378	
#19 #17 OR #18 10597	
#20 #3 AND #12 AND #16 AND #19 35	

Fonte: Cochrane Library

**Tabela 2:** Medline/PubMed

Search	Query	Results
#1	Search: "Women"[mh] OR Woman OR Women	1,717,025
#2	Search: Menopause[mh] OR Menopause	96,061
#3	Search: "Hormone Replacement Therapy"[mh] OR Hormone Replacement Therap* OR "Estrogen Replacement Therapy"[mh] OR Estrogen Progestin Combination Therapy OR Estrogen Progestin Replacement Therapy OR Estrogen Replacement OR Estrogen Replacement Therapies OR Estrogen Replacement* OR Postmenopausal Hormone Replacement Therapy OR Estrogen	312,110
#4	Search: "Primary Prevention"[mh] OR Primary Disease Prevention* OR Primordial Prevention* OR Primary Prevention	377,833
#5	Search: "Heart Failure"[mh] OR Heart Failure OR Cardiac Failure OR Heart Decompensation OR Myocardial Failure OR cardiac backward failure OR cardiac decompensation OR cardiac incompetence OR cardiac insufficiency OR cardiac stand still OR cardial decompensation OR cardial insufficiency OR heart insufficiency OR decompensatio cordis OR heart backward failure OR heart decompensation OR heart incompetence OR insufficientia cardis OR myocardial insufficiency OR "Cardiovascular Diseases"[mh] OR Cardiac Event* OR Cardiovascular Disease* OR "Heart Diseases"[mh] OR Cardiac Disease* OR Cardiac Disorder* OR Heart Disease* OR Heart Disorder* OR Cardiac OR "Coronary Disease"[mh] OR Coronary Heart Disease* OR Coronary Artery Disease* OR Coronary Arterioscleros* OR Coronary Atheroscleros* OR "Arrhythmias, Cardiac"[mh] OR Arrhythmia* OR Arrythmia OR Cardiac Arrhythmia* OR "Cardiac Dysrhythmia" OR Atheroscleros* OR "Myocardial Infarction"[mh] OR Cardiovascular Stroke* OR Heart Attack* OR Myocardial Infarct* OR Cardiovascular	4,429,382
#6	Search: #1 AND #2 AND #3 AND #4 AND #5	422

Fonte: MEDLINE/Pubmed

As referências recuperadas nas buscas foram agrupadas por base no EndNote (Clarivate Analytics, PA, USA), *software* gerenciador de referências e as duplicatas removidas. Em seguida, as referências foram importadas no *software* Intelligent Systematic Review (Rayyan) para seleção de acordo com os critérios de inclusão. As fontes selecionadas na primeira triagem de título e resumo foram recuperadas na íntegra. As referências potencialmente relevantes foram avaliadas por meio de leitura para confirmação da inclusão ou exclusão. Os resultados do processo de busca, identificação, seleção e inclusão serão descritos no *Preferred Reporting Items for Systematic reviews and Meta-Analyses* (PRISMA).

### Objetivo 2

A presente revisão tem como questão norteadora: Quais os benefícios, os danos, as indicações e as contraindicações da THM em mulheres? Usando o acrônimo População, Intervenção, Comparação e Desfecho (*Outcomes*) (PICO), tem-se P – mulheres, Intervenção – Terapia de Reposição Hormonal, Comparação – não se aplica, *Outcomes* – benefícios, danos, indicações e contraindicações.

A estratégia de busca orientou-se pelo acrônimo e os termos padronizados e as palavras-chave da pesquisa foram identificadas nos vocabulários controlados: Descritores em Ciências da Saúde (DECS) via Portal Regional da Biblioteca Virtual em Saúde, Medical Subject Heading (MESH) por meio do PubMed e Emtree (Embase subject headings) da base de dados EMBASE (Elsevier). Realizou-se uma busca preliminar para identificar termos adicionais nos títulos, resumos e palavras-chaves dos artigos e, em seguida, uma estratégia de busca definitiva foi aplicada em todas as bases.

A estratégia de busca foi aplicada e adaptada no dia 1 de janeiro de 2024 nas bases de dados: LILACS, IBECS, WPRIM e BIGG do Portal Regional da Biblioteca Virtual em Saúde, MEDLINE/PubMed da National Library of Medicine (NLM). No Portal de Periódicos da Capes: EMBASE e SCOPUS (Elsevier), CINAHL, Academic Search Premier (Ebsco), Web of Science (Clarivate). Acrescentaram-se Scientific Electronic Library Online (Scielo) e Epistemonikos. Apresentam-se as estratégias de busca da Cochrane Library e PubMed (Tabela 3 e 4).

**Tabela 3:** Cochrane Library

Cochrane Library	Nº
ID Search Hits	109
#1 MeSH descriptor: [Women] explode all trees 1242	
#2 female* OR woman OR women OR female 996379	
#3 #1 OR #2 996379	
#4 MeSH descriptor: [Hormone Replacement Therapy] explode all trees 3537	
#5 MeSH descriptor: [Estrogen Replacement Therapy] explode all trees 2486	109
#6 "Hormone Replacement Therapy" OR "Hormone Replacement Therapies" OR "Estrogen Replacement Therapy" OR "Estrogen Progestin Combination Therapy" OR "Estrogen Progestin Replacement Therapy" OR "Estrogen Replacement" OR "Estrogen Replacement Therapies" OR "Estrogen Replacements" OR "Postmenopausal Hormone Replacement Therapy" OR Estrogen 17128	
#7 #4 OR #5 OR #6 17128	
#8 MeSH descriptor: [Clinical Protocols] explode all trees 23966	
#9 MeSH descriptor: [Consensus] explode all trees 566	
#10 MeSH descriptor: [Guideline] explode all trees 1015	
#11 MeSH descriptor: [Meta-Analysis] explode all trees 0	
#12 MeSH descriptor: [Systematic Review] explode all trees 426	
#13 MeSH descriptor: [Health Planning Guidelines] explode all trees 28	
#14 MeSH descriptor: [Practice Guidelines as Topic] explode all trees 2750	
#15 "Clinical protocols" OR Consensus OR "Critical pathways" OR "Guidelines as topic" OR "Practice guidelines as topic" OR "Health planning guidelines" OR "Clinical Decision Rules" OR guideline* OR "practice guideline" OR "consensus development conference" OR "position statement" OR "policy statement" OR "practice parameter" OR "best practice" OR standards OR recommendat* OR "treatment guideline" OR "clinical guideline" OR "guideline recommendation" OR "systematic" OR "meta-analysis" OR "meta-analysis as topic" OR "meta analy" OR metanaly OR metaanaly OR "met analy" OR "integrative research" OR "integrative review" OR "integrative overview" OR "research integration" OR "research overview" OR "collaborative review" OR "collaborative overview" OR "systematic review" OR "systematic reviews as topic" OR "systematic review" 155064	
#16 #8 OR #9 OR #10 OR #11 OR #12 OR #13 OR #14 OR #15 172718	
#17 #3 AND #7 AND #16 2077	
#18 "Breast Neoplasms" OR "Breast cancer" OR "Breast tumor" OR "breast neoplasm" OR animal*OR "In Vitro" OR mice OR mouse OR rat OR rats OR murine OR rodent* OR beagle 63541	
#19 #3 AND #7 AND #16 NOT #18 566	

Fonte: Cochrane Library

**Tabela 4:** Medline/PubMed

Search	Query	Results
#1	Search: "Women"[mh] OR Woman OR Women	1,717,025
#2	Search: "Hormone Replacement Therapy/adverse effects"[Majr] OR "Hormone Replacement Therapy/instrumentation"[Majr] OR "Hormone Replacement Therapy/methods"[Majr] OR "Hormone Replacement Therapy/trends"[Majr] OR "Estrogen Replacement Therapy/adverse effects"[Mesh] OR "Estrogen Replacement Therapy/classification"[Mesh] OR "Estrogen Replacement Therapy/instrumentation"[Mesh] OR "Estrogen Replacement Therapy/methods"[Mesh] OR "Estrogen Replacement Therapy/standards"[Mesh] OR "Estrogen Replacement Therapy/trends"[Mesh] OR "Hormone Replacement Therapy"[mh] OR Hormone Replacement Therap* OR "Estrogen Replacement Therapy"[mh] OR Estrogen Progestin Combination Therapy OR Estrogen Progestin Replacement Therapy OR Estrogen Replacement OR Estrogen Replacement Therapies OR Estrogen Replacement* OR Postmenopausal Hormone Replacement Therapy OR Estrogen	312,110
#3	Search: "Clinical protocols"[mh] OR "Consensus"[mh] OR "Consensus development conferences as topic"[mh] OR "Critical pathways"[mh] OR "Guidelines as topic" [Mesh:NoExp] OR "Practice guidelines as topic"[mh] OR "Health planning guidelines"[mh] OR "Clinical Decision Rules"[mh] OR "guideline"[pt] OR "practice guideline"[pt] OR "consensus development conference"[pt] OR "consensus development conference, NIH"[pt] OR "position statement*"[tiab] OR "policy statement*"[tiab] OR "practice parameter*"[tiab] OR "best practice*"[tiab] OR standards[TI] OR guideline[TI] OR guidelines[TI] OR standards[ot] OR guideline[ot] OR guidelines[ot] OR guideline*[cn] OR standards[cn] OR consensus*[cn] OR recommendat*[cn] OR "practice guideline*"[tiab] OR "treatment guideline*"[tiab] OR CPG[tiab] OR CPGs[tiab] OR "clinical guideline*"[tiab] OR "guideline recommendation*"[tiab] OR consensus*[tiab] OR "systematic"[filter] OR "meta-analysis"[pt] OR "meta-analysis as topic"[mh] OR "meta analy*"[tw] OR metanaly*[tw] OR metaanaly*[tw] OR "met analy*"[tw] OR "integrative research"[tiab] OR "integrative review*"[tiab] OR "integrative overview*"[tiab] OR "research integration*"[tiab] OR "research overview*"[tiab] OR "collaborative review*"[tiab] OR "collaborative overview*"[tiab] OR "systematic review"[pt] OR "systematic reviews as topic"[mh] OR "systematic review*"[tiab]	1,256,669
#4	Search: english[Filter] OR portuguese[Filter] OR spanish[Filter]	32,382,331
#5	Search: 2019:2024[pdat]	7,580,602
#6	Search: "Breast Neoplasms"[majr] OR animals[mh] OR animal*[tiab] OR "In Vitro Techniques"[mh] OR mice[tw] OR mouse[tw] OR rat[tiab] OR rats[tiab] OR murine[tw] OR rodent*[tw] OR beagle[tw]	27,333,977
#7	Search: #1 AND #2 AND #3 AND #4 AND #5 AND #6	1,035

Fonte: MEDLINE/Pubmed

Os limites da busca foram idioma português, inglês e espanhol, ano de publicação a partir de 2019, tipo de estudo consensos, revisões sistemáticas, protocolos clínicos, *guidelines*, meta-análise, recomendações.

As referências recuperadas nas buscas foram agrupadas por base no EndNote (Clarivate Analytics, PA, USA), *software* gerenciador de referências e as duplicatas removidas. Em seguida, as referências foram importadas no *software* Intelligent Systematic Review (Rayyan) para seleção de acordo com os critérios de inclusão. As fontes selecionadas na primeira triagem de título e resumo foram recuperadas na íntegra. As referências potencialmente relevantes foram avaliadas por meio de leitura para confirmação da inclusão ou exclusão. Os resultados do processo de busca, identificação, seleção e inclusão serão descritos no *Preferred Reporting Items for Systematic reviews and Meta-Analyses* (PRISMA).
